# OTUB1 inhibits autophagy-dependent ferroptosis in hepatocellular carcinoma by stabilizing p62 via non-canonical deubiquitination

**DOI:** 10.1038/s41420-026-03241-5

**Published:** 2026-07-22

**Authors:** Pengcheng Zhao, Yongxin Wang, Nima Saeidi, Ping Zhang

**Affiliations:** 1 Department of Hepatobiliary Pancreas and Spleen Surgery, General Surgery Center, The First Hospital of Jilin University (the First Bethune Hospital of Jilin University), Changchun, China; 2 Division of Gastrointestinal and Oncologic Surgery, Department of Surgery, Massachusetts General Hospital, Boston, MA USA; 3 Center for Engineering in Medicine and Surgery, Department of Surgery, Massachusetts General Hospital, Boston, MA USA; 4 Shriners Hospital for Children, Boston, MA USA; 5 Harvard Medical School, Boston, MA USA

**Keywords:** Cancer, Cancer

## Abstract

Ubiquitination plays a critical role in hepatocellular carcinoma (HCC) pathogenesis and is closely linked to ferroptosis. This study investigates the function of OTUB1, a deubiquitinase overexpressed in HCC, in regulating autophagy-dependent ferroptosis. Using integrated bioinformatics, biochemical assays, and xenograft models, we demonstrated that OTUB1 is upregulated in HCC and correlates with poor prognosis. Functionally, OTUB1 knockdown suppressed proliferation and induced ferroptosis, whereas its overexpression promoted malignancy. Mechanistically, OTUB1 competitively binds to p62/SQSTM1, thereby suppressing autophagic flux. Crucially, we identified that OTUB1 stabilizes p62 via non-canonical, D88-dependent deubiquitination by specifically removing K48-linked polyubiquitin chains. This stabilization hinders the initiation of autophagy-dependent ferroptosis. Pharmacological inhibition of autophagy abrogated the ferroptosis induced by OTUB1 knockdown. Furthermore, inhibiting OTUB1 sensitized HCC tumors to Lenvatinib, resulting in synergistic anti-tumor efficacy in vivo. Collectively, these findings reveal that OTUB1 drives HCC progression by stabilizing p62 to block autophagy-dependent ferroptosis. Targeting the OTUB1-p62 axis represents a novel therapeutic strategy to overcome drug resistance and improve outcomes in advanced HCC.

## Introduction

Hepatocellular carcinoma (HCC) is a leading cause of cancer mortality worldwide [1, 2]. Due to the insidious nature of its onset, over 50% of patients are diagnosed at advanced stages, precluding curative surgical resection. Consequently, systemic therapies, including chemotherapy and immunotherapy, constitute the primary treatment modalities [3, 4]. Lenvatinib, an oral multi-target tyrosine kinase inhibitor affecting VEGFR and FGFR pathways, acts as a first-line therapy for unresectable HCC [5]. However, the clinical benefit of Lenvatinib is frequently compromised by the development of acquired resistance [6]. Therefore, elucidating the molecular mechanisms driving HCC progression and drug resistance is urgently needed to identify novel therapeutic targets and develop effective combination strategies.

Recent evidence implicates ferroptosis—an iron-dependent form of regulated cell death characterized by lipid peroxidation—as a critical determinant of HCC progression and therapeutic response [7]. The metabolic reprogramming inherent to tumor cells results in reactive oxygen species (ROS) accumulation, glutathione (GSH) depletion, and iron overload, rendering them intrinsically vulnerable to ferroptosis [8, 9]. Several regulators, such as SOCS2 and GSTZ1, have been shown to modulate ferroptosis sensitivity in HCC via the SLC7A11 or NRF2 pathways [10, 11]. Notably, autophagy, a lysosomal degradation pathway, can synergistically drive ferroptosis through mechanisms such as ferritinophagy (NCOA4-mediated), lipophagy, and degradation of cystine transporters [12]. While specific deubiquitinases like USP24 have been linked to autophagy-dependent ferroptosis in HCC [13], the broader regulatory network governing this interplay remains poorly defined.

Aberrant ubiquitination is a hallmark of HCC pathogenesis [14]. OTUB1, a deubiquitinase of the ovarian tumor (OTU) family, promotes tumorigenesis in various cancers by stabilizing key oncoproteins, including c-Maf, MYC, and FOXM1 [15–17]. Although OTUB1 is known to promote HCC malignancy via RACK1 stabilization [14] and has been reported to inhibit ferroptosis in gastric and nasopharyngeal carcinomas [18–20], its specific role in regulating autophagy-dependent ferroptosis in HCC remains unexplored. Intriguingly, OTUB1 has been implicated in this process in skeletal muscle [21], suggesting a potential conserved mechanism that warrants investigation in the context of liver cancer.

Here, we report that OTUB1 drives HCC progression by stabilizing p62 to suppress autophagy-dependent ferroptosis. Importantly, we demonstrate that pharmacological inhibition of OTUB1 sensitizes HCC tumors to Lenvatinib in vivo. Our findings thus characterize the OTUB1-p62 axis as a key mechanism of ferroptosis evasion and a promising target for combination therapy.

## Results

### OTUB1 is upregulated in HCC and promotes malignant progression

**Fig. 1:**
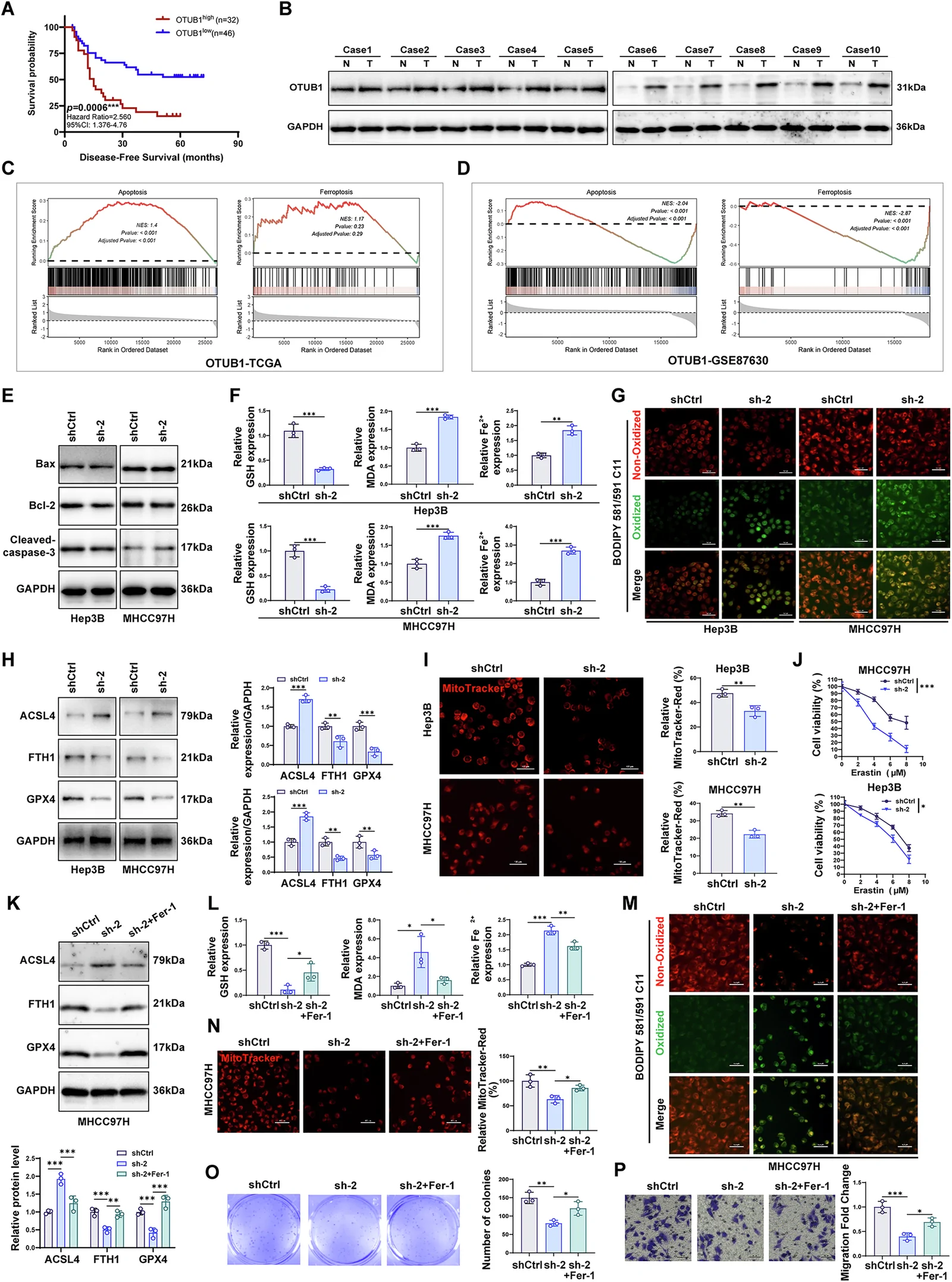
OTUB1 is upregulated in HCC, correlates with poor prognosis, and its knockdown induces ferroptosis. **A** Kaplan-Meier curves of disease-free survival (DFS) for the in-house cohort of 78 HCC patients, stratified by OTUB1 expression levels. **B** Western blot analysis of OTUB1 protein expression in paired tumor and adjacent non-tumorous liver tissues from 10 HCC patients. GSEA in TCGA-LIHC (**C**) and GSE87630 (**D**) cohorts showing enrichment of apoptosis and ferroptosis pathways. **E, H, K** Western blot analysis of apoptosis- and ferroptosis-related proteins in Hep3B and MHCC97H cells (*n* = 3). **F, L** The relative values of GSH, MDA, and Fe^2+^ concentrations in indicated cells (*n* = 3). **G, M** Lipid peroxidation assessed by BODIPY 581/591 C11 staining. Scale bar: 100 μm. **I, N** Mitochondrial membrane potential detected by MitoTracker Red in the indicated cells (*n* = 3). Scale bar: 100 μm. **J** Cell viability after Erastin treatment (2-10 μM) assessed by CCK-8 assay (*n* = 3). **O** The proliferation of cells was assessed using colony formation assays (*n* = 3). **P** Migration abilities of cells were evaluated with Transwell assays (*n* = 3). Scale bar: 100 μm. ns, *P* > 0.05; *, *P* < 0.05; **, *P* < 0.01; ***, *P* < 0.001.

To investigate OTUB1’s role in HCC, we analyzed TCGA and GEO (GSE87630) datasets, which revealed significant OTUB1 mRNA upregulation in tumors versus adjacent tissues, correlating with poor prognosis (Fig. S1A–C). To validate these clinical implications, we assessed an in-house cohort of 78 HCC patients. This analysis confirmed elevated OTUB1 expression in tumor tissues (*p* < 0.001, Table S1), and linked high OTUB1 levels to aggressive features, including multiple tumor nodules, recurrence, and increased ALT and GGT serum levels (Table S2). Crucially, Kaplan-Meier analysis demonstrated that patients with high OTUB1 expression experienced significantly shorter disease-free survival (Fig. 1A). Consistent with the transcriptional data, OTUB1 protein was markedly upregulated in paired clinical HCC tissues (Figs. 1B and S1D), a finding further corroborated by immunohistochemistry data from The Human Protein Atlas (Fig. S1E). Collectively, these data further support the association between elevated OTUB1 expression and adverse clinical outcomes in HCC, establishing OTUB1 as a biomarker of aggressive HCC.

We then assessed OTUB1 expression in HCC cell lines, noting higher levels in Hep3B and MHCC97H than in HepG2 and Huh7 or the normal LX-2 line (Fig. S1F, G). Stable OTUB1-knockdown models were generated in Hep3B and MHCC97H cells (sh-2 showing optimal efficiency; Fig. S1H), while OTUB1-overexpressing lines were established in HepG2 and Huh7 (Fig. S1I). Functional assays showed that OTUB1 knockdown suppressed proliferation (colony formation and CFDA-SE retention; Fig. S2A, B), increased apoptosis (Fig. S2C), impaired migration (Fig. S2D), reduced adhesion, and modulated EMT markers (increased E-cadherin, decreased N-cadherin and Vimentin; Fig. S2E, F). Conversely, OTUB1 overexpression enhanced proliferation, migration, and invasion (Fig. S2G–L). These in vitro results demonstrate that OTUB1 promotes malignant phenotypes in HCC, including proliferation, migration, and EMT.

### OTUB1 promotes HCC malignant progression by suppressing ferroptosis

To elucidate the molecular mechanism underlying OTUB1-mediated HCC malignancy, we performed GSEA using TCGA and GSE87630 datasets. The analysis linked OTUB1 to both apoptosis and ferroptosis; however, OTUB1 knockdown showed negligible effects on apoptosis, prompting us to focus on ferroptosis (Fig. 1C–E). Investigating ferroptotic biochemical markers, we found that OTUB1 depletion increased MDA, Fe^2+^, and lipid peroxidation while reducing GSH content (Figs. 1F, G and S3A). Molecularly, this was accompanied by upregulated ACSL4 and downregulated GPX4 and FTH1, alongside impaired mitochondrial membrane potential (Fig. 1H, I). Conversely, OTUB1 overexpression in HepG2 and Huh7 cells suppressed these ferroptotic hallmarks and alleviated mitochondrial damage (Fig. S3B–F). Functionally, OTUB1 depletion sensitized HCC cells to Erastin-induced cytotoxicity, whereas overexpression conferred resistance (Figs. 1J and S3G). To validate that OTUB1 promotes progression via ferroptosis inhibition, we performed rescue assays. The inhibitor Fer-1 reversed the knockdown-induced ferroptotic markers and mitochondrial damage (Figs. 1K–N and S3H), thereby restoring cell proliferation and migration (Fig. 1O, P). Consistently, Erastin treatment abolished the protective effects of OTUB1 overexpression on mitochondrial integrity and cellular functions (Fig. S3I–N). Collectively, these findings demonstrate that OTUB1 facilitates HCC progression primarily by suppressing ferroptosis.

### OTUB1 suppresses autophagy-dependent ferroptosis by competitively binding p62

**Fig. 2:**
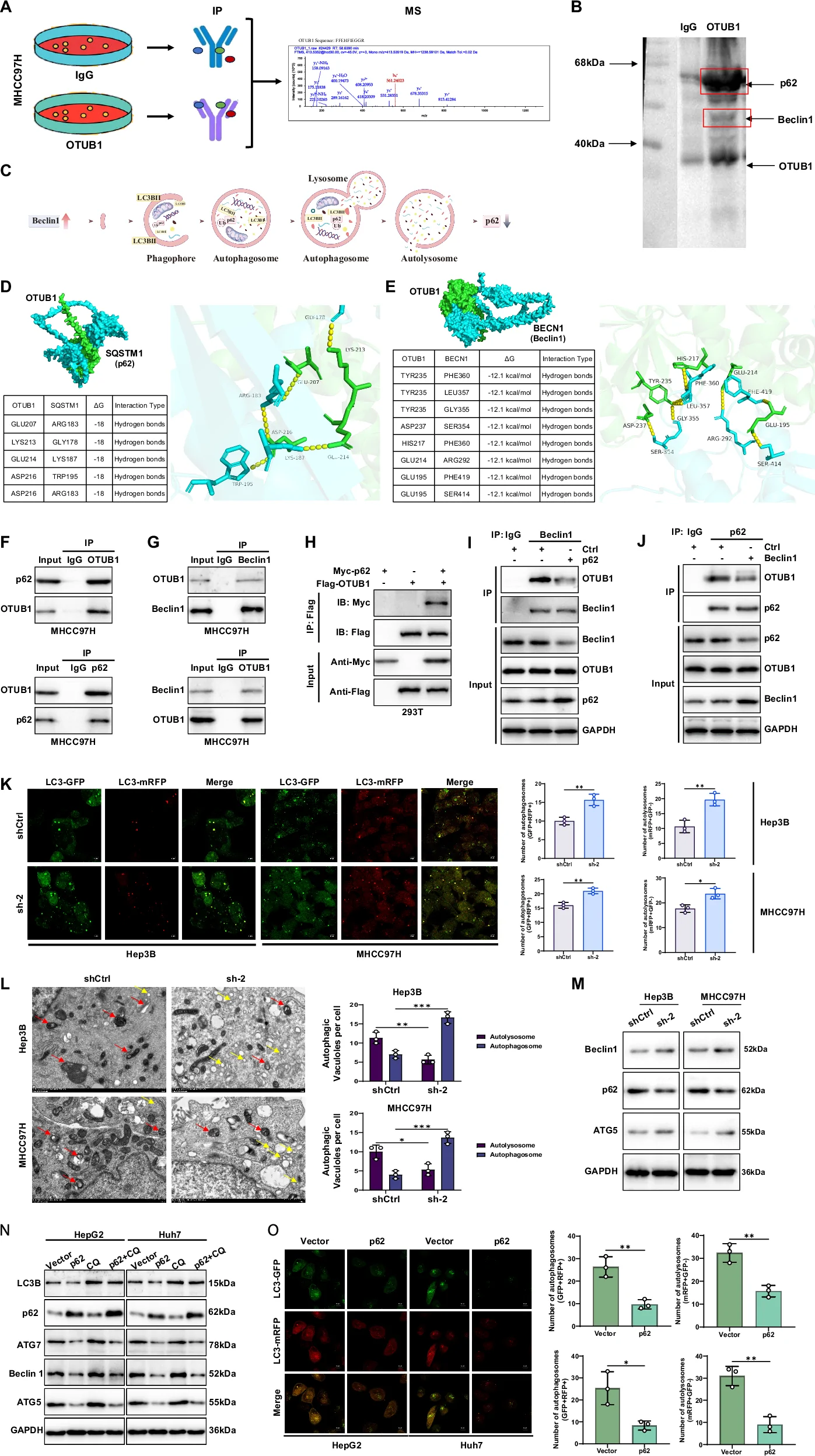
OTUB1 inhibits autophagy by competitively binding to p62. Workflow of OTUB1 interactor identification by IP-MS **A** three replicates) and silver staining (**B**). **C** Schematic diagram of autophagy. AlphaFold3 prediction of OTUB1 interactions with p62 (**D**) and Beclin1 (**E**). Reciprocal Co-IP confirming OTUB1 binding to p62 (**F**) and Beclin1 (**G**), with IgG as control. **H** Validation of OTUB1–p62 interaction by Flag-OTUB1 and Myc-p62 co-immunoprecipitation in HEK293T cells. Competitive Co-IP showing reduced OTUB1–Beclin1 binding upon p62 overexpression (**I**), and reduced OTUB1–p62 binding upon Beclin1 overexpression (**J**). **K** Confocal microscopy of mRFP-GFP-LC3 puncta in Hep3B and MHCC97H cells with OTUB1 knockdown, and quantification of mRFP-GFP-LC3 puncta per cell. Yellow puncta (GFP^+^/mRFP^+^) represent autophagosomes; red puncta (GFP^-^/mRFP^+^) represent autolysosomes (*n* = 3). Scale bar: 10 μm. **L** Transmission electron microscopy showing autophagosomes and autolysosomes (*n* = 3). Scale bar: 1 μm. **M** Western blot analysis of ATG5, p62, and Beclin1 in indicated cells (*n* = 3). **N** Western blot analysis of LC3B, p62, ATG7, Beclin1, and ATG5 in cells treated with Vector, p62 overexpression, CQ, or p62 plus CQ (*n* = 3). **O** Confocal microscopy and quantification of mRFP-GFP-LC3 puncta under Vector or p62 overexpression conditions. The number of autophagosomes (yellow puncta, GFP^+^/mRFP^+^) and autolysosomes (red puncta, GFP^-^/mRFP^+^) per cell was independently quantified to assess the blockade of autophagic flux (*n* = 3). Scale bar: 10 μm. *, *P* < 0.05; **, *P* < 0.01; ***, *P* < 0.001.

To delineate the molecular mechanism by which OTUB1 regulates ferroptosis, we performed IP-MS and identified p62 and Beclin1 as OTUB1-interacting partners (Fig. 2A, B).Given the established role of ferroptosis as an autophagy-dependent cell death process [22, 23], and the distinct functions of Beclin1 (promoting autophagosome initiation) and p62 (SQSTM1, an autophagy receptor whose degradation reflects active autophagic flux) (schematic in Fig. 2C), we focused on these two key autophagy regulators. AlphaFold3 structural prediction showed that p62 and Beclin1 compete for the same GLU214 residue on OTUB1 (Fig. 2D, E). Further validated the OTUB1-p62 and OTUB1-Beclin1 interaction via reciprocal Co-IP (Fig. 2F, G). Exogenous Co-IP further verified the interaction between OTUB1 and p62 (Fig. 2H). Specifically, p62 overexpression reduced OTUB1-Beclin1 binding (Fig. 2I), while Beclin1 overexpression reduced OTUB1-p62 binding (Fig. 2J). To assess the impact on autophagic activity, we monitored flux using mRFP-GFP-LC3 reporter assays and TEM. By quantifying GFP^+^/mRFP^+^ (yellow) puncta representing autophagosomes and GFP^-^/mRFP^+^ (red) puncta representing autolysosomes, we observed that OTUB1 knockdown significantly increased both autophagosome and autolysosome numbers per cell (Fig. 2K), indicating enhanced autophagic flux. TEM further confirmed the accumulation of autophagosomes in OTUB1-knockdown cells (Fig. 2L). Consistently, OTUB1 knockdown upregulated Beclin1 and downregulated p62 and ATG5, whereas OTUB1 overexpression produced the opposite effects (Figs. 2M and S4A, B). RT-qPCR was performed. Modulation of OTUB1 did not significantly alter SQSTM1 (p62), ATG5, or BECN1 mRNA levels (Fig. S4C–H), confirming that OTUB1 regulates these targets post-translationally. Notably, p62 accumulation is widely regarded as a marker of impaired autophagic flux, while our model proposes that p62 stabilization actively suppresses autophagy initiation. To clarify this causal relationship, cells were transfected with p62 plasmids with or without the lysosomotropic agent chloroquine (CQ). We found that p62 overexpression alone significantly reduced LC3B, Beclin1, and ATG5 protein levels. This suppressive effect was maintained even in the presence of CQ. (Figs. 2N and S4I). Consistent with the biochemical results, the mRFP-GFP-LC3 tandem reporter assay further confirmed the autophagy-suppressive effect of p62 (Fig. 2O). Thus, p62 stabilization actively suppresses autophagic flux.

To validate that OTUB1 inhibits ferroptosis via autophagy suppression, we examined these pathways under Erastin challenge. Erastin treatment reversed the OTUB1 overexpression-induced suppression of autophagy markers (LC3-Ⅱ, Beclin1, ATG5) and accumulation of p62 (Fig. S4J–O). Crucially, blocking autophagy with CQ or overexpressing p62 rescued the ferroptotic phenotypes induced by OTUB1 depletion (Fig. S5A–F). To further solidify this causal link with genetic evidence, we silenced the core autophagy component ATG5 in OTUB1-knockdown cells. Consistent with the pharmacological results, genetic ablation of autophagy significantly reversed OTUB1 depletion-induced ferroptotic phenotypes, including restored GSH content, reduced MDA and Fe^2+^ accumulation (Fig. S5G) and normalized ACSL4, GPX4 and FTH1 expression (Fig. S5H). Collectively, these findings demonstrate that OTUB1 suppresses autophagy-dependent ferroptosis by preferentially binding p62 to inhibit autophagic flux.

### OTUB1 drives HCC progression by stabilizing p62 via non-canonical deubiquitination to suppress autophagy-dependent ferroptosis

**Fig. 3:**
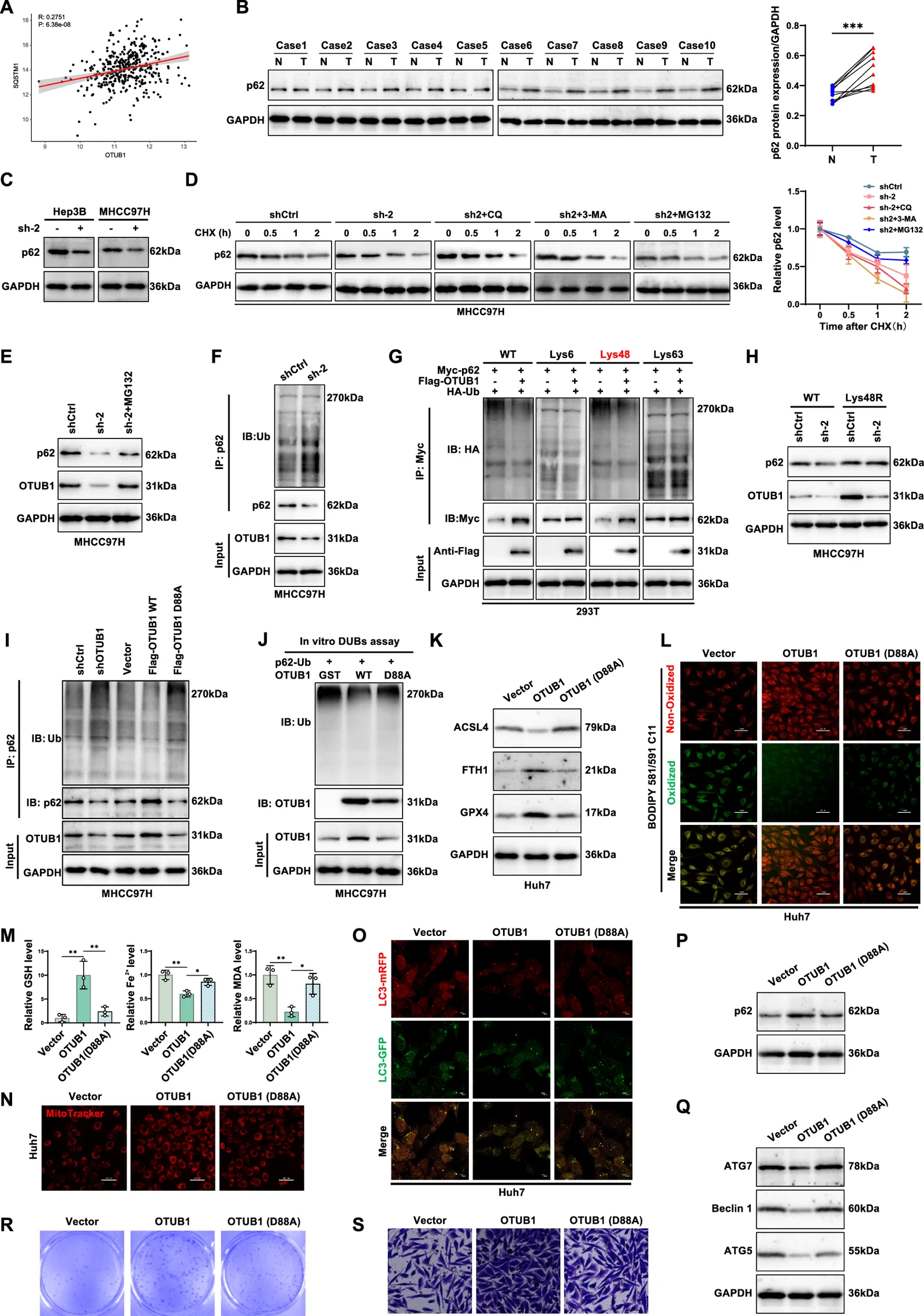
OTUB1 stabilizes p62 through non-canonical deubiquitination to inhibit autophagy-dependent ferroptosis and drive HCC progression. **A** Correlation analysis between OTUB1 and p62. **B** Western blot analysis of p62 protein in paired tumor and adjacent non-tumor tissues from 10 HCC patients. (**C**-**E**, **P**) Western blot analysis of p62 in OTUB1-knockdown Hep3B/MHCC97H cells (**C**), after CHX chase with lysosome/autophagy/proteasome inhibitors (**D**, *n* = 3), with/without MG132 treatment (**E**), and in Huh7 cells expressing Vector, OTUB1, or OTUB1 (D88A) (**P**). Ubiquitination assays: p62 ubiquitination in MHCC97H cells with OTUB1 knockdown with or without MG132 (**F**), in HEK293T cells with Myc-p62, Flag-OTUB1, and HA-Ub variants (**G**), and in cells with Ub WT or Ub Lys48R (**H**). **I** Denaturing in vivo ubiquitination assay assessing the covalent modification of p62. MHCC97H cells were subjected to shOTUB1 or transfected with Flag-OTUB1 WT versus the D88A mutant, in the presence of MG132. Immunoprecipitation of p62 was performed under denaturing conditions, followed by immunoblotting for ubiquitin. **J** In vitro ubiquitination assays in MHCC97H cells expressing WT or D88A OTUB1. Western blot of ferroptosis-related proteins (**K**) and autophagy-related proteins ATG5, ATG7, Beclin1 (**Q**) in Huh7 cells with Vector, OTUB1, or OTUB1 (D88A). **L** Confocal microscopy of lipid peroxidation by BODIPY 581/591 C11 staining in cells. Scale bar: 100 μm. **M** Analysis of GSH, MDA, and Fe^2+^ concentrations in Huh7 cells treated with Vector, OTUB1, or OTUB1 (D88A) (*n* = 3). **N** Mitochondrial membrane potential detected by MitoTracker Red. Scale bar: 100 μm. **O** Confocal microscopy of mRFP-GFP-LC3 puncta in cells (*n* = 3). Scale bar: 10 μm. **R** Colony formation assays were performed to assess the proliferation of Huh7 cells. **S** Transwell assays were performed to evaluate the migration of Huh7 cells. Scale bar, 100 μm. *, *P* < 0.05; **, *P* < 0.01; ***, *P* < 0.001.

To elucidate the molecular basis of p62 regulation, we first analyzed clinical samples and observed a positive correlation between OTUB1 and p62 expression (Fig. 3A, B). Degradation assays revealed that p62 reduction caused by OTUB1 knockdown was rescued by the proteasome inhibitor MG132, but not by autophagy inhibitors, implicating the ubiquitin-proteasome system (Fig. 3C–E). To pinpoint the specific contributions of proteasomal versus autophagic degradation, we genetically silenced the core autophagy gene ATG5 in OTUB1-knockdown cells and performed CHX chase assays. The accelerated degradation of p62 induced by OTUB1 knockdown was reversed by the proteasome inhibitor MG132, whereas ATG5 knockdown provided only marginal rescue (Fig. S6A–C). This clarifies that upon OTUB1 depletion, p62 is predominantly degraded via the ubiquitin-proteasome system. Mechanistically, OTUB1 specifically removed K48-linked polyubiquitin chains from p62 without affecting K63- or K6-linkages, a finding confirmed by the rescue effect of the K48R ubiquitin mutant (Fig. 3F–H). OTUB1 exerts deubiquitination through two distinct mechanisms: canonical catalytic activity dependent on the C91 residue and non-canonical E2-inhibitory activity dependent on the D88 residue [14, 24, 25]. To distinguish the deubiquitination mode, we utilized the OTUB1-D88A mutant (defective in non-canonical activity). Denaturing immunoprecipitation clearly demonstrated that OTUB1 depletion increased the polyubiquitination of p62 compared to the control. Conversely, overexpression of Wild-Type (WT) OTUB1 significantly reduced covalent p62 ubiquitination. Crucially, the OTUB1-D88A mutant failed to deubiquitinate p62, yielding a ubiquitin smear comparable to the control (Fig. 3I). In vitro ubiquitination experiments further confirmed this result (Fig. 3J).

To determine the functional consequences, we assessed ferroptotic and autophagic phenotypes. OTUB1-D88A failed to replicate the anti-ferroptotic effects of WT, resulting in elevated lipid ROS, Fe^2+^, mitochondrial damage, and pro-ferroptotic marker expression (Figs. 3K–N and S6D–F). Furthermore, D88A reversed OTUB1-mediated autophagy suppression (Figs. 3O–Q and S6G–I) and significantly attenuated the promotion of cell proliferation and migration (Figs. 3R, S, and S6J, K). Collectively, these findings demonstrate that OTUB1 stabilizes p62 via D88-dependent non-canonical deubiquitination, thereby inhibiting autophagy-dependent ferroptosis to drive HCC progression.

### Knockdown of p62 reverses OTUB1 overexpression-mediated malignant progression of HCC in vitro and in vivo

**Fig. 4:**
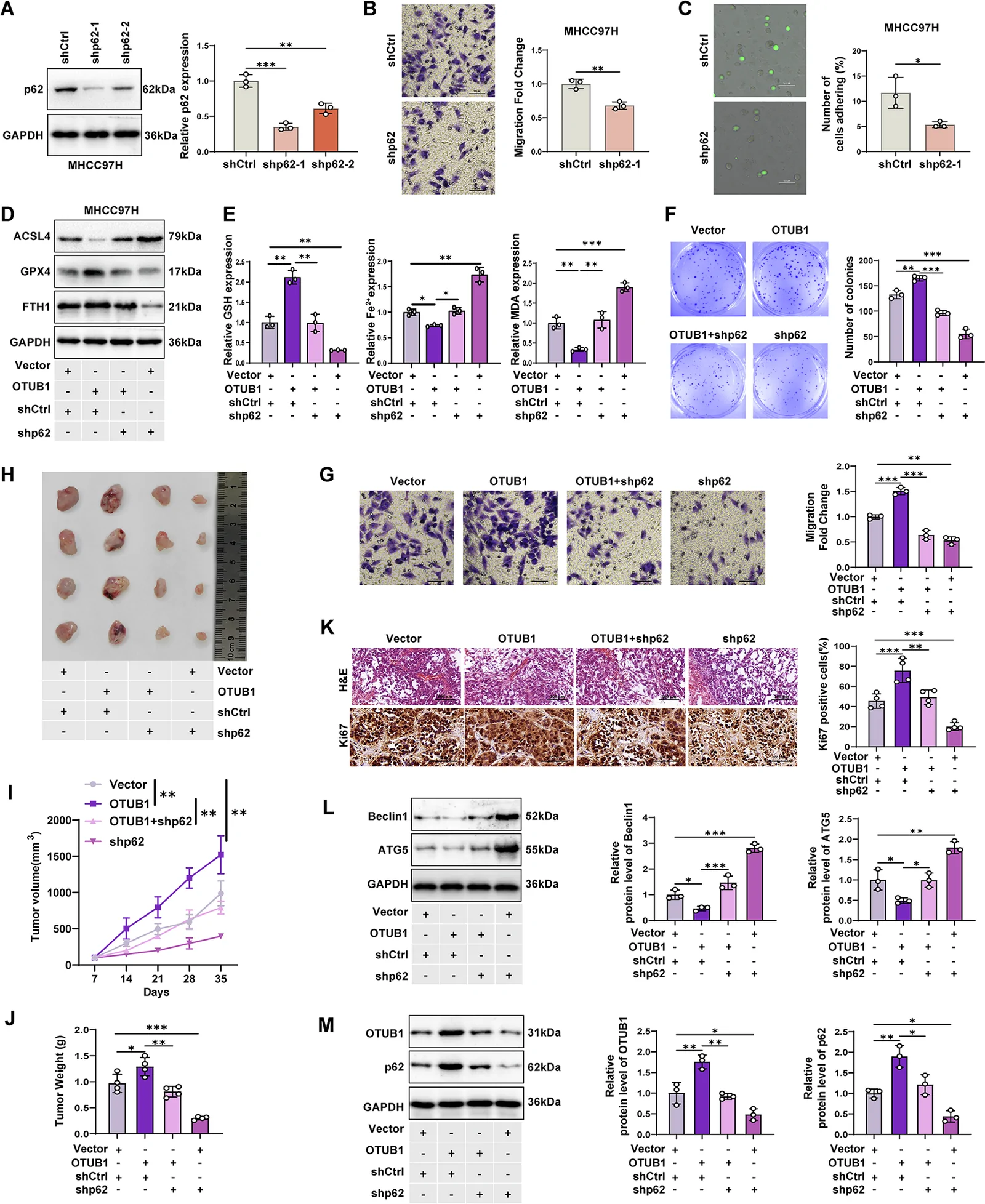
Knockdown of p62 reverses OTUB1 overexpression-mediated malignant progression of HCC in vitro and in vivo. **A** Western blot of p62 protein in p62-knockdown MHCC97H cells (*n* = 3). Transwell assays showing migration of MHCC97H cells after p62 knockdown (**B**) and in OTUB1-overexpressing cells with/without p62 knockdown (**G**) (*n* = 3). Scale bar: 100 μm. **C** Cell adhesion ability assessed by Calcein AM staining (*n* = 3). Scale bar: 100 μm. Western blot of ferroptosis-related proteins (**D**), autophagy proteins Beclin1 and ATG5 (**L**), and OTUB1/p62 (**M**) (*n* = 3). **E** GSH, MDA, and Fe^2+^ levels in indicated cells (*n* = 3). **F** Colony formation assays evaluating proliferative capacity (*n* = 3). Xenograft experiments: representative tumors (**H**), tumor volume (**I**), and tumor weight (**J**) in nude mice (*n* = 4). **K** IHC analysis of Ki67 in xenograft tumors (*n* = 4). Scale bar: 100 μm. *, *P* < 0.05; **, *P* < 0.01; ***, *P* < 0.001.

To establish p62 as the critical downstream effector of OTUB1, we first validated that p62 knockdown significantly impaired HCC cell migration and adhesion (Fig. 4A–C). In rescue experiments, p62 depletion effectively reversed the oncogenic phenotypes driven by OTUB1 overexpression, neutralizing the suppression of ferroptosis and the enhancement of cell proliferation and migration (Fig. 4D–G). To validate this regulatory axis in vivo, we utilized a xenograft model. Consistent with in vitro findings, p62 knockdown abrogated OTUB1-induced tumor growth, as evidenced by reduced tumor volume, weight, and Ki67 proliferation indices (Fig. 4H–K). Mechanistically, protein analysis of tumor tissues confirmed that p62 depletion restored autophagic flux, characterized by upregulated Beclin1 and ATG5 expression (Fig. 4L, M). Collectively, these findings demonstrate that OTUB1 promotes HCC malignant progression primarily by stabilizing p62.

### The OTUB1 inhibitor enhances the efficacy of Lenvatinib by inducing autophagy-dependent ferroptosis

**Fig. 5:**
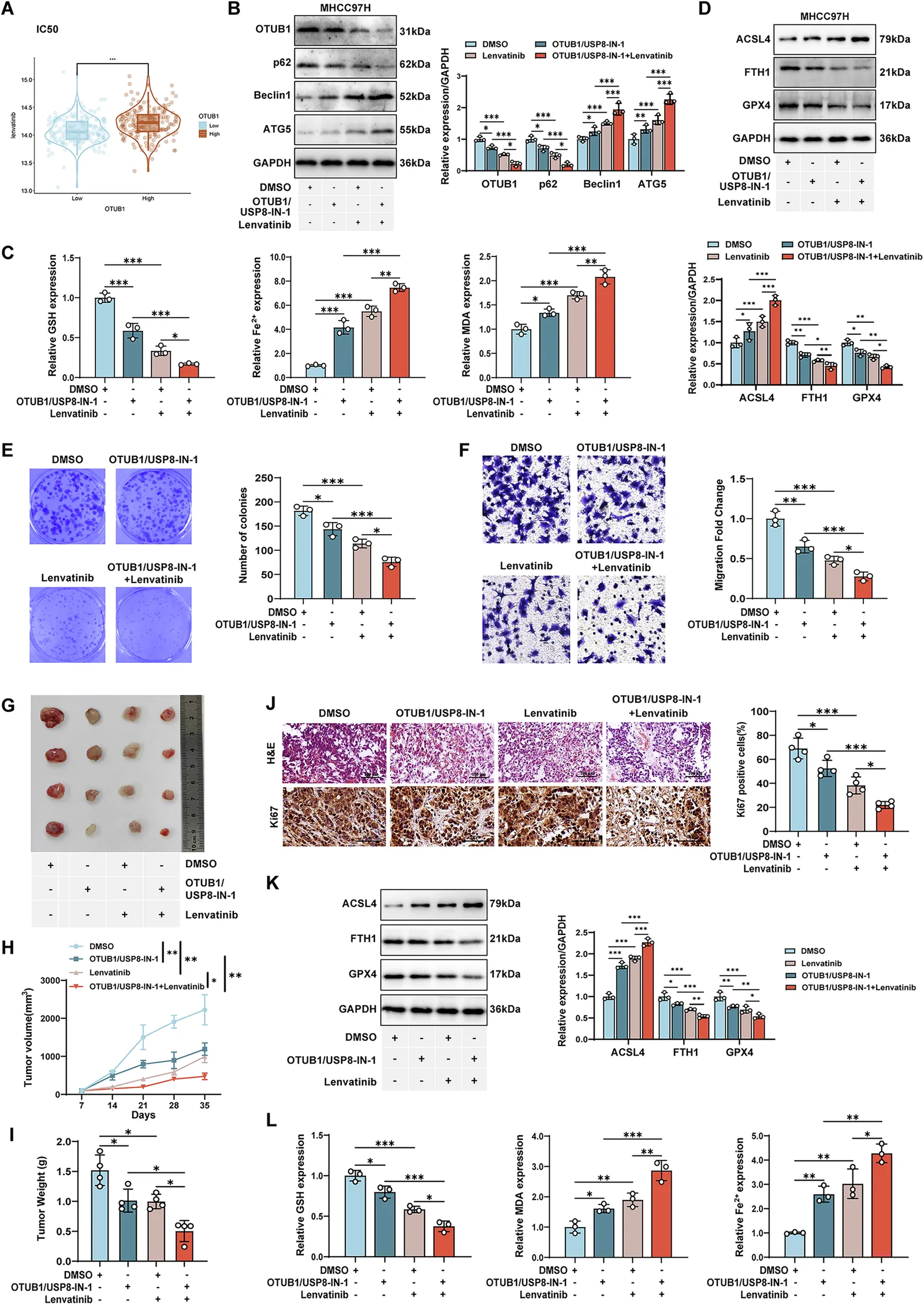
Combined treatment with OTUB1/USP8-IN-1 and Lenvatinib synergistically inhibits HCC progression in vitro and in vivo. **A** Box plots showing significantly elevated IC_50_ values of Lenvatinib in OTUB1-high cells (orange) compared to OTUB1-low cells (blue). Western blot of autophagy markers and OTUB1 in MHCC97H cells (**B**, *n* = 3), ferroptosis proteins in MHCC97H cells (**D**, *n* = 3), and ferroptosis proteins in xenograft tumors (**K**, *n* = 3). Analysis of GSH, MDA, and Fe^2+^ concentrations in MHCC97H cells (**C**) and tumor tissues (**L**) (*n* = 3). **E** Colony formation assays assessing proliferation in MHCC97H cells (*n* = 3). **F** Transwell assays evaluating migration in MHCC97H cells (*n* = 3). Scale bar: 100 μm. Xenograft experiments: representative tumors (**G**), tumor volume (**H**), and tumor weight (**I**) across treatment groups (*n* = 4). **J** IHC analysis of Ki67 expression in xenograft tumors (*n* = 4). Scale bar: 100 μm. *, *P* < 0.05; **, *P* < 0.01; ***, *P* < 0.001.

Lenvatinib, a multi-receptor tyrosine kinase inhibitor, serves as a first-line therapy for advanced HCC. However, its clinical benefit is limited by drug resistance, which has been linked to ferroptosis resistance [26]. Building on our finding that OTUB1 knockdown suppresses autophagy-dependent ferroptosis and considering the significant association between high OTUB1 expression and Lenvatinib resistance in HCC patients (Fig. 5A), we investigated whether pharmacological inhibition of OTUB1 could overcome Lenvatinib resistance and enhance its efficacy. In vitro, while monotherapy with the OTUB1 inhibitor (OTUB1/USP8-IN-1) or Lenvatinib triggered autophagy and ferroptosis, the combination treatment exhibited marked synergy, significantly amplifying these pathways and suppressing proliferation and migration (Fig. 5B–F). To validate this therapeutic strategy in vivo, we employed a xenograft model. The combination therapy demonstrated the most potent inhibition of tumor growth (Fig. 5G–I), a finding corroborated by significantly reduced Ki67 proliferation indices (Fig. 5J). Mechanistically, tissue analysis confirmed that the combination regimen synergistically enhanced the activation of ferroptosis markers beyond levels achieved by single agents (Fig. 5K, L). Collectively, these results demonstrate that pharmacological inhibition of OTUB1 enhances Lenvatinib efficacy by inducing autophagy-dependent ferroptosis.

## Discussion

In this study, we address the critical challenge of targeting HCC by exploiting its vulnerability to ferroptosis, an iron-dependent, lipid peroxidation-driven form of regulated cell death [27]. Consequently, targeting the ferroptosis regulatory network represents a highly promising anti-cancer strategy [28]. While ferroptosis induction represents a promising therapeutic avenue, the specific regulatory networks governing ferroptosis resistance in HCC remain incompletely understood. Our investigation established that OTUB1 is significantly upregulated in HCC tissues and serves as a potent driver of malignant progression. The core finding of this work is that OTUB1 promotes HCC cell proliferation, migration, and EMT specifically by suppressing autophagy-dependent ferroptosis. We demonstrated that OTUB1 depletion reactivates this cell death pathway, leading to the accumulation of lipid ROS and iron, mitochondrial dysfunction, and tumor suppression. Mechanistically, we identified that OTUB1 stabilizes the autophagy receptor p62 (SQSTM1) via a non-canonical deubiquitinating mechanism. This stabilization prevents the autophagic degradation of p62, which in turn inhibits autophagic flux. The suppression of autophagy subsequently blocks the degradation of ferroptosis inhibitors (such as GPX4 and FTH1) and prevents the accumulation of ferroptosis drivers (such as ACSL4), ultimately conferring ferroptosis resistance to HCC cells.

Our findings align with and expand upon the emerging understanding of deubiquitinating enzymes (DUBs) as critical modulators of ferroptosis [29]. Previous studies have implicated OTUB1 as a negative regulator of ferroptosis in other malignancies. For instance, OTUB1 has been reported to stabilize GPX4 protein, thereby inhibiting ferroptosis and promoting gastric cancer metastasis [18]. It also modulates SLC7A11 stability, mediating CD44v-dependent ferroptosis resistance in malignancies [30]. Furthermore, the FTO-OTUB1 axis has been shown to confer chemoresistance in nasopharyngeal carcinoma by inhibiting ferroptosis [19]. Consistent with these reports, our data confirms OTUB1’s role as a pro-tumorigenic factor associated with adverse clinicopathological features and poor prognosis [31]. Furthermore, our study reinforces the concept of ferroptosis as an “autophagy-dependent” process. Existing literature indicates that selective forms of autophagy-such as ferritinophagy and lipophagy-are essential for degrading iron-storage proteins and generating lipid peroxidation substrates to execute ferroptosis [22, 32, 33]. Our rescue experiments, using autophagy inhibitors and p62 overexpression, provide robust causal evidence supporting this consensus, demonstrating that OTUB1-mediated ferroptosis suppression is strictly dependent on its ability to impair autophagic flux.

Unlike previous studies that primarily focused on the catalytic stability of substrates, our research introduces a novel structural and mechanistic dimension to OTUB1 biology in HCC. First, we employed an innovative approach combining Co-IP/Mass Spectrometry with AlphaFold3 structural prediction. This allowed us to characterize the competitive nature of OTUB1 binding. We discovered that OTUB1 binds with high affinity to the autophagy receptor p62, competitively displacing the autophagy initiator Beclin1. These factors play opposing roles: p62 facilitates autophagosomal clearance, whereas Beclin1 nucleates the autophagic machinery [34]. AlphaFold3 uniquely predicted that both proteins compete for the same GLU214 residue on OTUB1, providing a structural basis for why OTUB1 overexpression acts as a molecular switch to suppress autophagic initiation.

Second, we elucidated the specific mode of ubiquitin regulation. While p62 stability is known to be regulated by E3 ligases like TRAF2 [35] or stabilizing factors like SERPINH1 [36], our study is the first to demonstrate that OTUB1 stabilizes p62 via its non-canonical, D88-dependent activity [24, 37]. By utilizing the OTUB1-D88A mutant (which disrupts E2 enzyme sequestration) versus the OTUB1-C91A mutant, we proved that OTUB1 prevents p62 degradation not merely by catalytically cleaving ubiquitin chains, but by blocking the transfer of ubiquitin from E2 enzymes to p62. Specifically, OTUB1 inhibits the formation of K48-linked polyubiquitin chains on p62, thereby shielding it from the proteasome. This precise dissection of the non-canonical mechanism distinguishes our work from general descriptions of DUB activity in cancer.

The most significant translational insight from our study lies in overcoming drug resistance. Lenvatinib is a first-line therapy for advanced HCC, but its efficacy is severely limited by primary or acquired resistance [3, 38]. Our correlation analysis revealed a strong link between high OTUB1 expression and poor response to Lenvatinib. We demonstrate that while Lenvatinib alone induces some degree of ferroptosis [39–41], OTUB1 acts as a brake on this process. Strikingly, the combination of an OTUB1 inhibitor (OTUB1/USP8-IN-1) with Lenvatinib produced a synergistic anti-tumor effect in vivo, significantly amplifying autophagy-dependent ferroptosis. This suggests that targeting OTUB1 represents a viable strategy to sensitize refractory HCC tumors to kinase inhibitor therapy.

Despite these promising findings, several limitations warrant future investigation. First, while we confirmed the role of the D88 residue, the specific E2 conjugating enzyme(s) sequestered by OTUB1 to prevent p62 ubiquitination remain to be identified. Second, current pharmacological inhibitors of OTUB1 may have off-target effects; the development of more specific, high-affinity OTUB1 antagonists is necessary for clinical translation. Finally, future studies utilizing OTUB1-C91A mutants are needed to fully rule out any minor contributions of canonical catalytic activity to this phenotype. Nevertheless, OTUB1 stands out as a valuable biomarker for prognosis and a potent therapeutic target.

In summary, our study identifies the OTUB1-p62 axis as a critical regulator of HCC progression that functions by suppressing autophagy-dependent ferroptosis. Beyond elucidating the non-canonical mechanism underlying p62 stabilization, we provide a definitive mechanistic rationale for combinatorial pharmacological intervention. Specifically, targeted inhibition of OTUB1 promotes the proteasomal degradation of p62, which subsequently relieves the competitive suppression of Beclin 1 and restores autophagic flux. This autophagic reactivation alters the cellular metabolic state, serving as a potent sensitizer to Lenvatinib-induced ferroptosis. The synergistic anti-tumor efficacy achieved by this combination strategy in vitro and in vivo highlights its translational viability. Ultimately, these findings position OTUB1 inhibition as a compelling therapeutic strategy to enhance the efficacy of Lenvatinib and improve clinical outcomes in patients with advanced HCC.

## Materials and methods

### Tissue samples and cell culture

Human HCC tissues and matched non-tumorous liver samples were obtained from The First Hospital of Jilin University with ethical approval and patient informed consent. Tissues were snap-frozen in liquid nitrogen for storage. Human HCC cell lines (HepG2, Hep3B, Huh7, MHCC97H), LX-2, and HEK293T cells (ATCC) were cultured in DMEM/high-glucose medium (HyClone, Logan City, USA) containing 10% fetal bovine serum (Gibco) under standardized culture parameters: 37 °C, 5% CO_2_, and controlled humidity.

### RNA interference, RNA isolation, and real-time PCR

Lentiviral shRNAs targeting OTUB1 or p62 (Genechem) were used for knockdown. Total RNA was extracted using TRIzol (Invitrogen), reverse-transcribed (HiScript II RT SuperMix, Vazyme), and analyzed by qPCR (SYBR Master Mix, Vazyme) on a Bio-Rad CFX96 system. The primer sequences used for qPCR were as follows: OTUB1: Forward 5’-GCTGGATGACAGCAAGGAGTTG-3’, Reverse 5’- CTTCTCCACCTGCTCAATCAGG-3’. SQSTM1 (p62): Forward 5’-TGTGTAGCGTCTGCGAGGGAAA-3’, Reverse 5’-AGTGTCCGTGTTTCACCTTCCG-3’. ATG5: Forward 5’- GCAGATGGACAGTTGCACACAC-3’, Reverse 5’- GAGGTGTTTCCAACATTGGCTCA-3’. BECN1: Forward 5’- CTGGACACTCAGCTCAACGTCA-3’, Reverse 5’- CTCTAGTGCCAGCTCCTTTAGC-3’. GAPDH: Forward 5’- GTCTCCTCTGACTTCAACAGCG-3’, Reverse 5’- ACCACCCTGTTGCTGTAGCCAA-3’. The relative mRNA expression was normalized to GAPDH and calculated using the 2^-ΔΔCt^ method.

### Plasmid construction and cell transfections

Flag-tagged OTUB1 (wild-type and D88A mutant), Myc-p62 (Sino Biological), and HA-ubiquitin plasmids (Genechem) were verified by sequencing and transfected using JetPRIME reagent (Polyplus).

### In vivo, in vitro, and denaturing ubiquitination assay

For in vivo ubiquitination, cells transfected with HA-ubiquitin ± other plasmids were treated with MG132, lysed, and subjected to co-IP with anti-HA blotting. For in vitro assays, lysates from transfected cells were immunoprecipitated with anti-Myc or anti-FLAG beads and immunoblotted. To assess covalent p62 ubiquitination and exclude non-specific interacting proteins, denaturing immunoprecipitation was performed. Briefly, cell pellets were lysed in an SDS-containing buffer (1% SDS, 50 mM Tris-HCl, pH 7.5, 0.5 mM EDTA, and 1 mM DTT) and boiled for 10 min at 95 °C. The lysates were then diluted 10-fold with standard non-denaturing lysis buffer to quench the SDS before overnight incubation with primary antibodies and Protein A/G magnetic beads.

### Transmission electron microscopy

Cell pellets were fixed in 2.5% glutaraldehyde and 1% OsO_4_, dehydrated, embedded, sectioned, and stained with uranyl acetate and lead citrate. Images were acquired using a Hitachi HT7800 TEM.

### Double-labeled adenovirus mRFP-GFP-LC3 transfection

Cells transfected with mRFP-GFP-LC3 adenovirus (Hanbio) were fixed, DAPI-stained, and imaged by confocal microscopy (Zeiss LSM 980). To quantify autophagic flux, yellow puncta (GFP^+^/mRFP^+^, representing autophagosomes) and red puncta (GFP^-^/mRFP^+^, representing autolysosomes wherein GFP fluorescence is quenched by acidic pH) were independently counted. Puncta were quantified per cell across at least 30 individual cells per condition from three independent experiments using ImageJ software.

### Co-IP and LC-MS/MS

Cell lysates in IP buffer were precleared, incubated with antibody-bound beads overnight, washed, and eluted. Samples were separated by SDS-PAGE, and gel bands were analyzed by LC-MS/MS (Biotree).

### Mouse tumor models

Five-week-old female BALB/c nude mice underwent subcutaneous implantation of 1 × 10^7^ MHCC97H cells (100 µL PBS). Animals, maintained under specific pathogen-free conditions at the Laboratory Animal Center, the First Hospital of Jilin University, with ad libitum access to chow and water, were monitored for tumor growth (caliper measurements every 7 days; volume = length × width2/2, with maximum dimension ≤ 2cm). At 100 mm^3^ tumor volume, mice were randomized into four cohorts (*n* = 4/group): DMSO vehicle control; Lenvatinib monotherapy (4 mg/kg, oral gavage, q2d); OTUB1/USP8-IN-1 monotherapy (5 mg/kg, i.p., q2d); and combination therapy. Treatments continued for 14 days. Terminal procedures at day 35 included cervical dislocation, tumor excision, and 4% PFA fixation. All protocols complied with institutional ethics regulations.

### Statistical analysis

Data are presented as mean ± SD. Categorical comparisons used Mann-Whitney U tests with Benjamini-Hochberg FDR correction. For continuous variables, two-tailed unpaired Student’s *t* tests or one-way ANOVA with Tukey’s post-hoc test were used. IC_50_ values were derived via nonlinear regression. Correlations and survival were analyzed using Pearson’s coefficient and Kaplan-Meier/log-rank tests, respectively. Significance was defined as FDR-adjusted *q* < 0.05 or *p* < 0.05. Analyses used SPSS 23.0 and GraphPad Prism 9.0.2.

Additional methods are described in Supplementary Methods.

## Supplementary information

Supplementary Materials

Western Blots

## Data Availability

The mass spectrometry proteomics data have been deposited in the PRIDE repository with the dataset identifier PXD078730 and will be made publicly available upon publication. Other data analyzed during this study are available from the corresponding author upon reasonable request.
