# Supplementary material for: OTUB1 inhibits autophagy-dependent ferroptosis in hepatocellular carcinoma by stabilizing p62 via non-canonical deubiquitination
Source: Cell Death Discov. 2026 Jul 22;12:382. doi: 10.1038/s41420-026-03241-5 (PMC13601636; doi:10.1038/s41420-026-03241-5)
Supplement: Supplementary file 1 [file 41420_2026_3241_MOESM2_ESM.pdf]

Figure1 D Case1-5 GAPDH

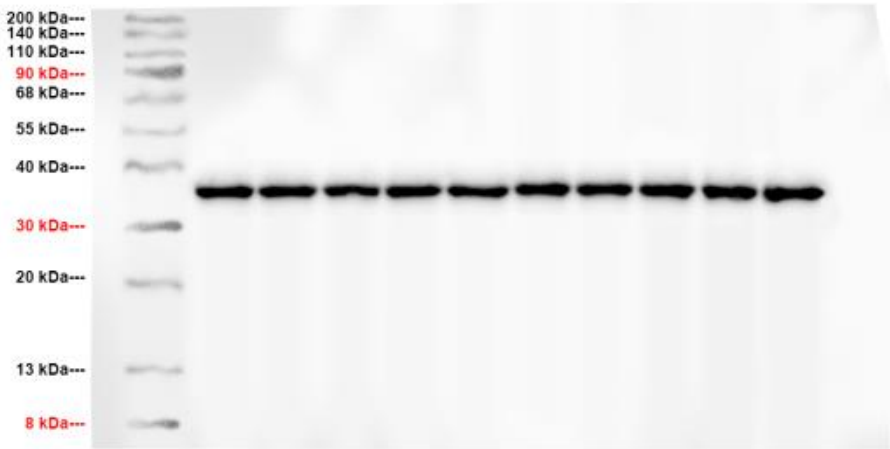

Figure1 D Case 1-5 OTUB1

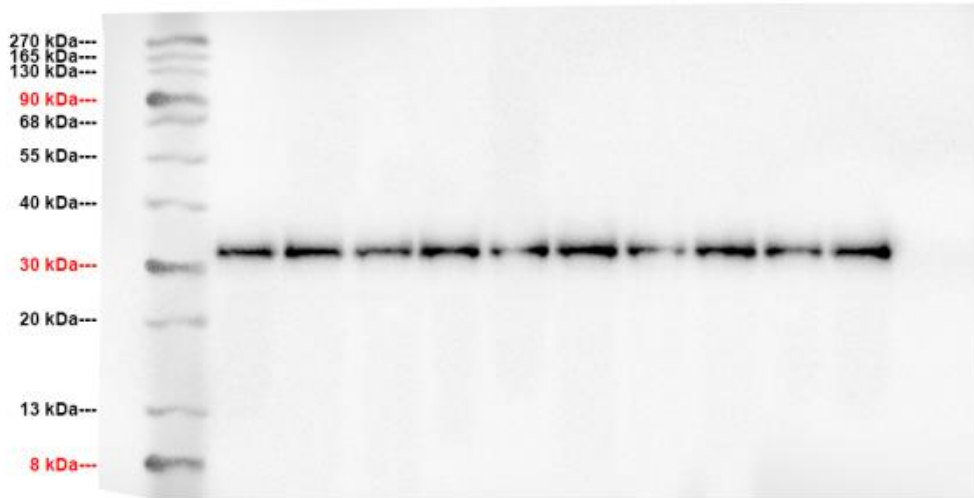

Figure1 D Case6-10 GAPDH

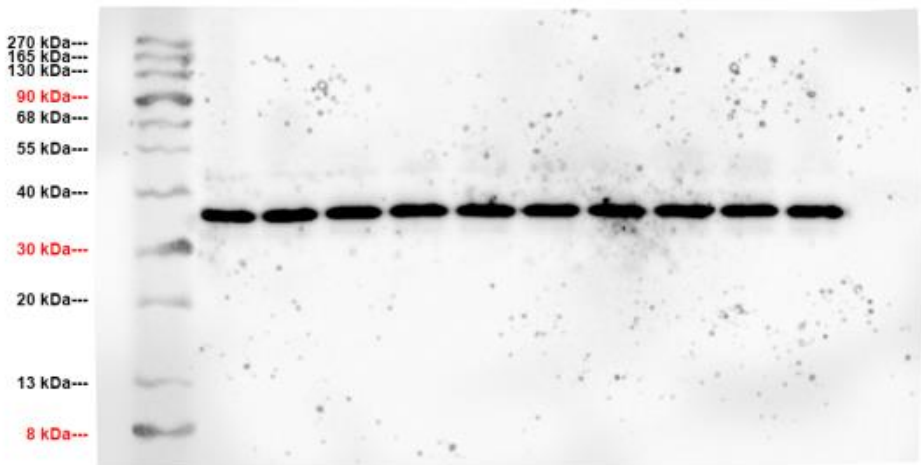

Figure1 D Case6-10 OTU8

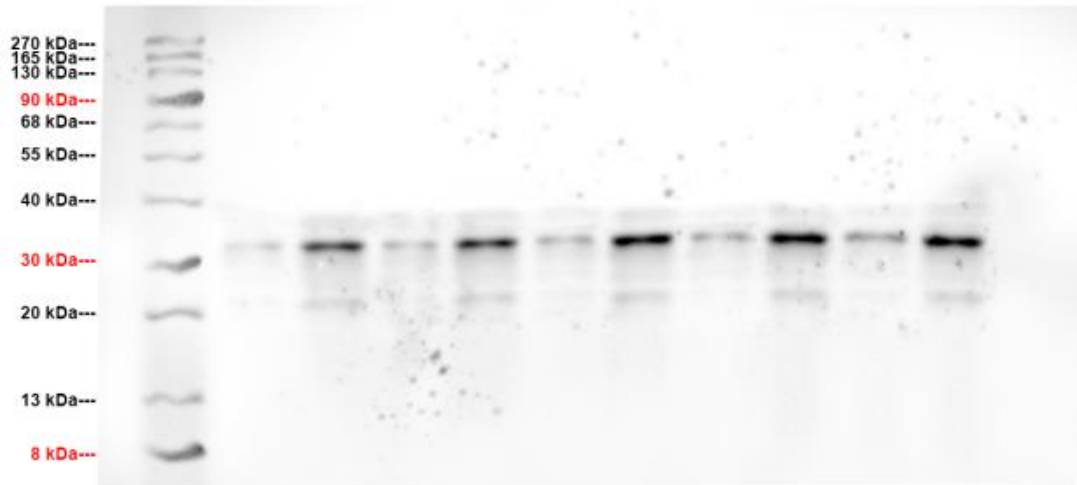

Figure1 H GAPDH

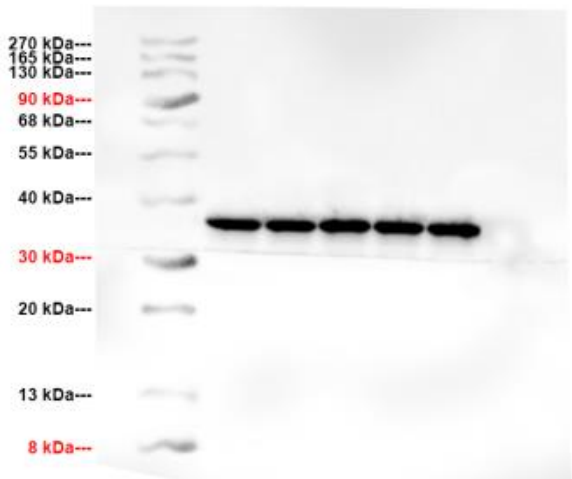

Figure1 H OTUB1

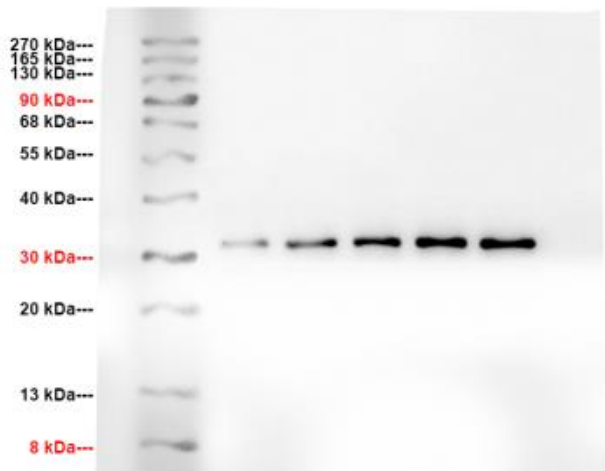

Figure1 I Hep38 GAPDH

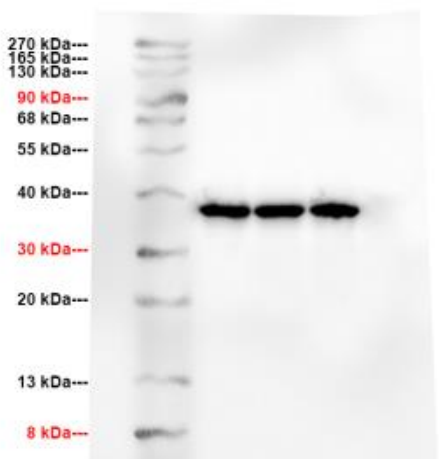

Figure1 I Hep38 OTUB1

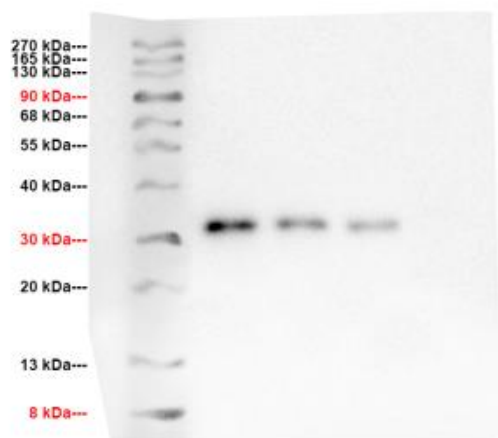

Figure1 J HepG2 GAPDH

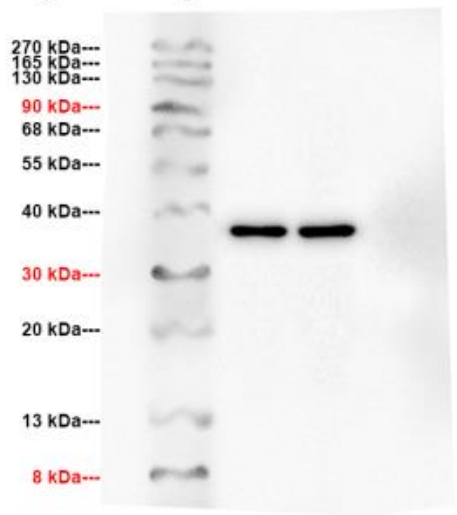

Figure1 J HepG2 OTUB1

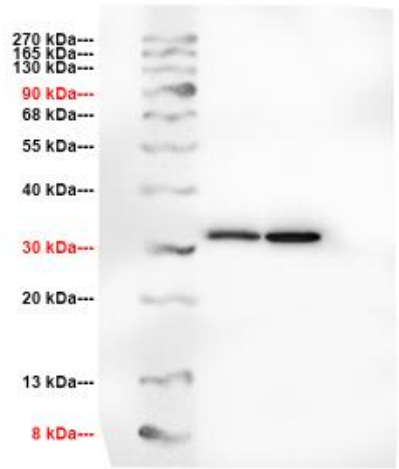

Figure2 C MHCC97H Bax

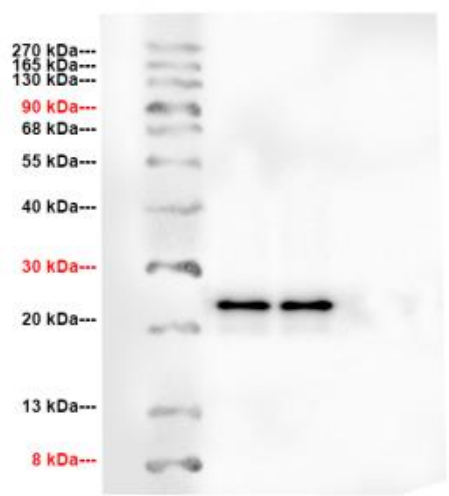

Figure2 C MHCC97H Bcl-2

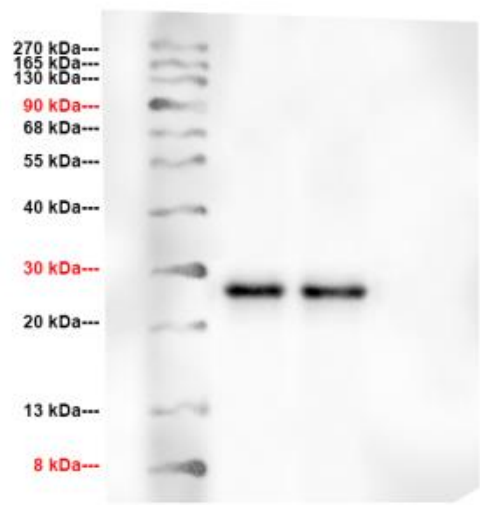

Figure2 C MHCC97H Cleaved-caspase-3

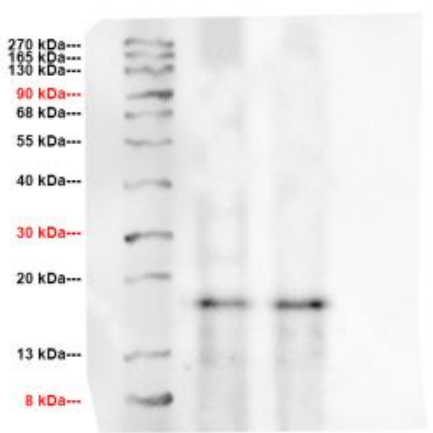

Figure2 C MHCC97H GAPDH

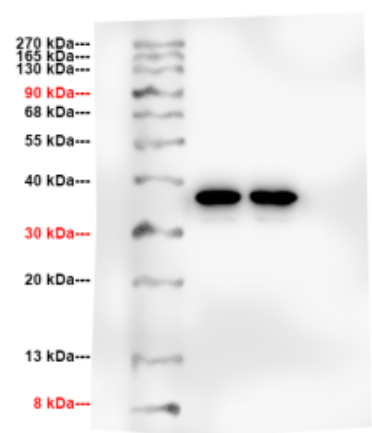

Figure2 C Hep3B Bax

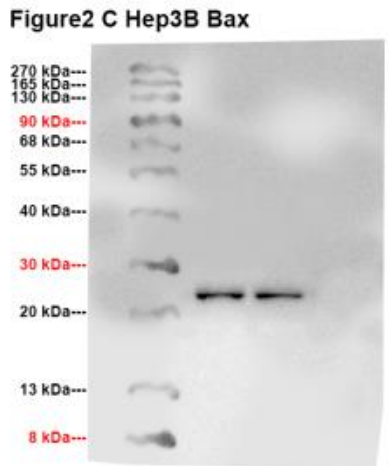

Figure2 C Hep3B Bcl-2

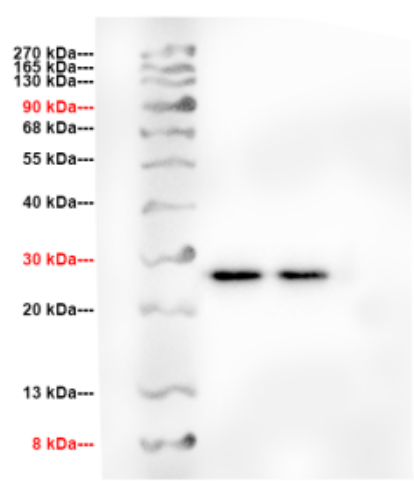

Figure2 C Hep3B Cleaved-caspase-3

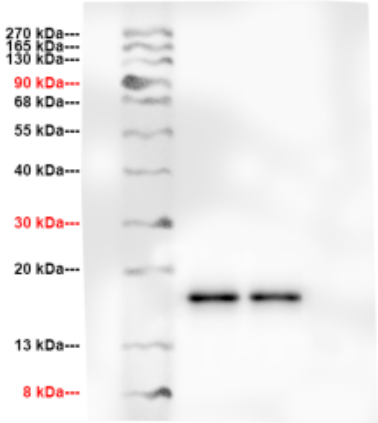

Figure2 C Hep38 GAPDH

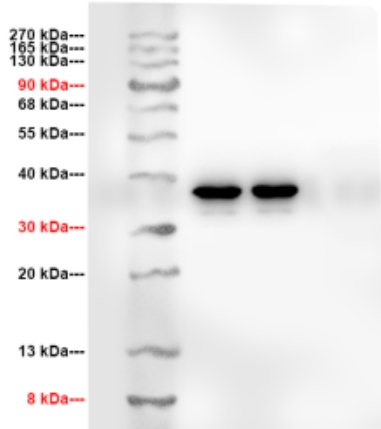

Figure1 J Huh7 GAPDH

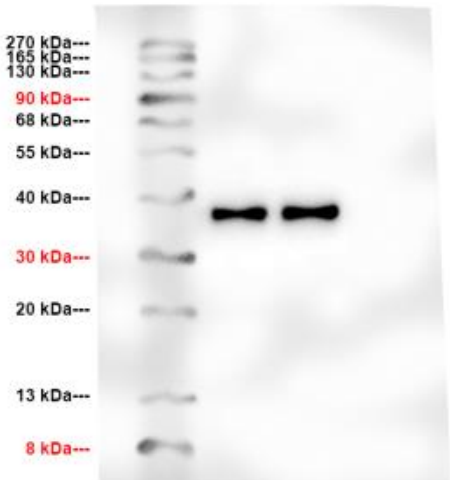

Figure1 J Huh7 OTUB1

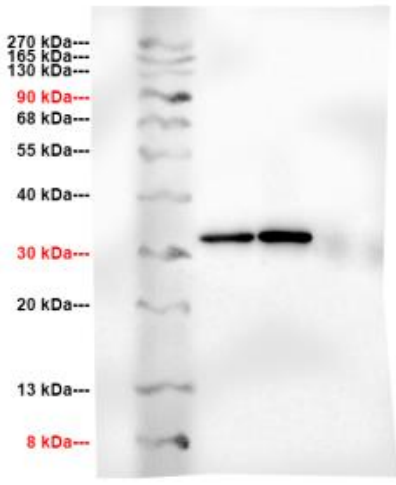

Figure2 F Hep3B ACSL4

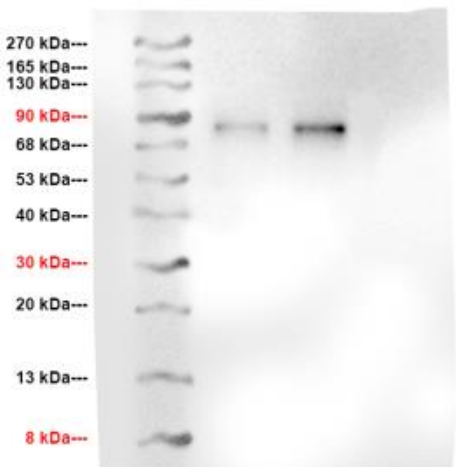

Figure2 F Hep3B FTH1

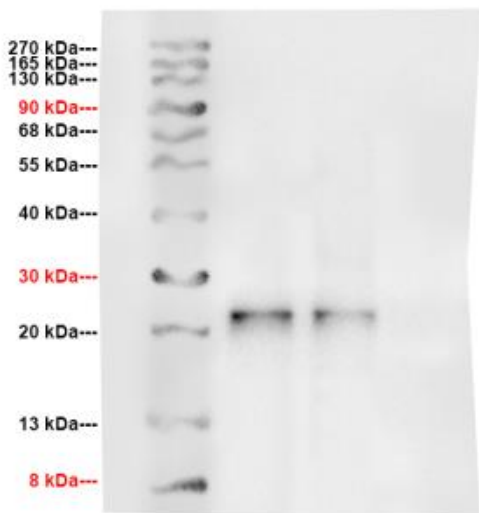

Figure2 F Hep3B GAPDH

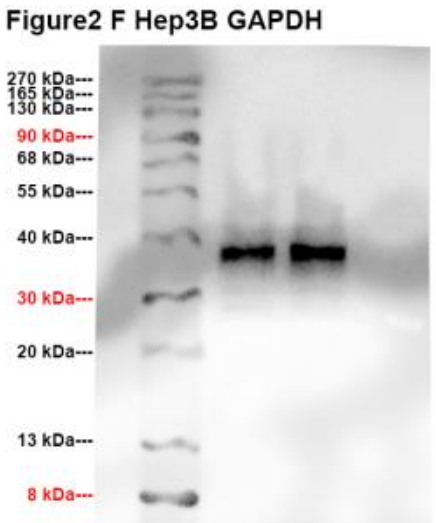

Figure2 F Hep3B GPX4

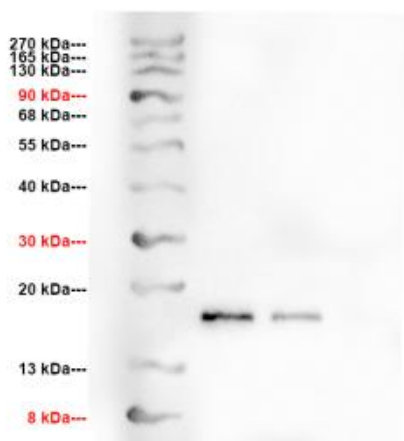

Figure1 I MHCC97H GAPDH

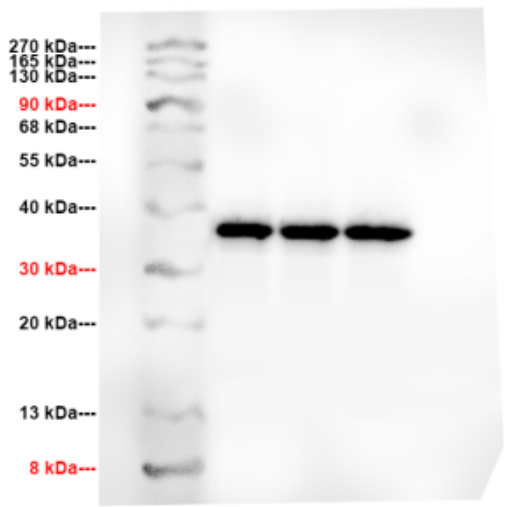

Figure1 I MHCC97H OTUB1

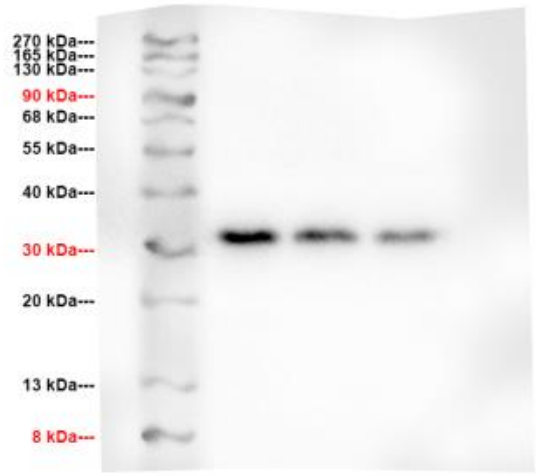

Figure2 F MHCC97H ACSL4

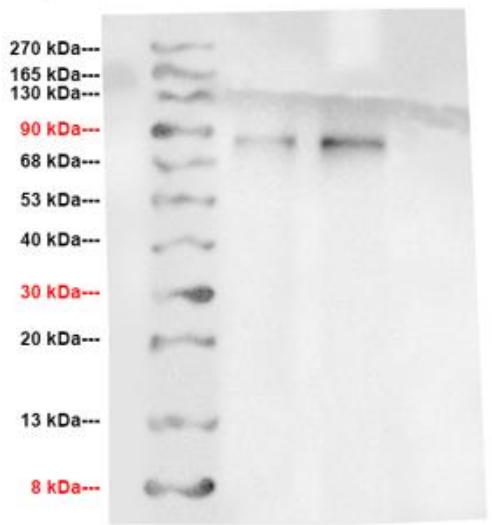

Figure2 F MHCC97H FTH1

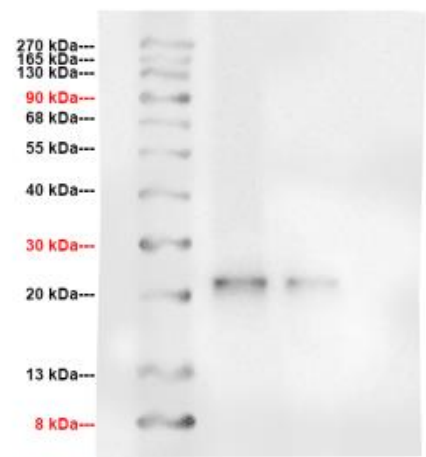

Figure2 F MHCC97H GAPDH

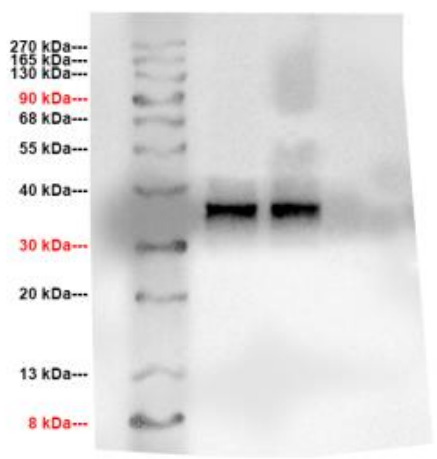

Figure2 F MHCC97H GPX4

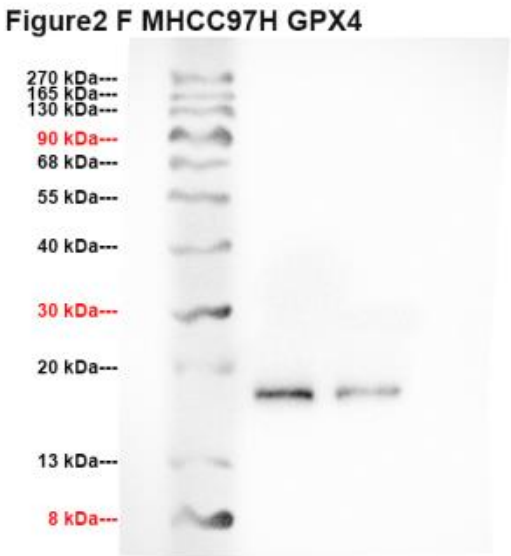

Figure2 I ACSL4

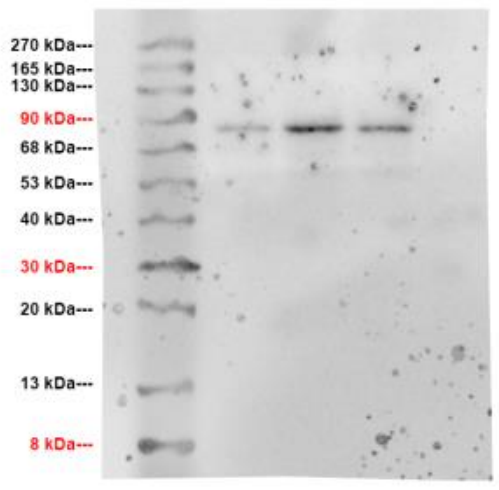

Figure2 I FTH1

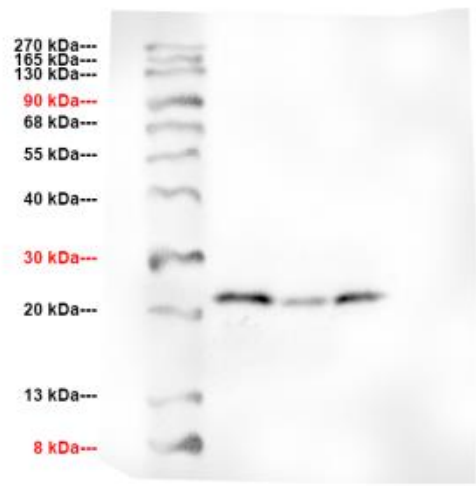

Figure2 I GAPDH

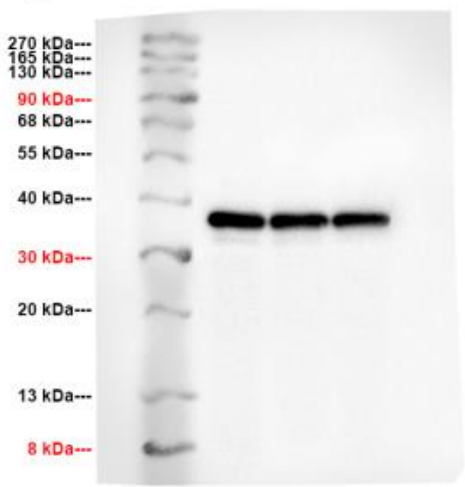

Figure2 I GPX4

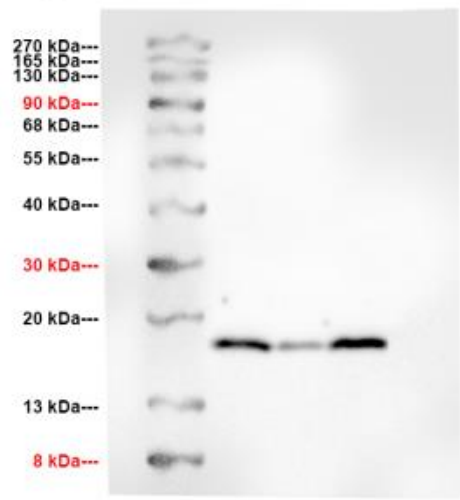

Figure3 G Beclin1 Beclin1

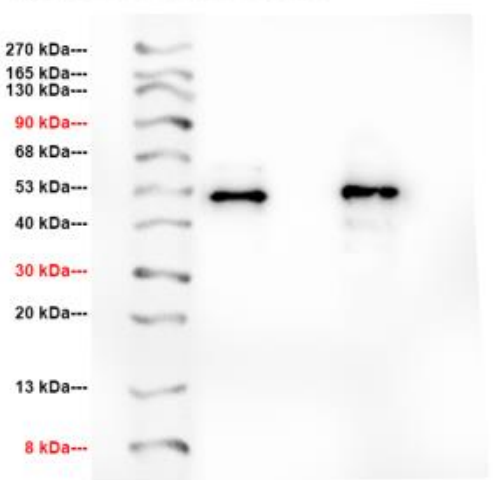

Figure3 G Beclin1 OTUB1

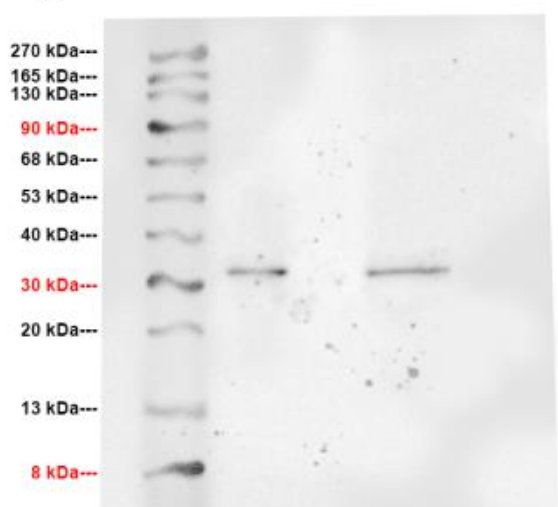

Figure3 G OTUB1 Beclin1

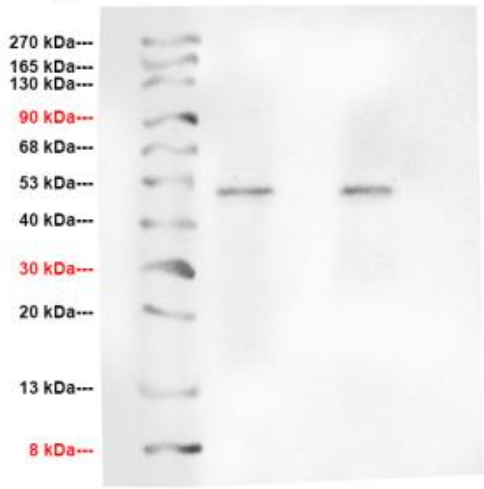

Figure3 G OYUB1 OTUB1

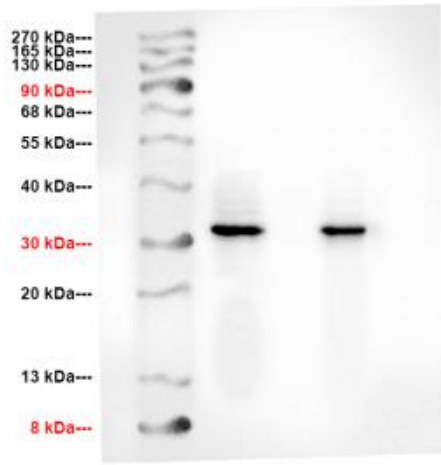

Figure3 H IP Beclin1

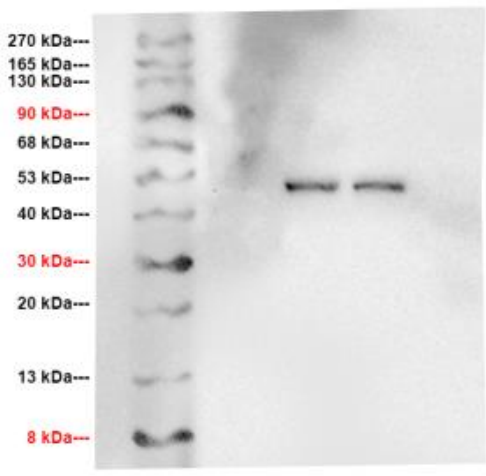

Figure3 H IP OYUB1

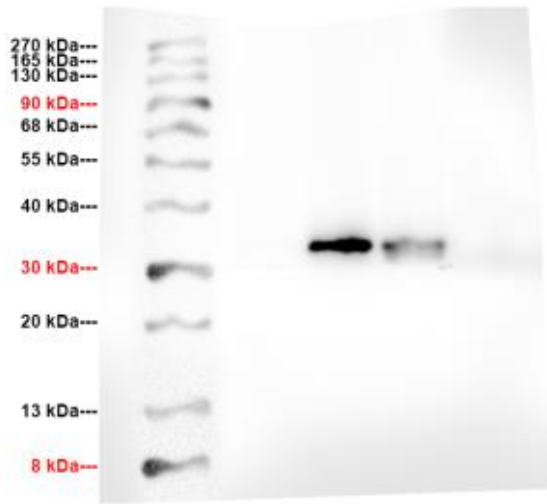

Figure3 H IP Beclin1

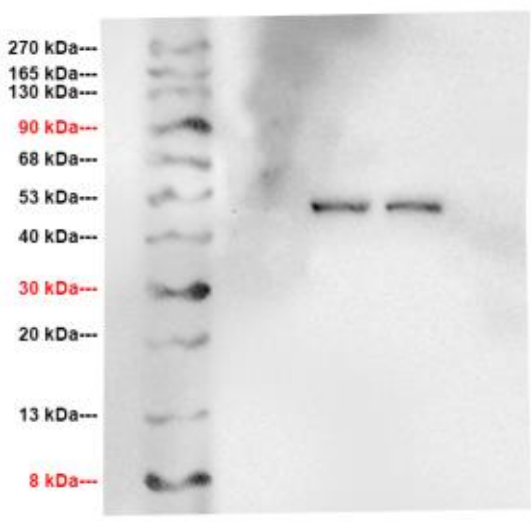

Figure3 H IP OYUB1

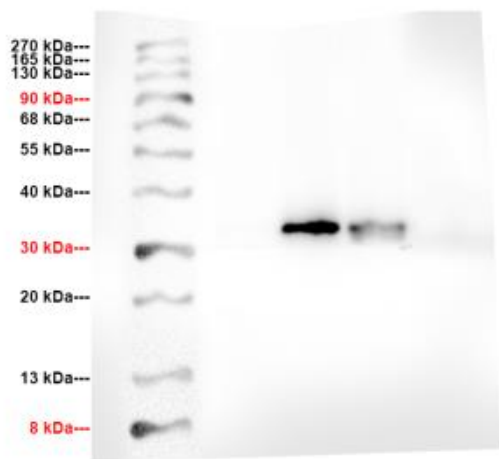

Figure3 H Input Beclin1

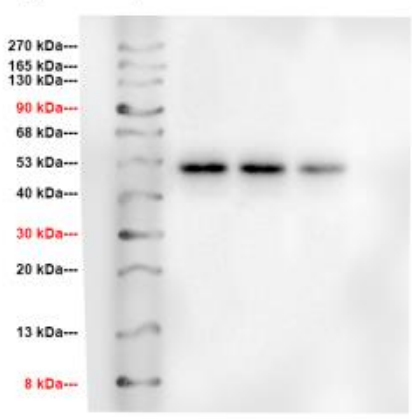

Figure3 H Input GAPDH

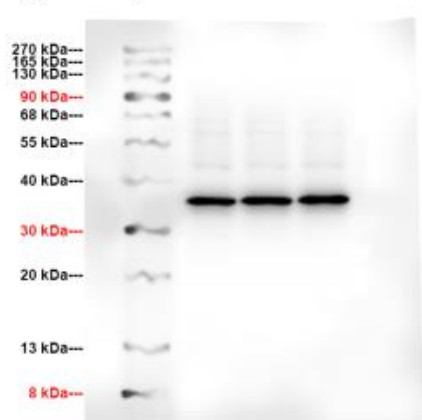

Figure3 H Imput OYUB1

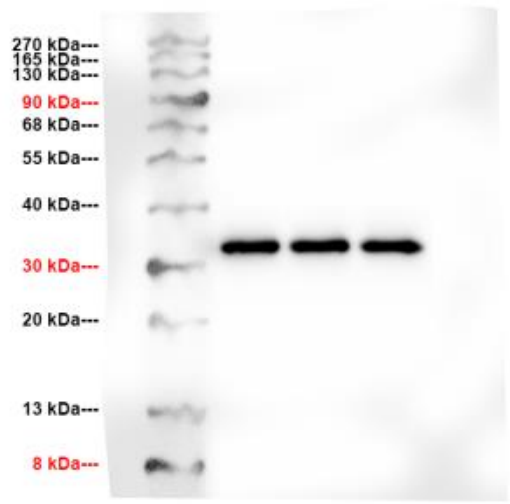

Figure3 H Input p62

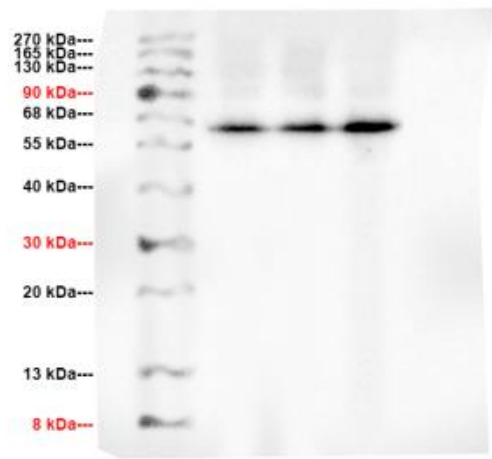

Figure3 I Input p62

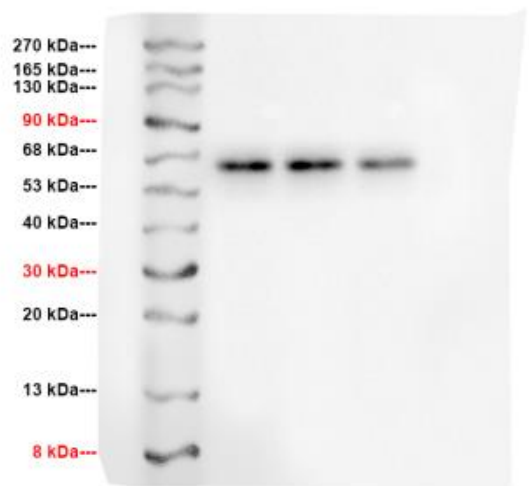

Figure3 I Input OTUB1

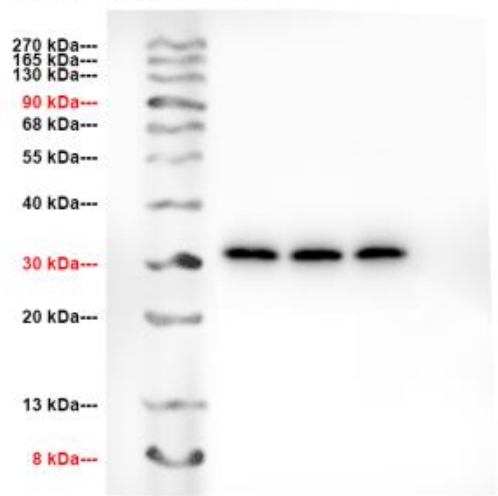

Figure3 I Input GAPDH

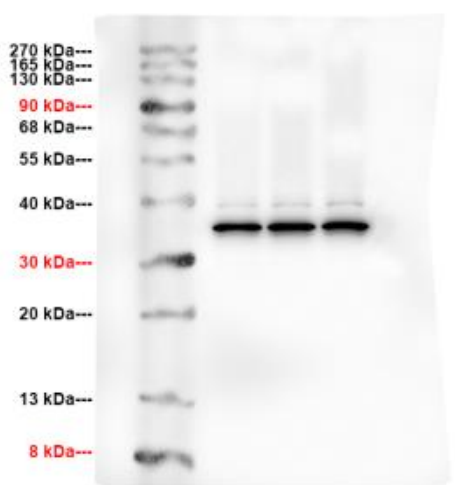

Figure3 I Input Beclin1

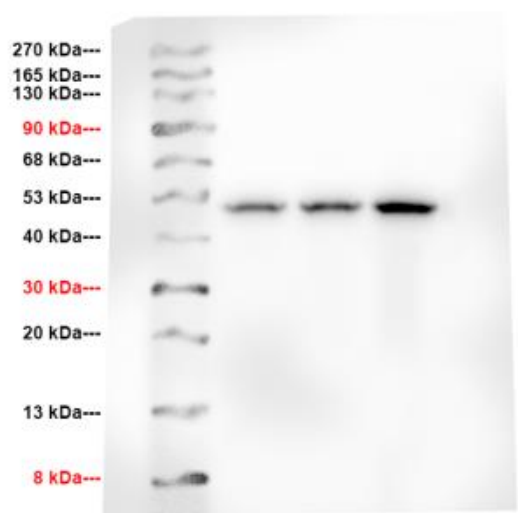

Figure3 I IP p62

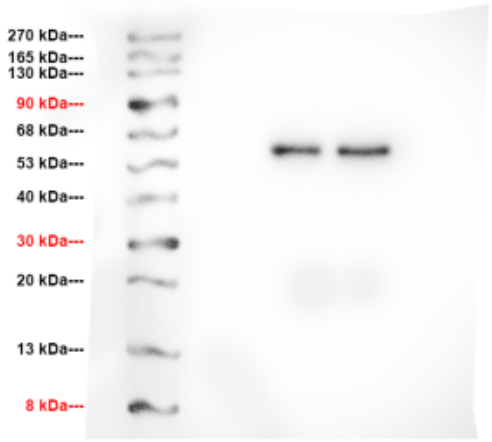

Figure3 I IP OTUB1

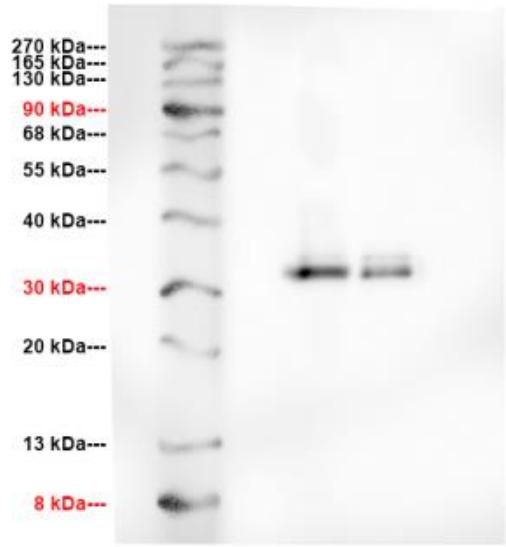

Figure3 J IB:Myc

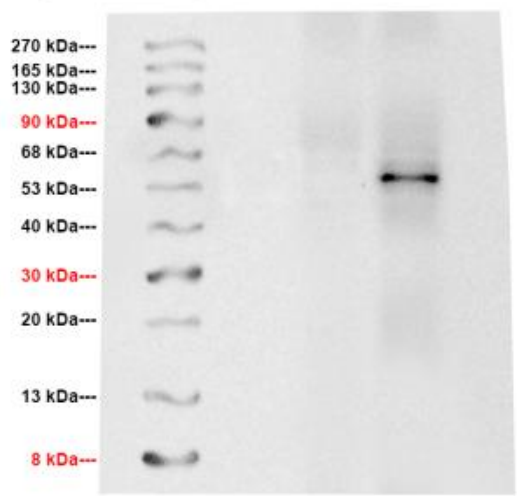

Figure3 J IB Flag

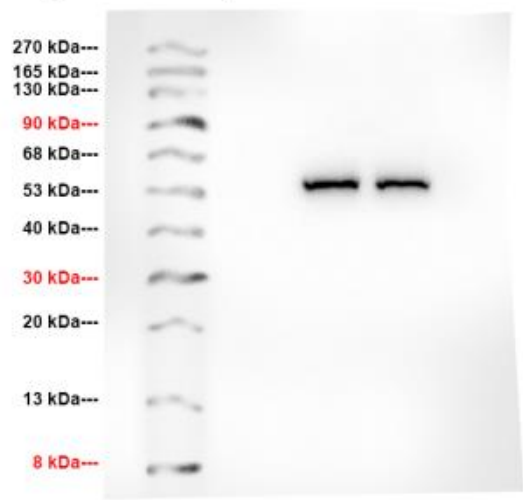

Figure3 J Anti-Myc

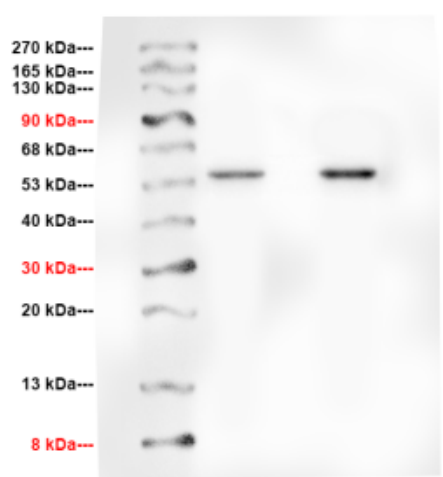

Figure3 J Anti-Flag

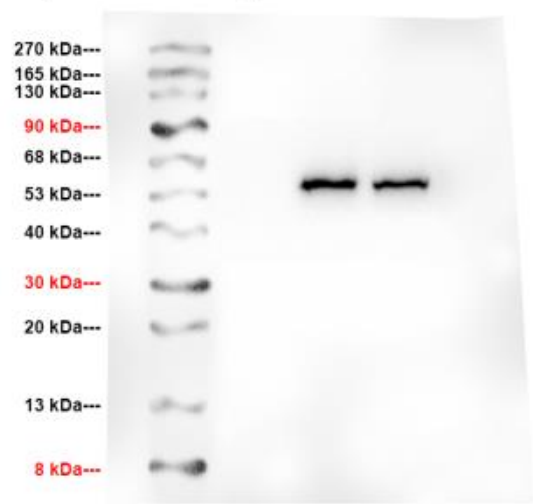

Figure3 M Hep38 p62

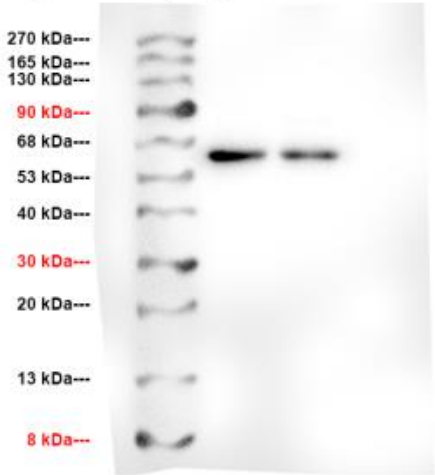

Figure3 M Hep38 GAPDH

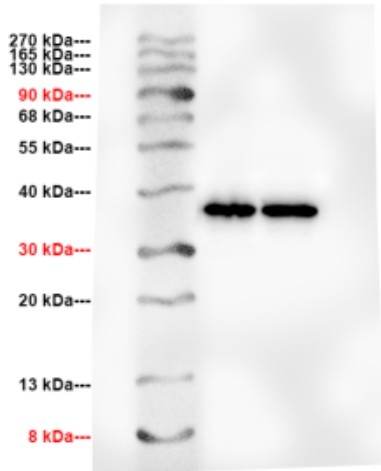

Figure3 M Hep38 Beclin1

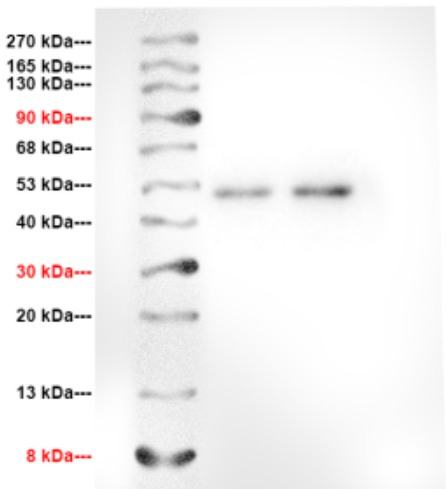

Figure3 M Hep38 ATG5

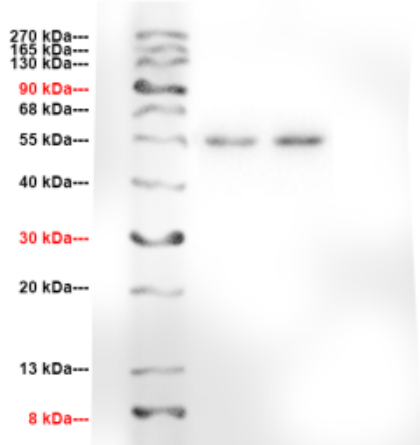

Figure3 M MHCC97H p62

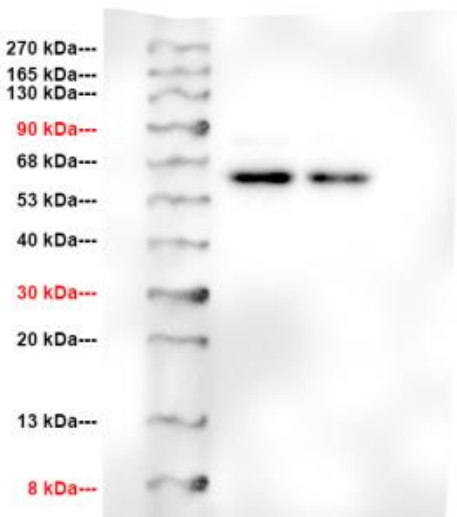

Figure3 M MHCC97H GAPDH

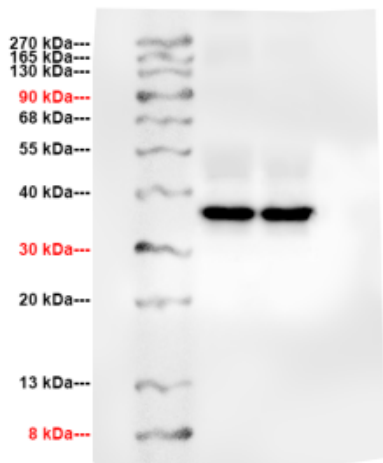

Figure3 M MHCC97H Beclin1

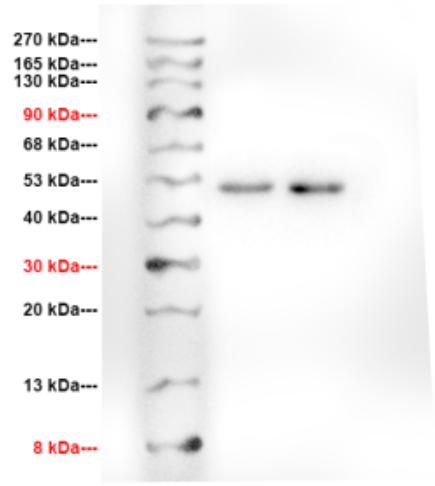

Figure3 M MHCC97H ATG5

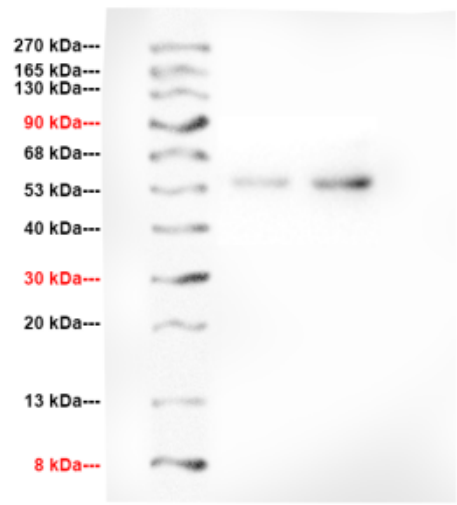

Figure4 A p62

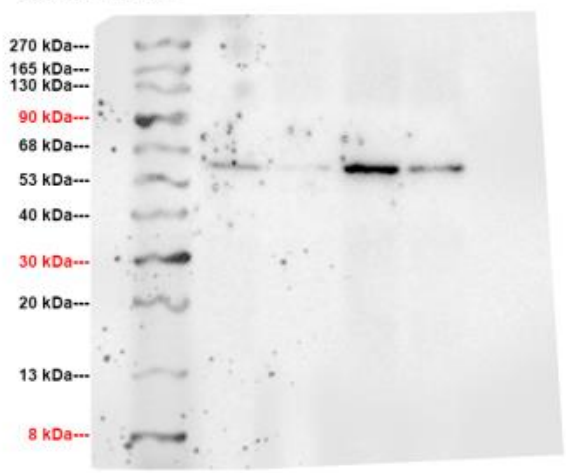

Figure4 A LC3

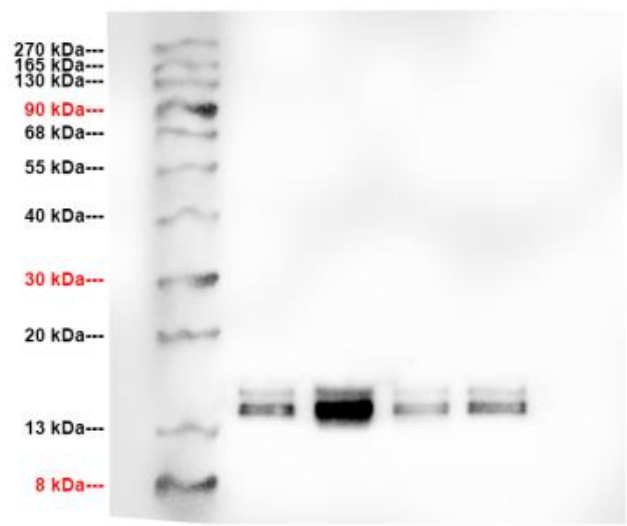

Figure4 A GAPDH

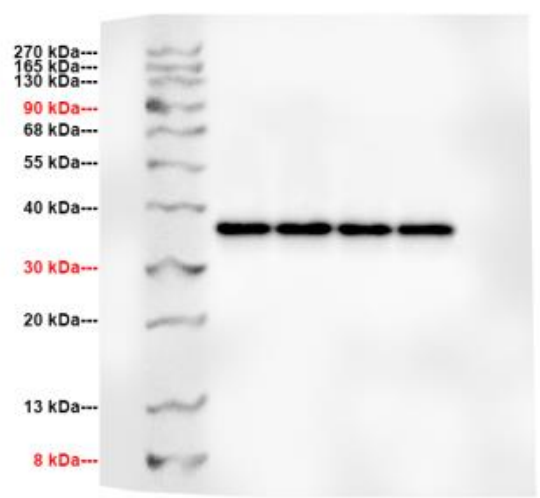

Figure4 C GPX4

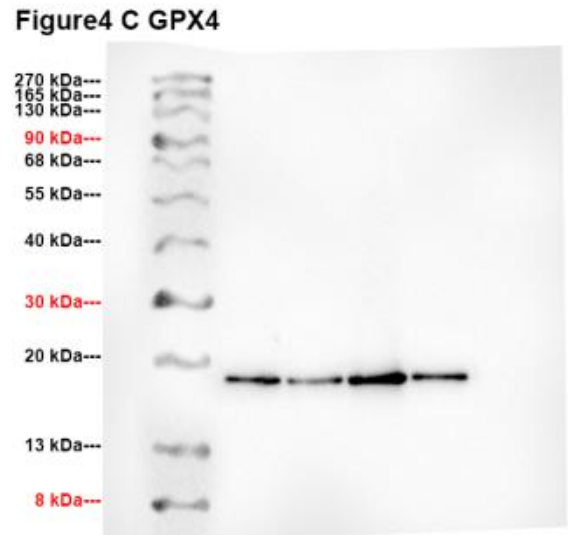

Figure4 C GAPDH

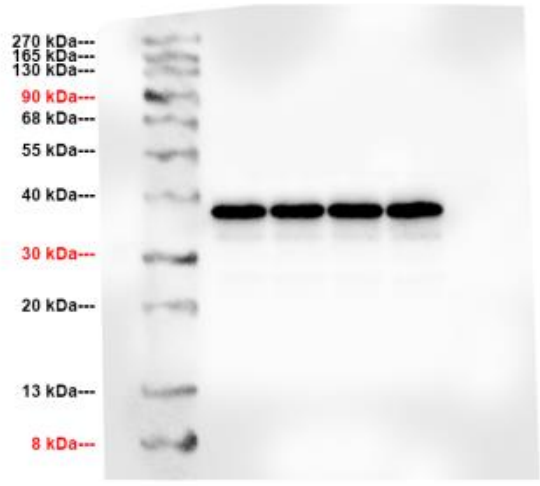

Figure4 C FTH

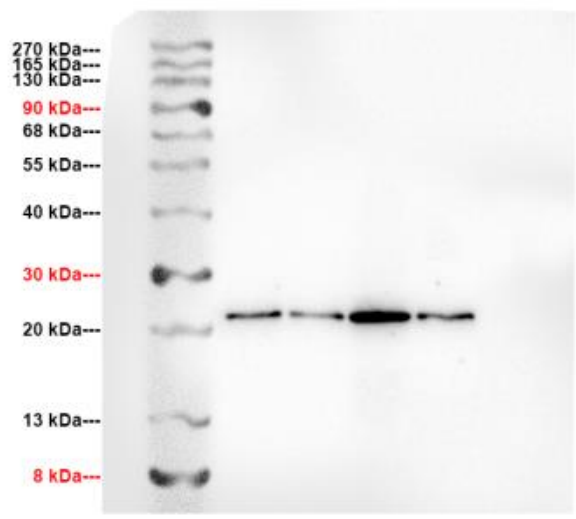

Figure4 C ACSL4

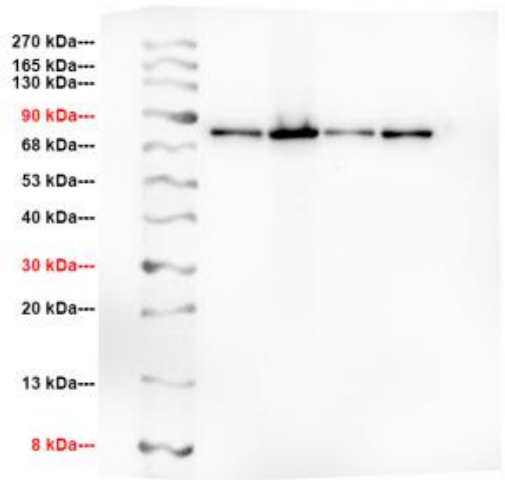

Figure4 D p62

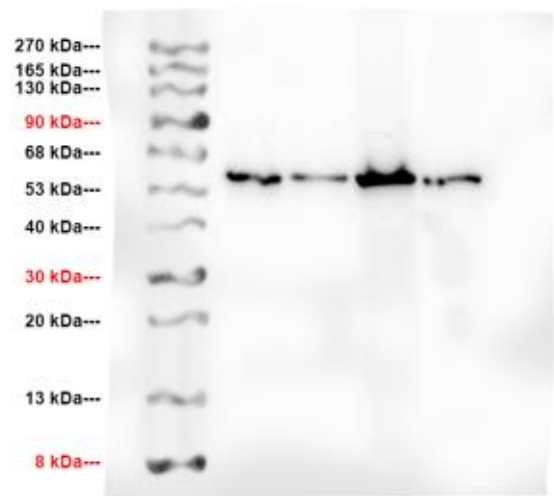

Figure4 D LC3

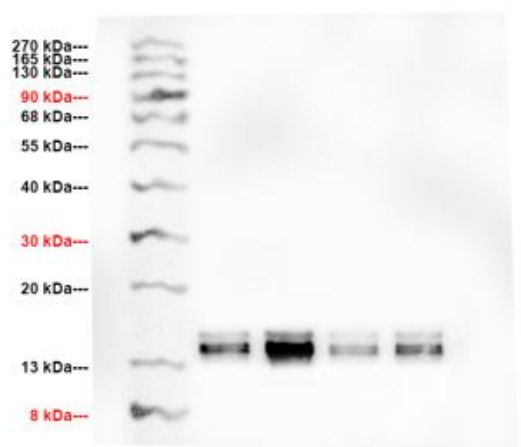

Figure4 D GAPDH

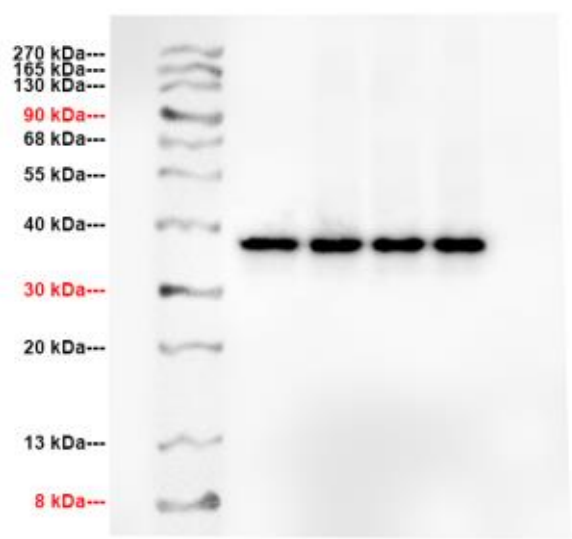

Figure4 D Beclin1

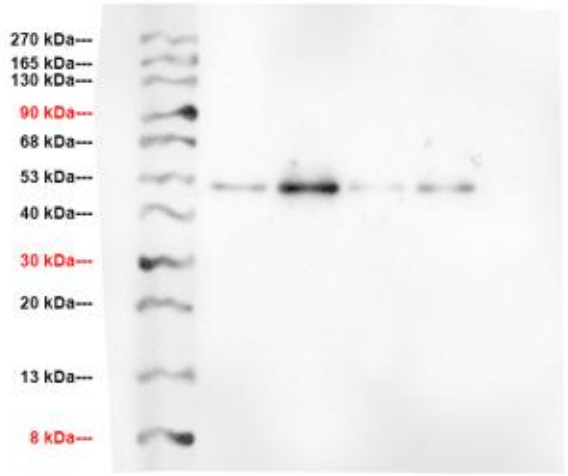

Figure4 F GPX4

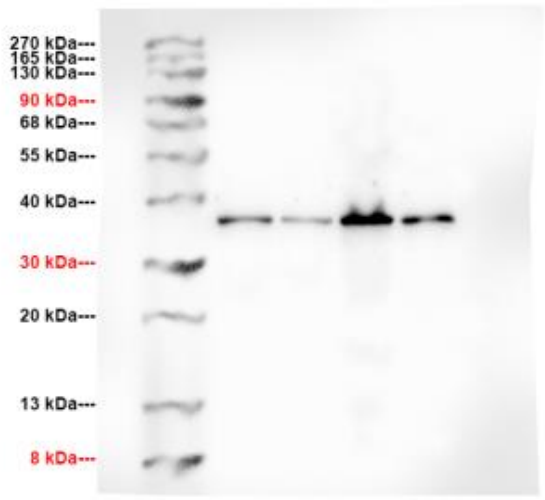

Figure4 F GAPDH

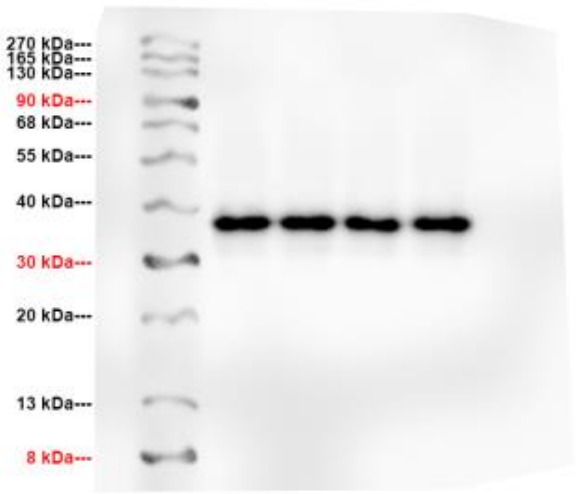

Figure4 F FTH1

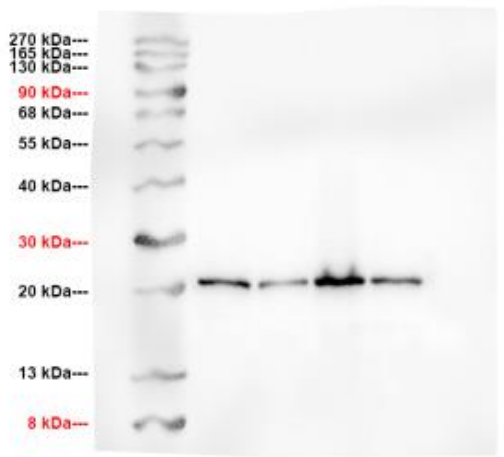

Figure4 F ACSL4

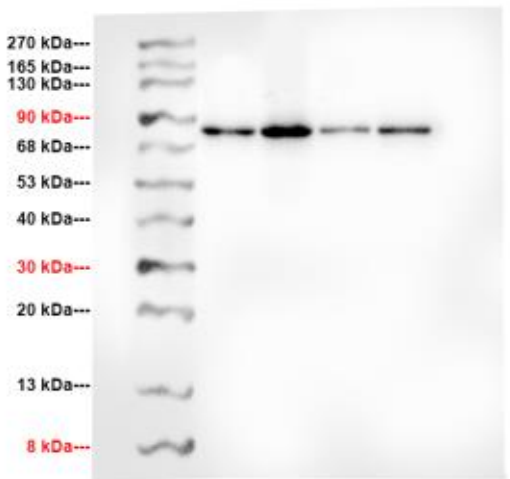

Figure4 D p62

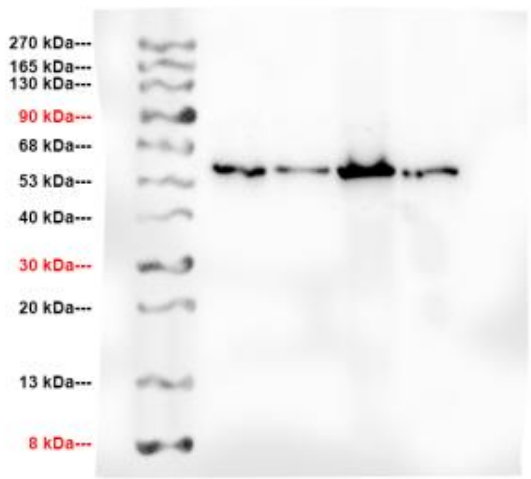

Figure4 D LC3

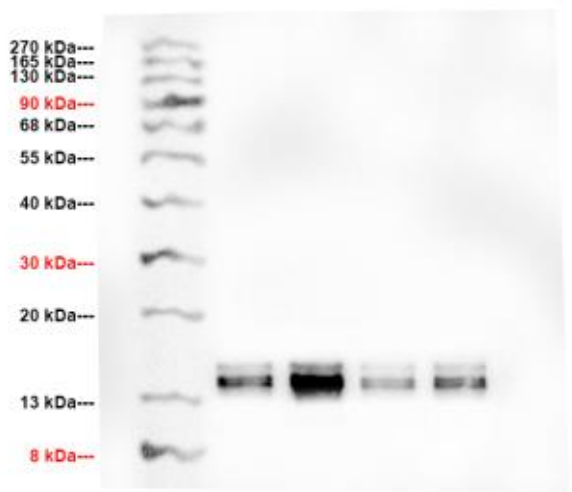

Figure4 D GAPDH

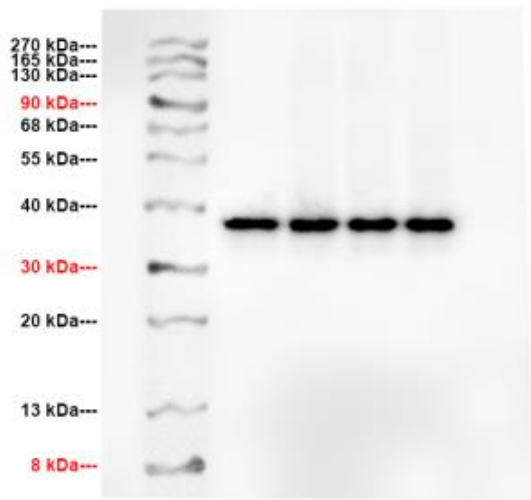

Figure4 D Beclin1

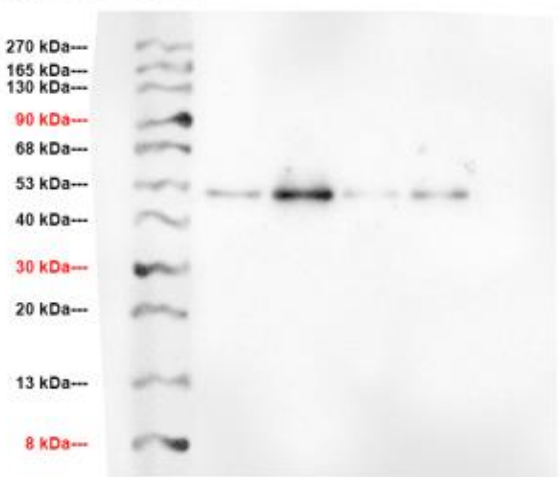

Figure4 F GPX4

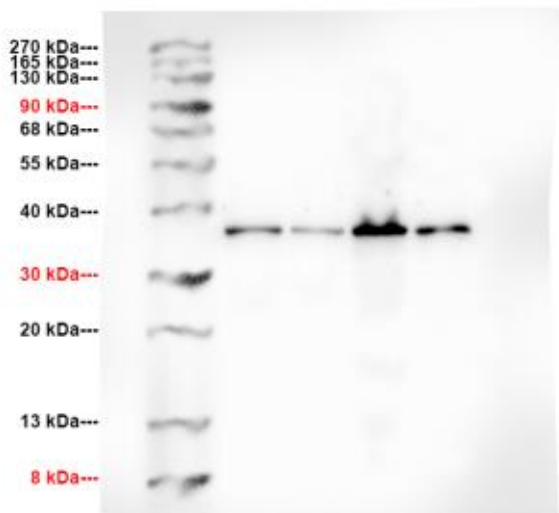

Figure4 F GAPDH

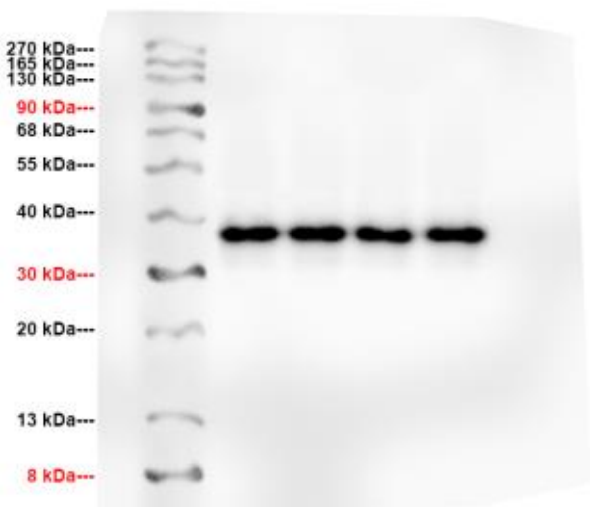

Figure4 F FTH1

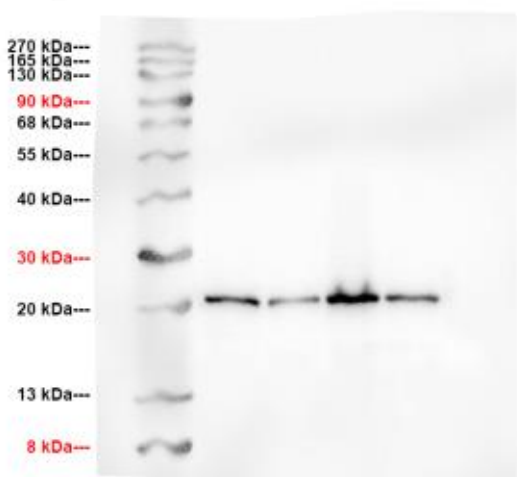

Figure4 F ACSL4

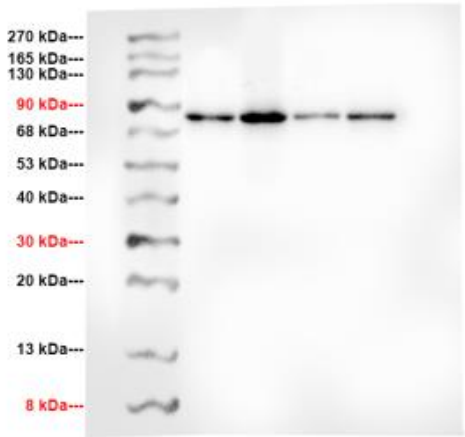

Figure 5 B Case1-5 p62

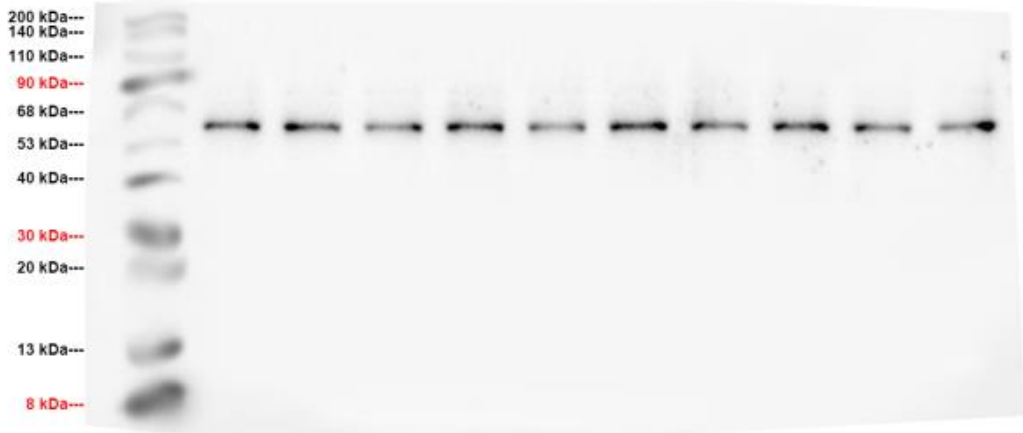

Figure 5 B Case1-5 GAPDH

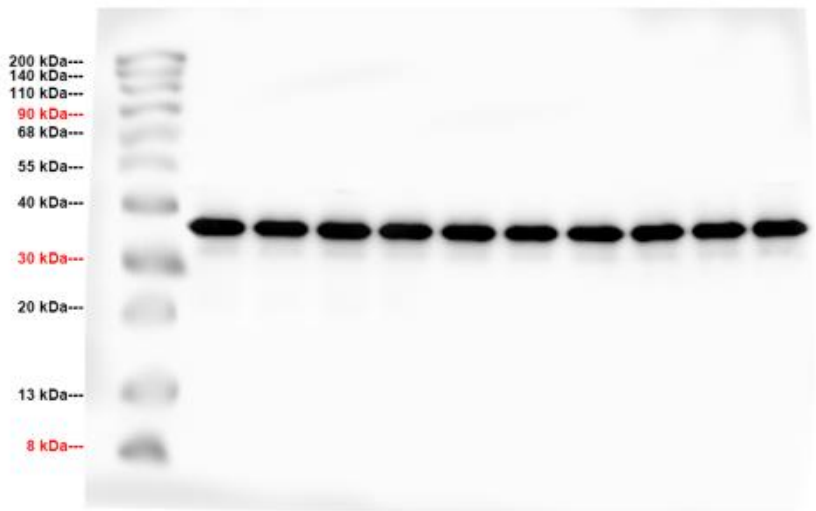

Figure 5 B Case6-10 p62

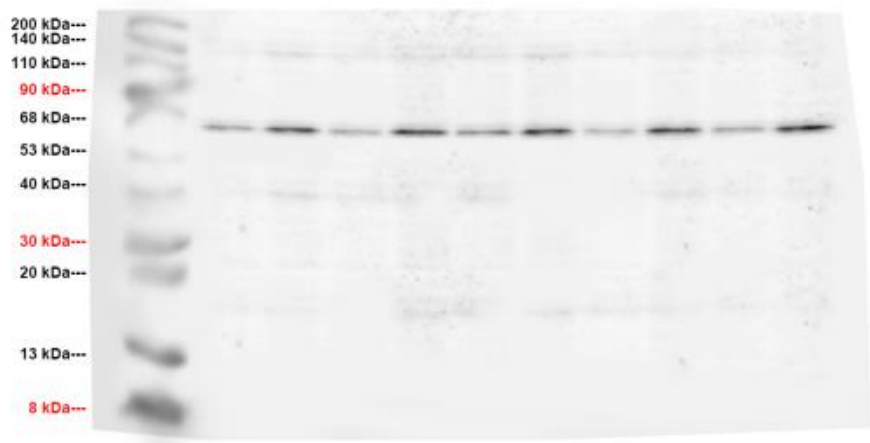

Figure5 B GAPDH

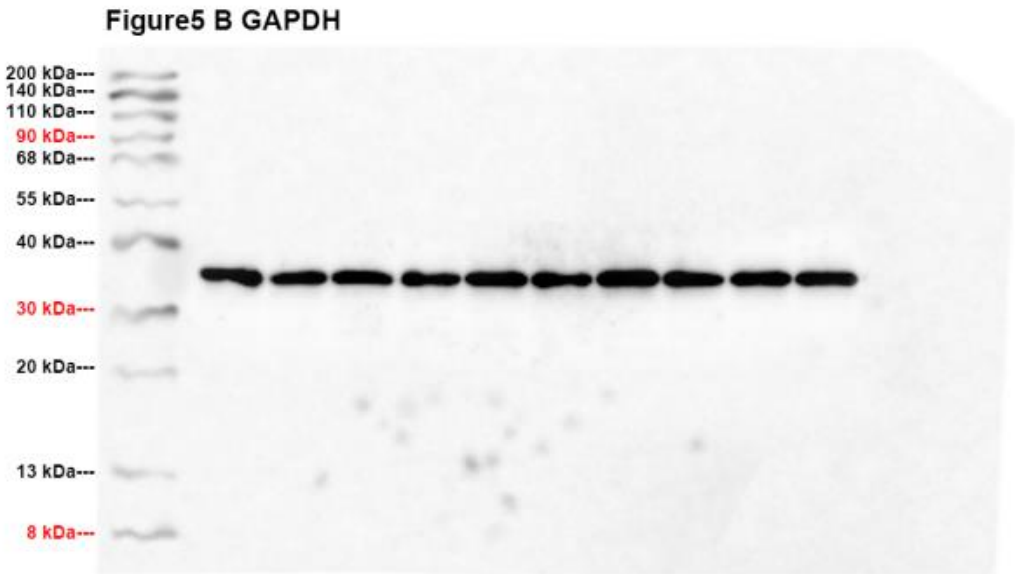

Figure 5 C Hep3B p62

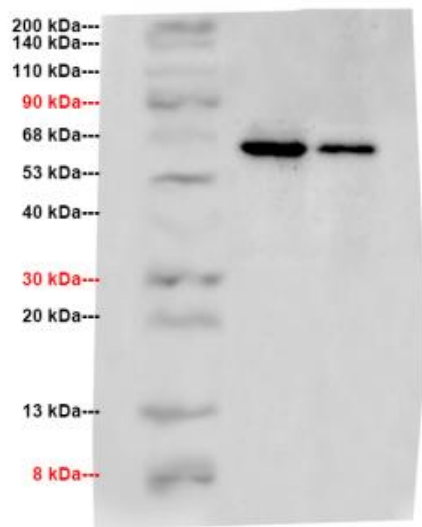

Figure 5 C Hep3B GAPDH

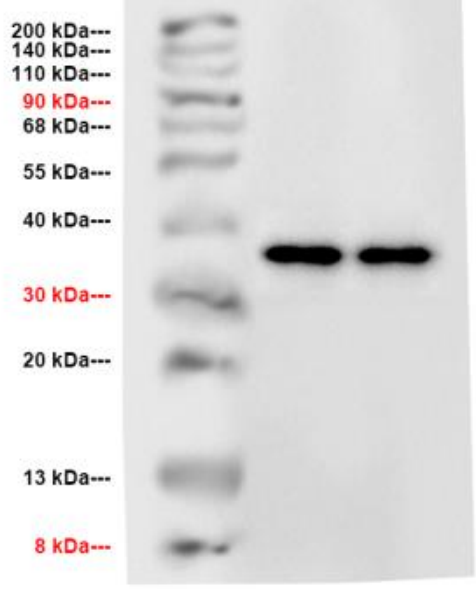

Figure 5 C MHCC97H p62

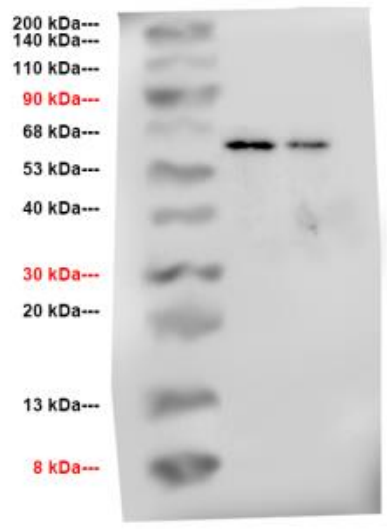

Figure 5 C MHCC97H GAPDH

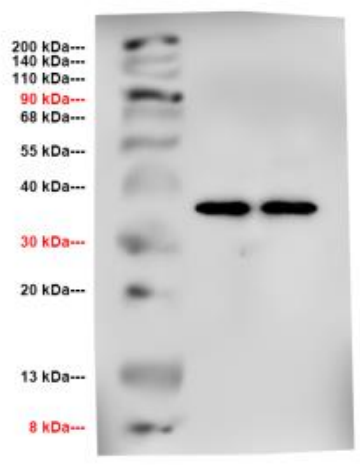

Figure 5 D sh-2 p62

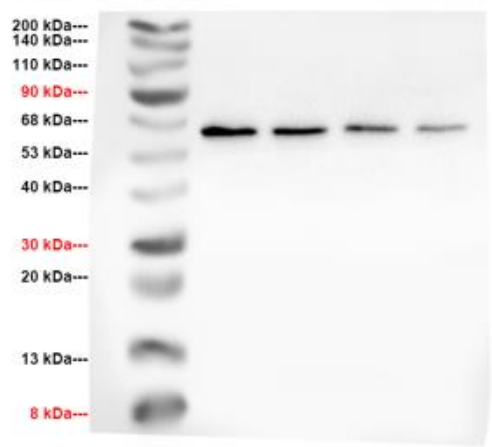

Figure 5 D sh2+3-MA p62

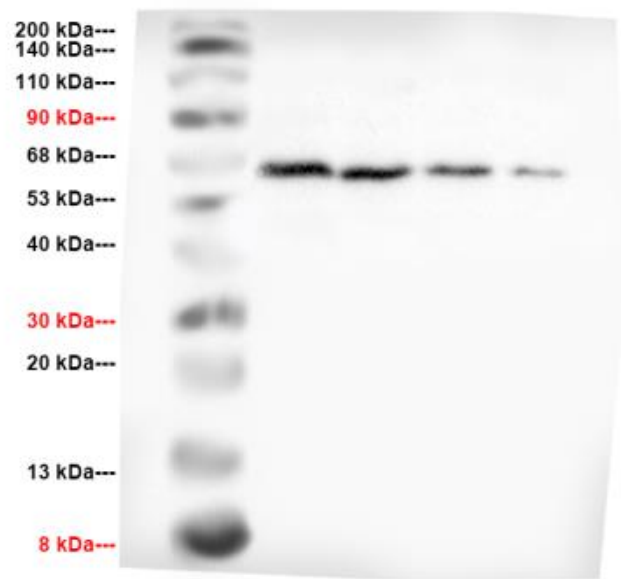

Figure 5 D sh-2 GAPDH

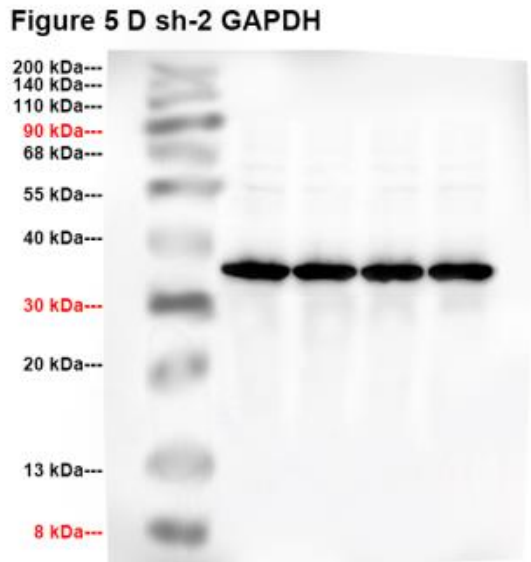

Figure 5 D sh2+CQ p62

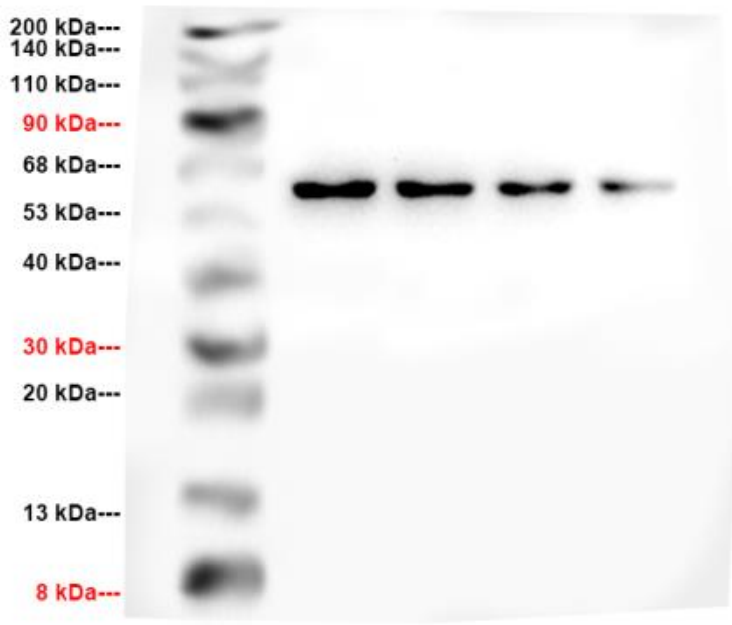

Figure 5 D sh2+3-MA GAPDH

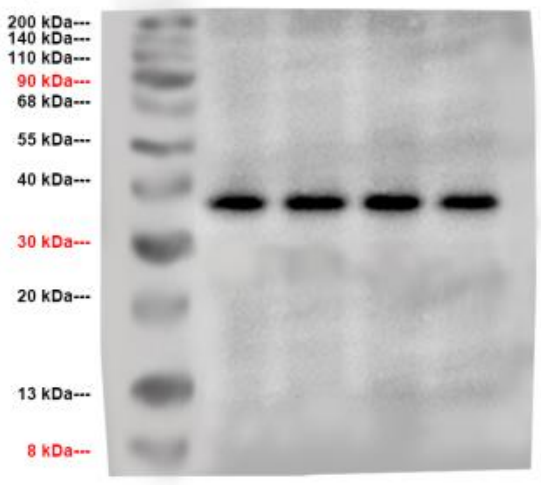

Figure 5 D sh2+MG132 p62

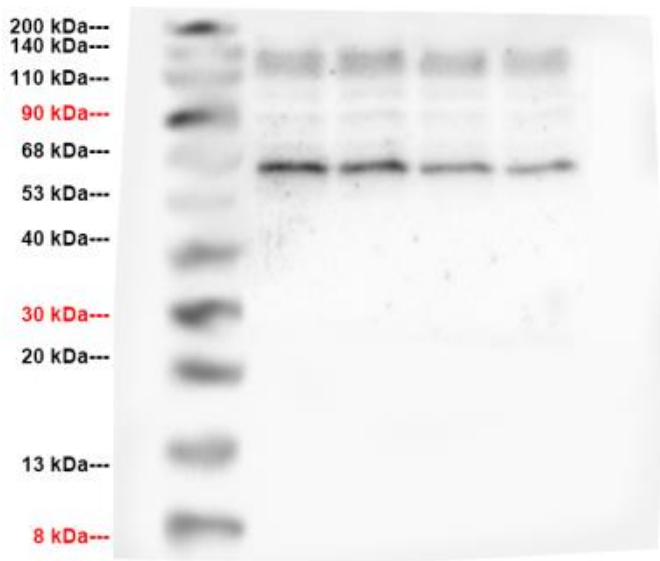

Figure 5 D sh2+CQ GAPDH

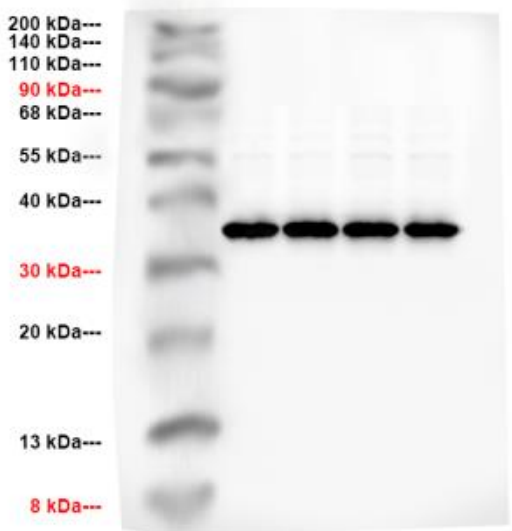

Figure 5 D shCtrl p62

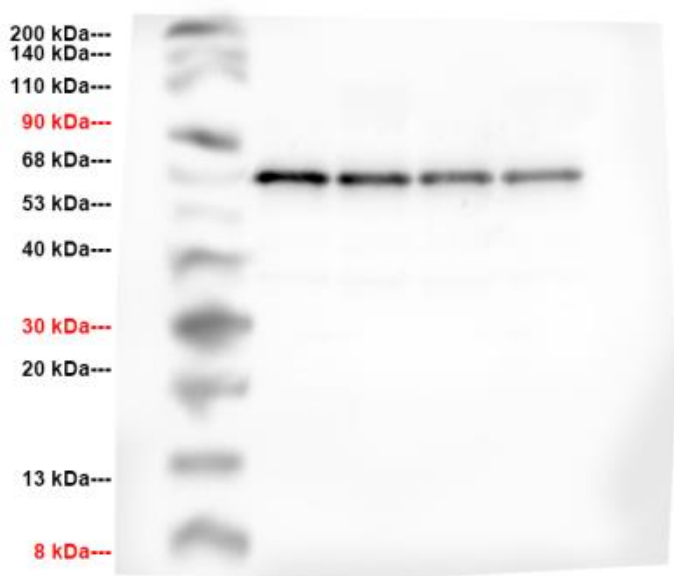

Figure 5 D sh2+MG132 GAPDH

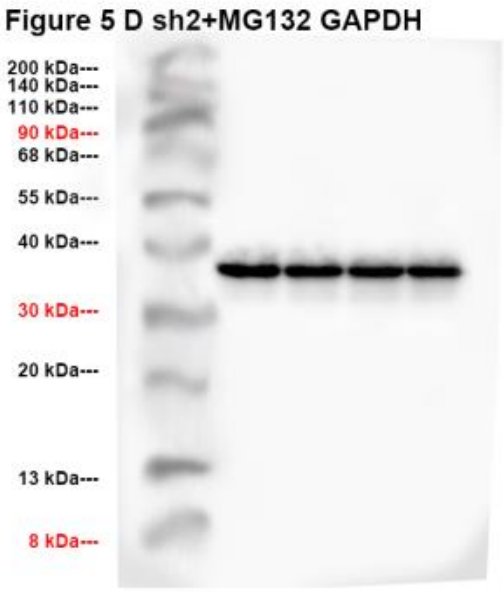

Figure 5 D shCtrl GAPDH

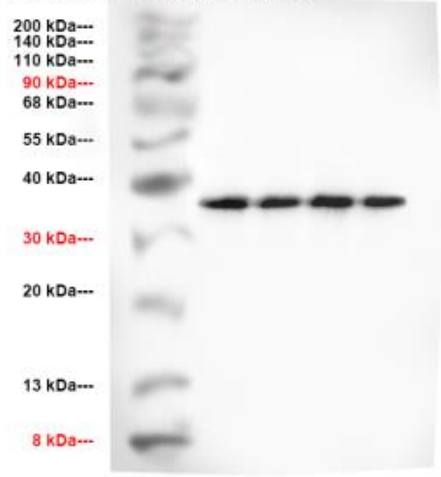

Figure 5 E p62

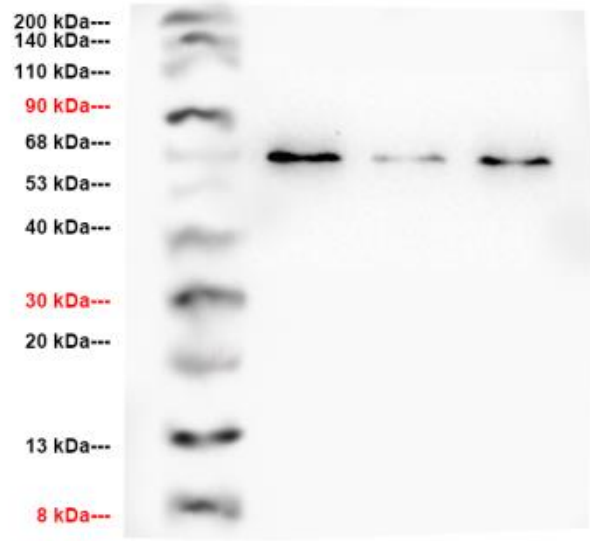

Figure 5 E OTUB1

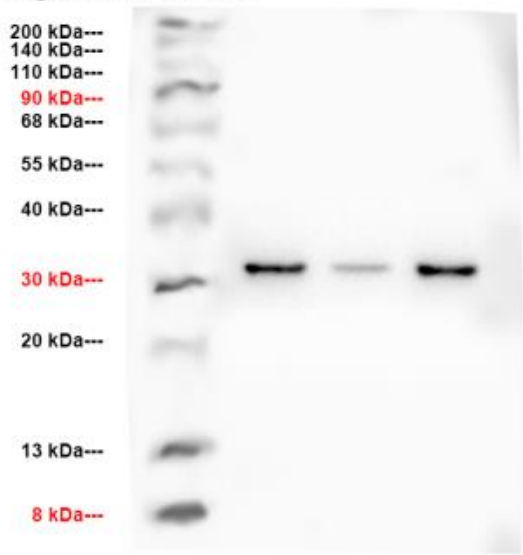

Figure 5 E GAPDH

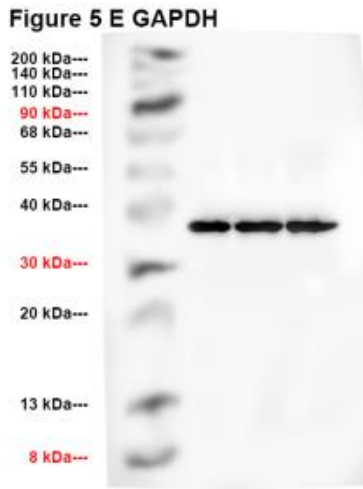

Figure5 F p62

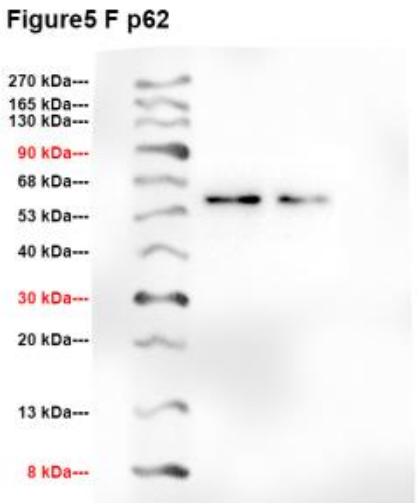

Figure5 F OTUB1

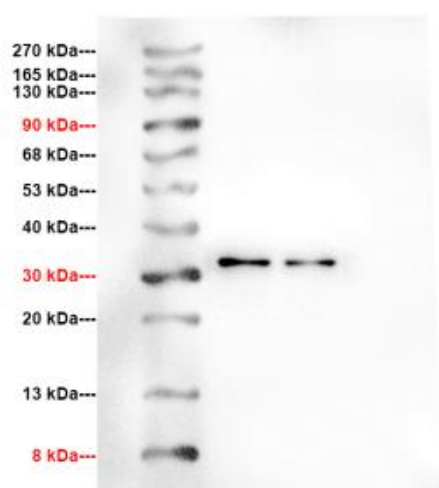

Figure5 F IB:Ub

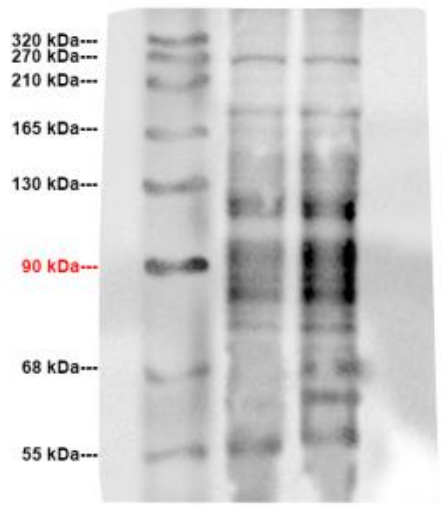

Figure4 F GAPDH

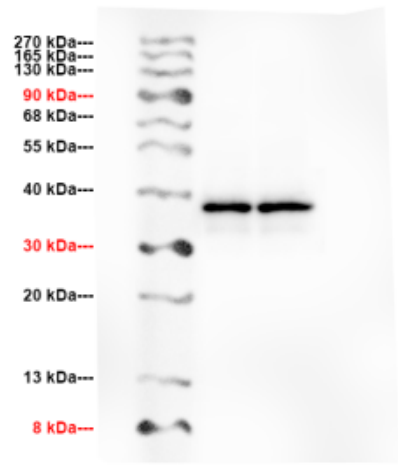

Figure5 G Lys6 IB Myc

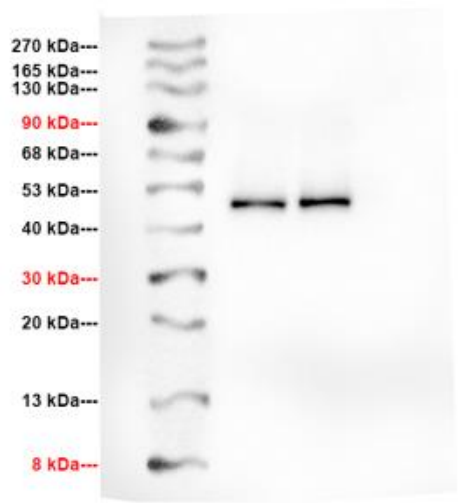

Figure5 G Lys6 IBHA

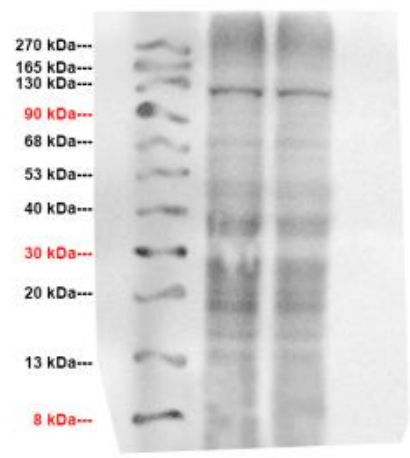

Figure5 G Lsy6 GAPDH

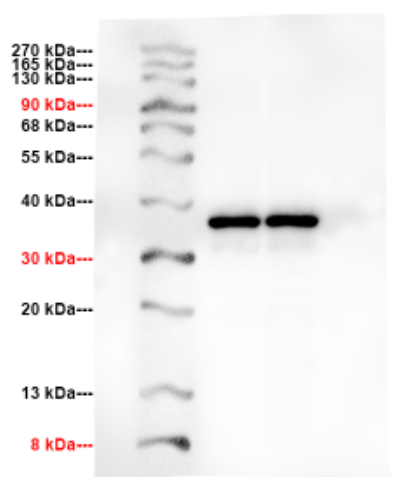

Figure5 G Lys6 Anti-Flag

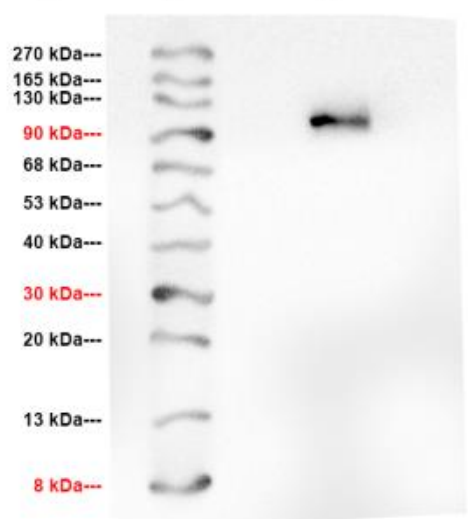

Figure5 G Lys48 IB Myc

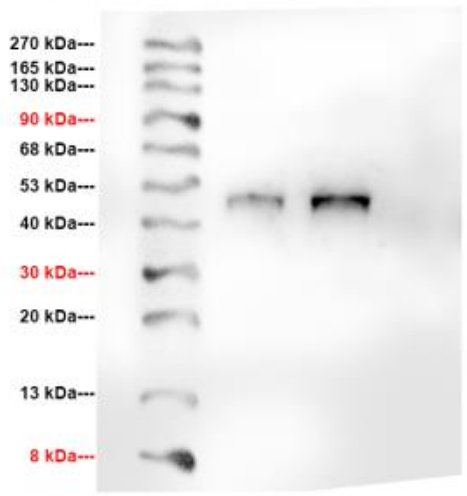

Figure5 G Lys48 IB HA

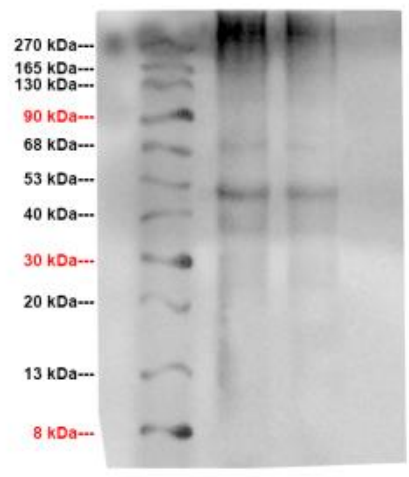

Figure5 G Lys48 GAPDH

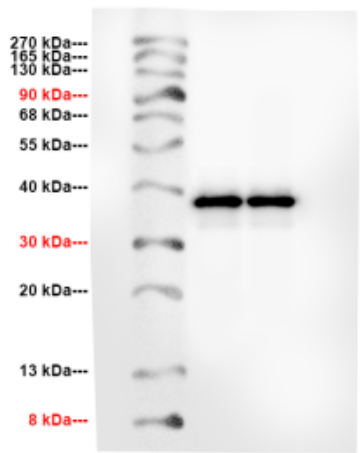

Figure5 G Lys48 Anti-Flag

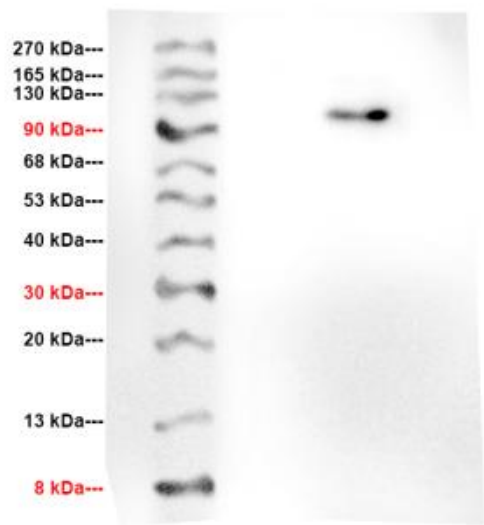

Figure5 G Lys63 IB Myc

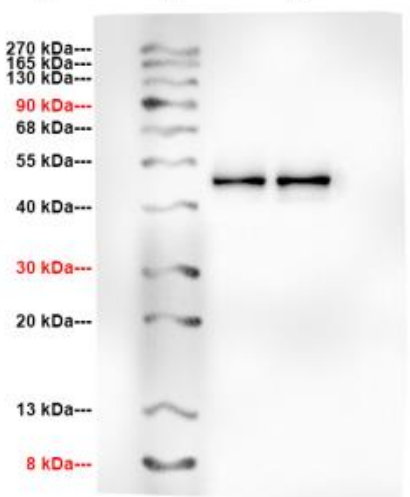

Figure5 G Lys63 IB HA

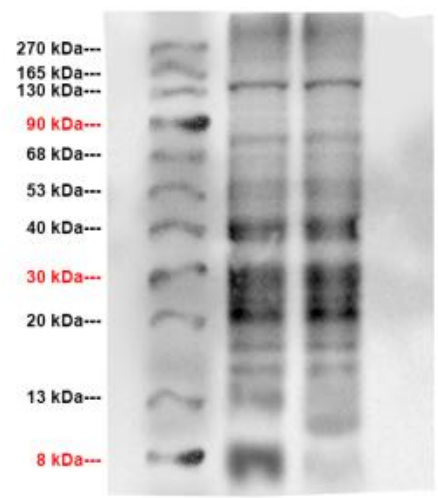

Figure5 G Lys63 GAPDH

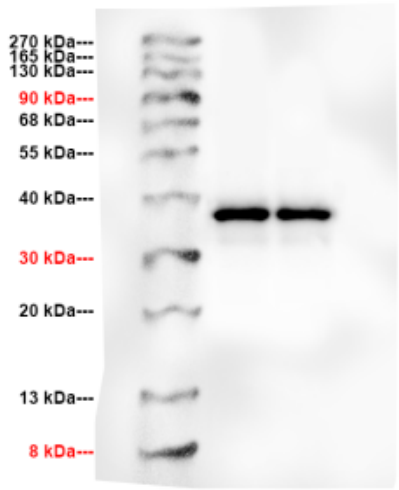

Figure5 G Lys63 Anti-Flag

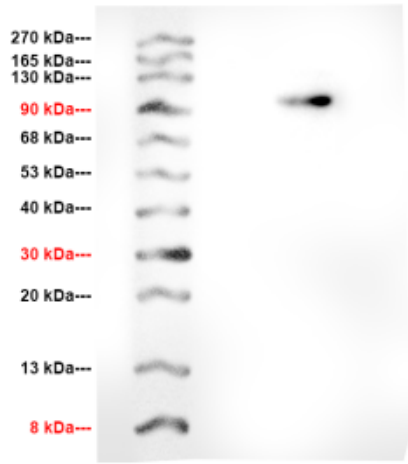

Figure5 G WT IB Myc

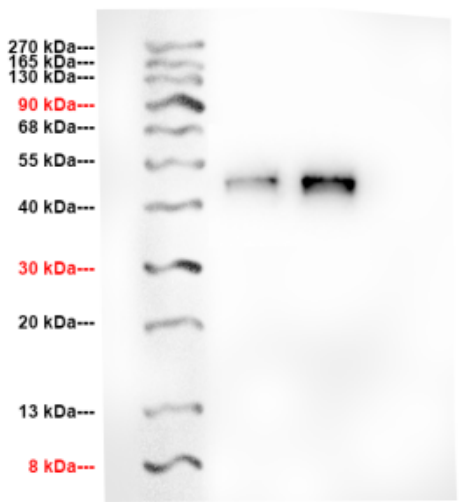

Figure5 G WT IB HA

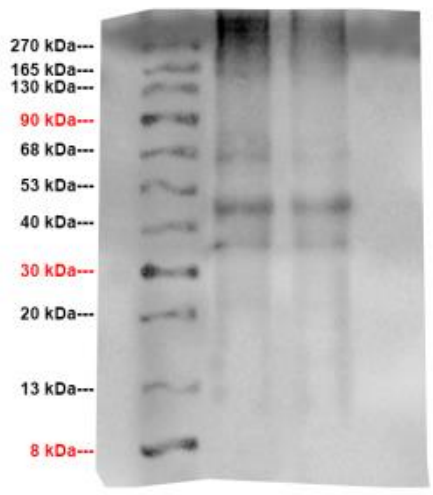

Figure5 G WT Anti-Flag

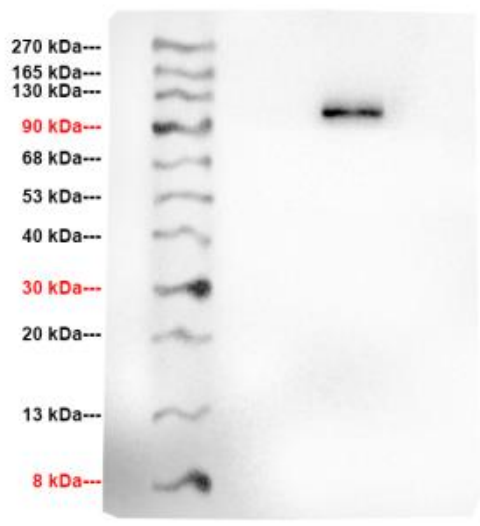

Figure5 G WT GAPDH

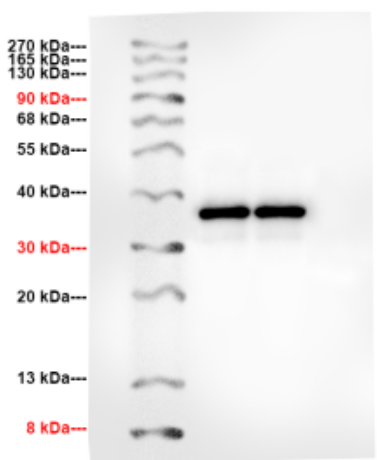

Figure5 H p62

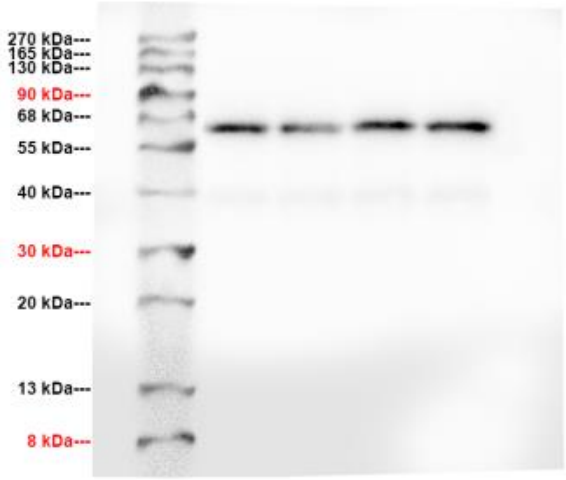

Figure5 H OTUB1

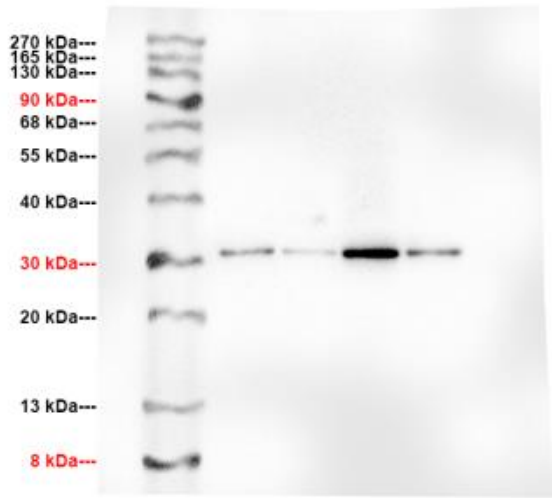

Figure5 H GAPDH

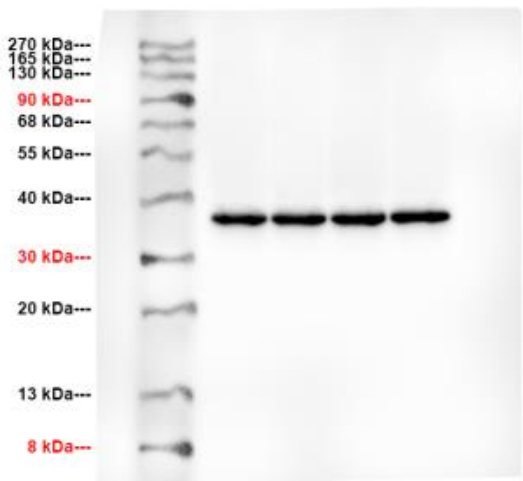

Figure5 I IBHA

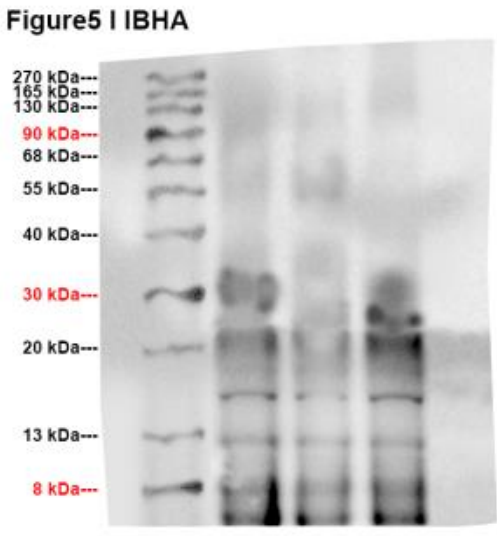

Figure5 I IB Myc

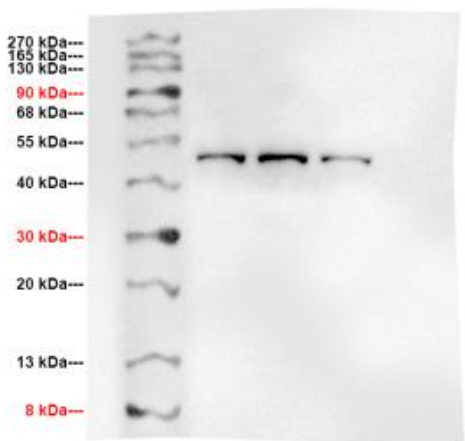

Figure5 I GAPDH

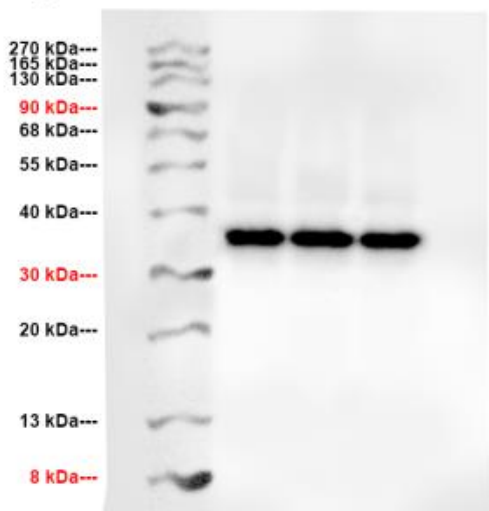

Figure5 I Anti-Flag

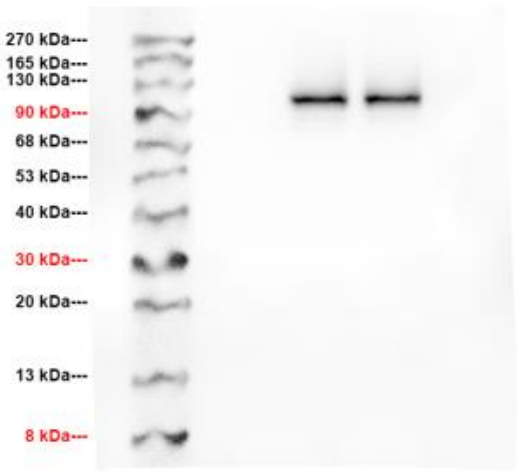

Figure5 J OTUB1

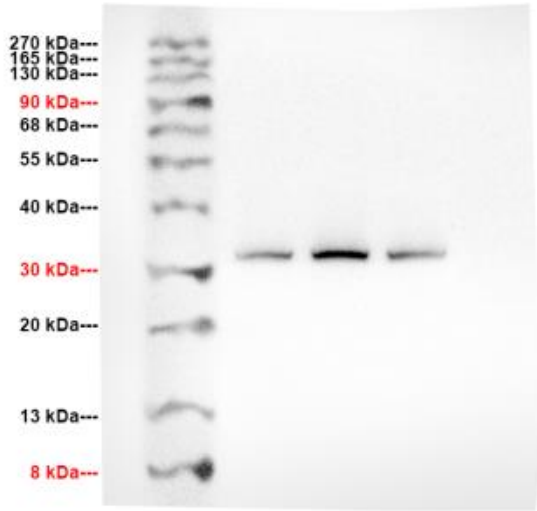

Figure5 J IB Ub

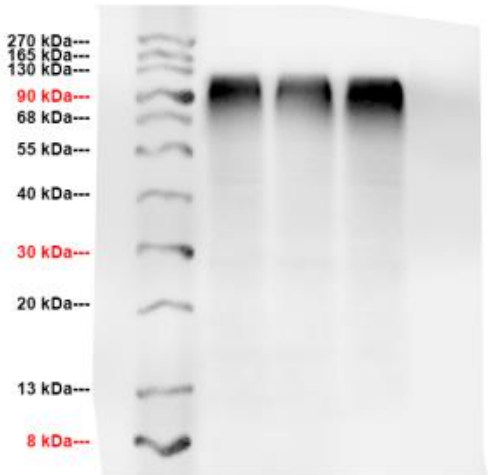

Figure5 J IB OTUB1

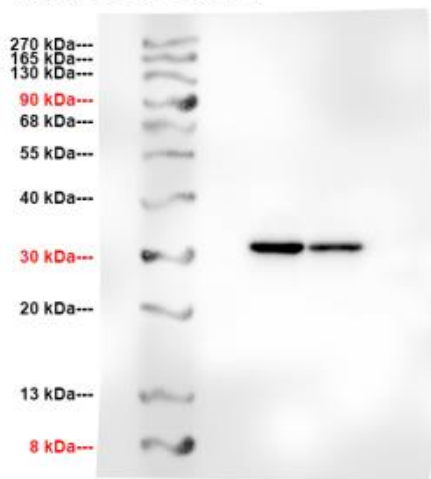

Figure5 J GAPDH

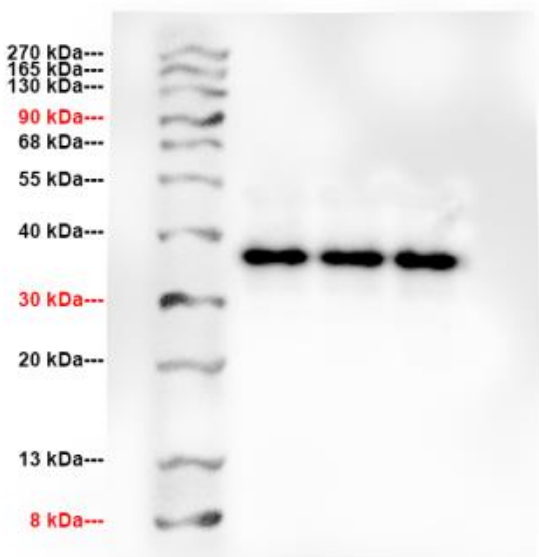

Figure 5-K-GPX4

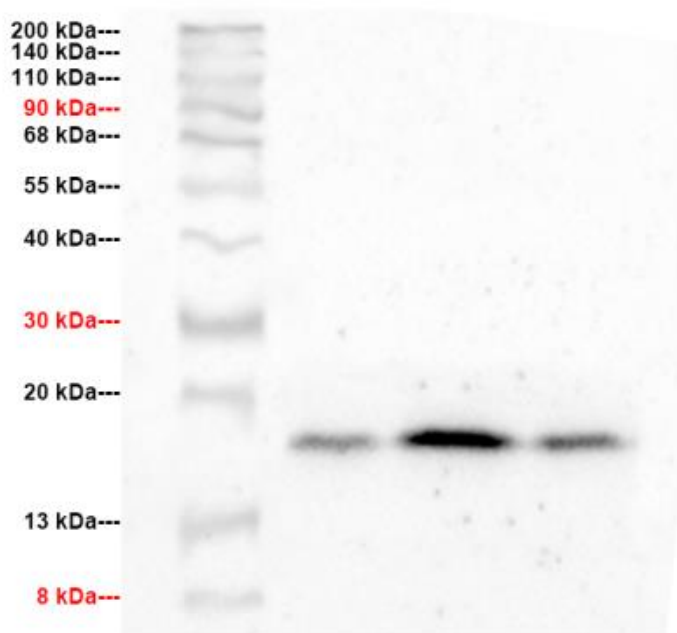

Figure 5-K-GAPDH

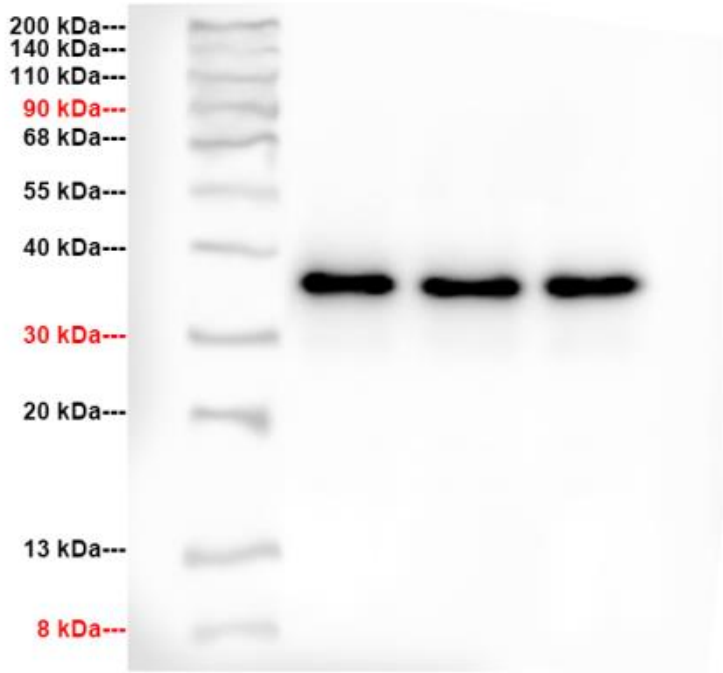

Figure 5-K-FTH1

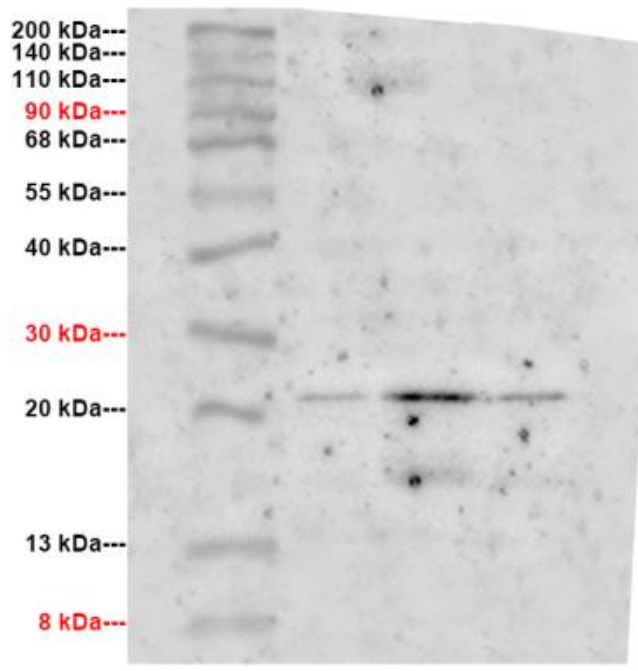

Figure 5-K-ACSL4

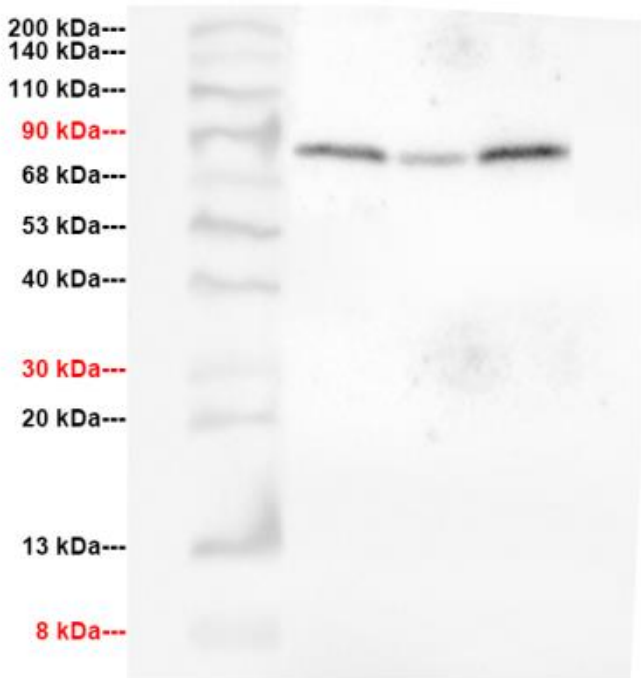

Figure 5 P p62

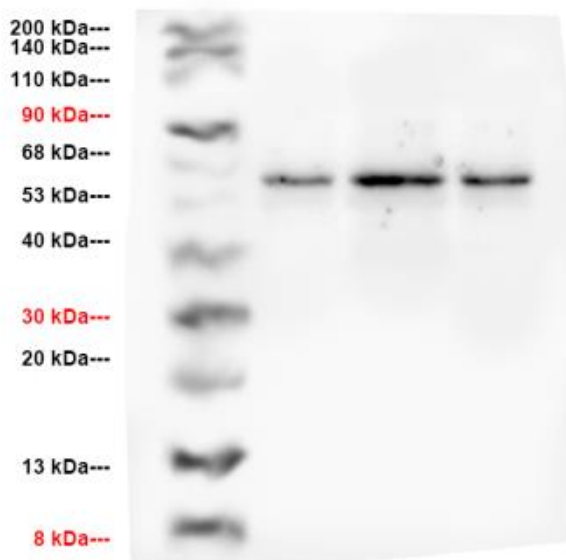

Figure 5 P GAPDH

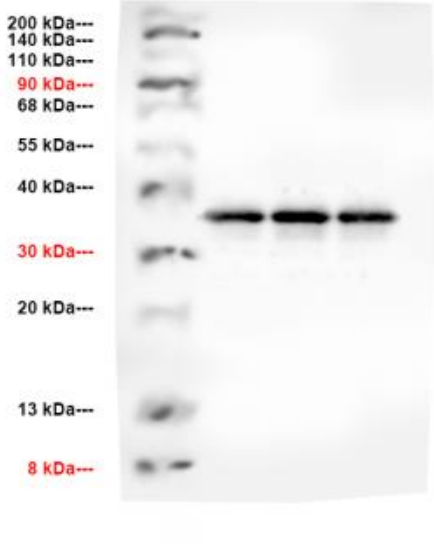

Figure5 Q GAPDH

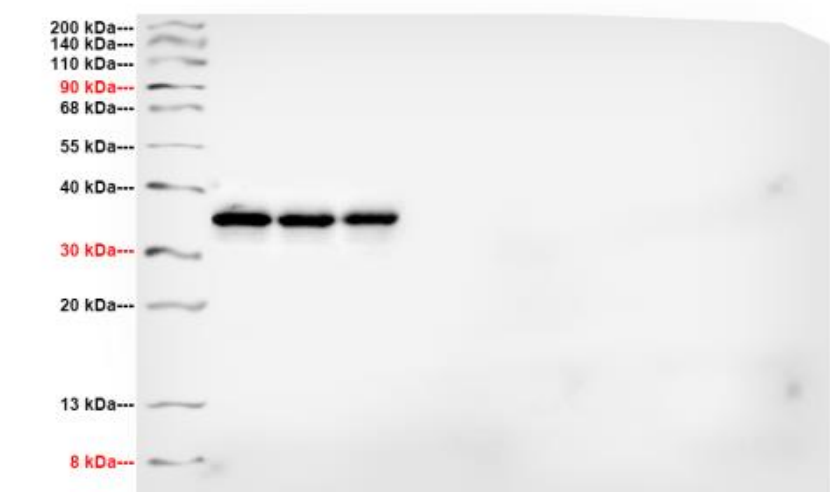

Figure5 Q Beclin 1

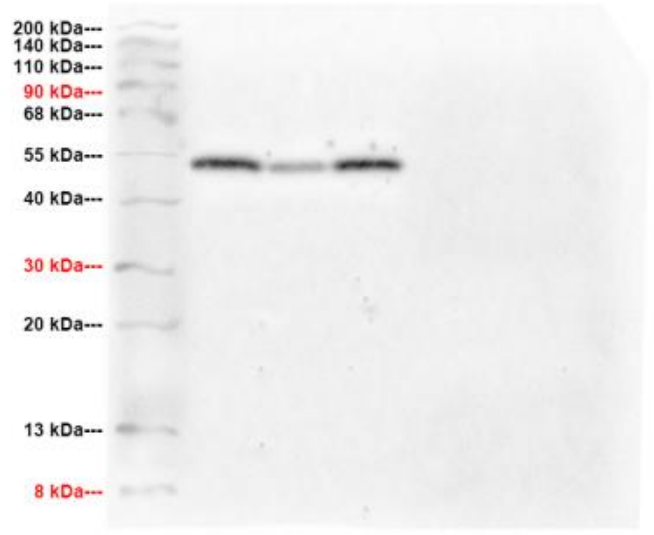

Figure5 Q ATG7

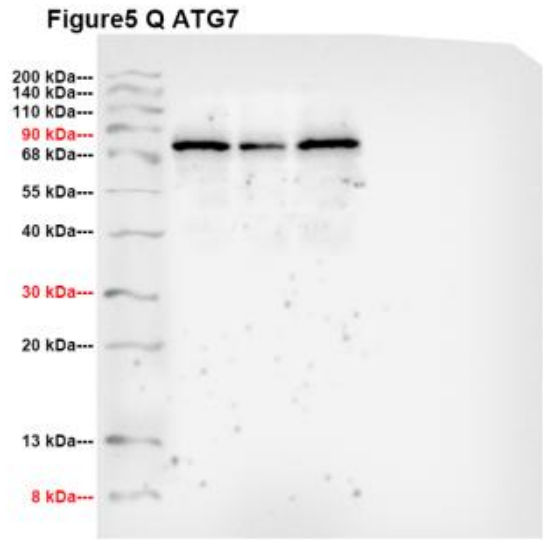

Figure5 Q ATG5

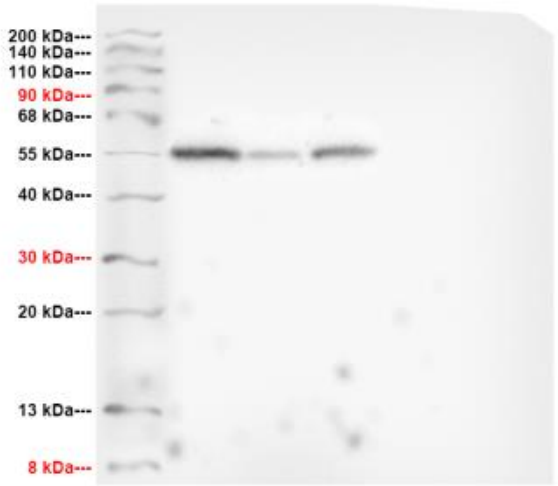

Figure 6-A-GAPDH

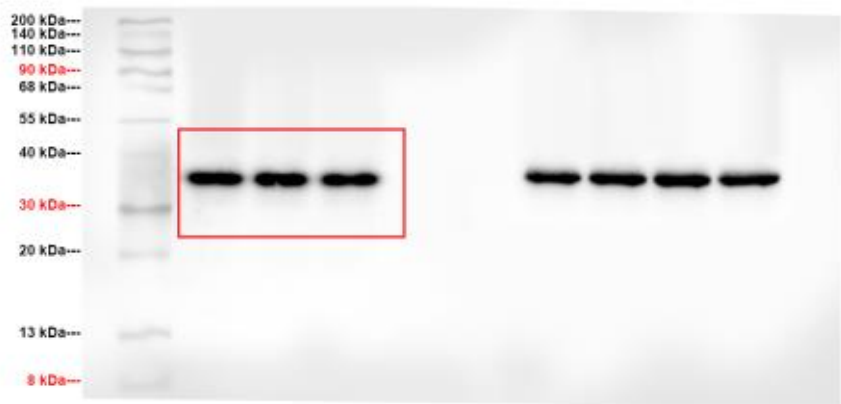

Figure 6-D-ACSL4

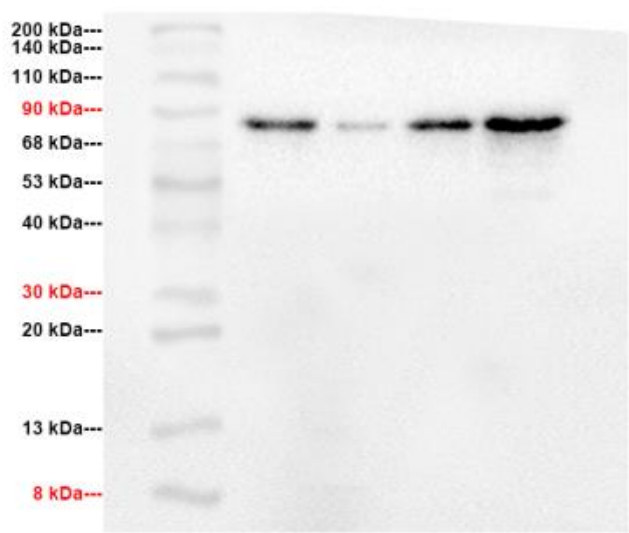

Figure 6-A-p62

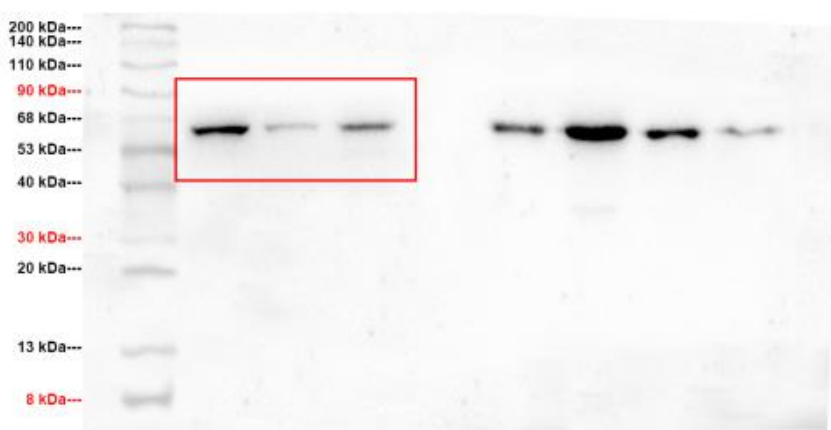

Figure 6-D-FTH1

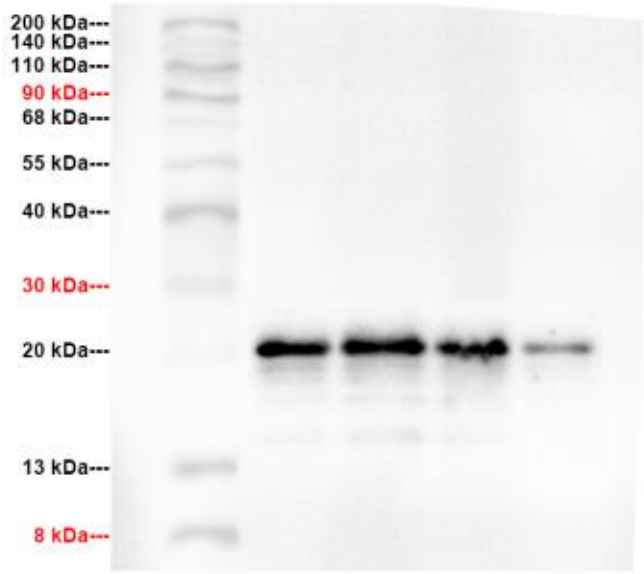

Figure 6-D-GAPDH

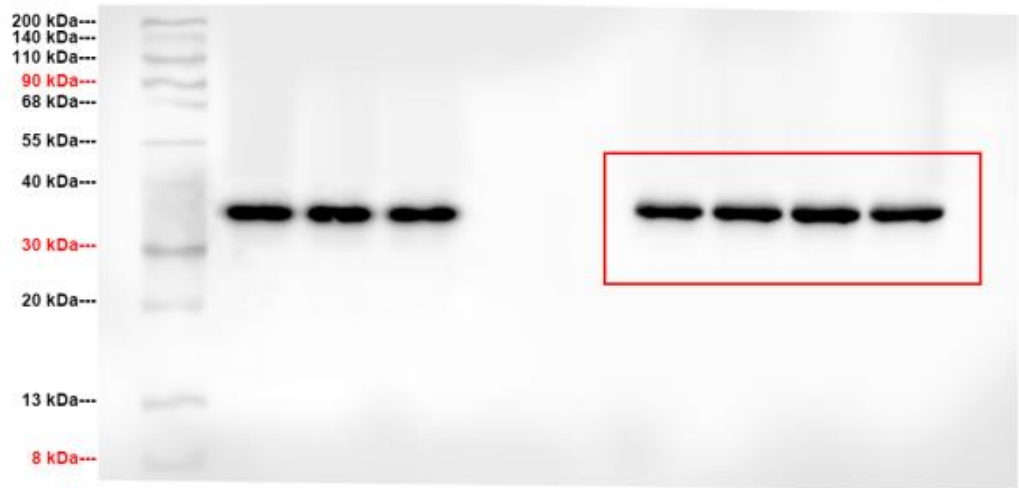

Figure 6-D-GPX4

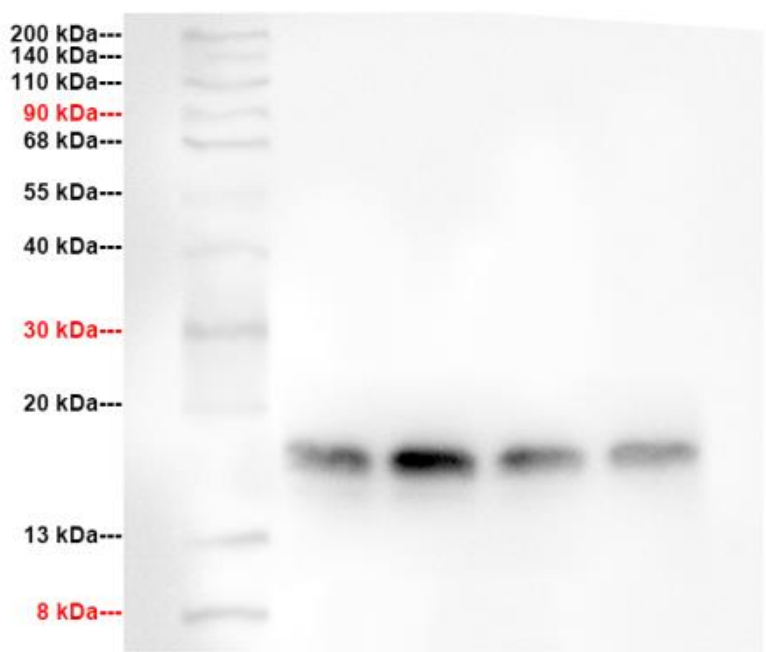

Figure 6-L-ATG5

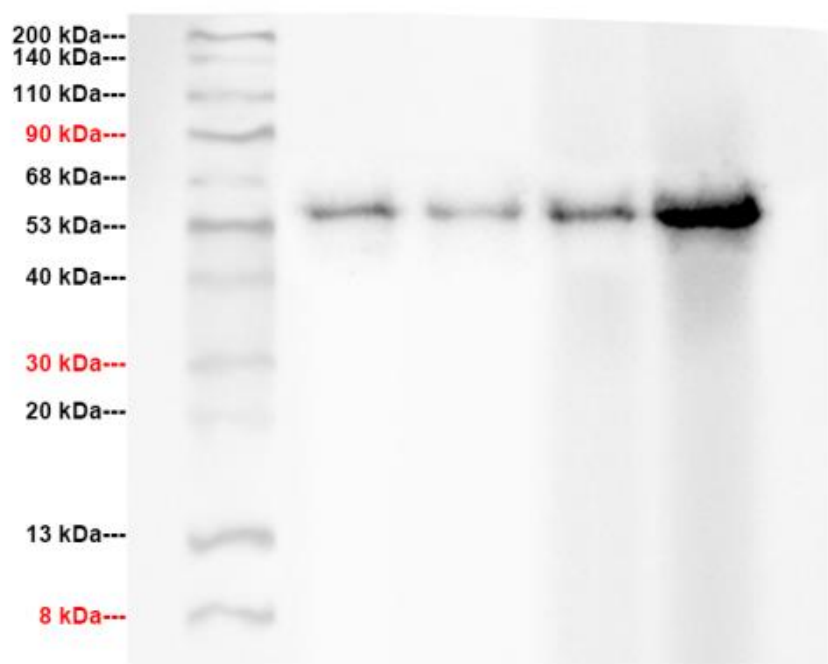

Figure 6-L-Beclin1

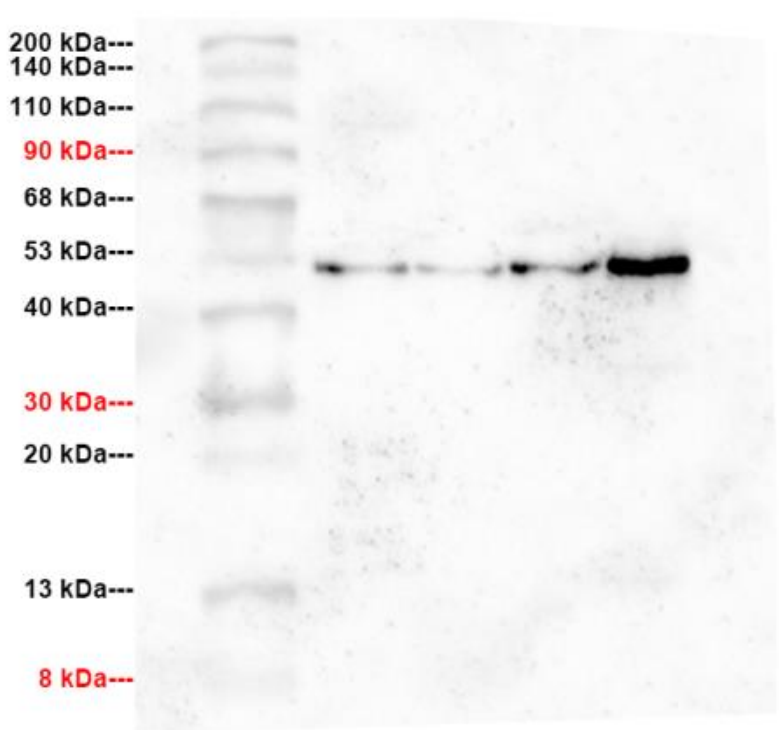

Figure 6-L-GAPDH

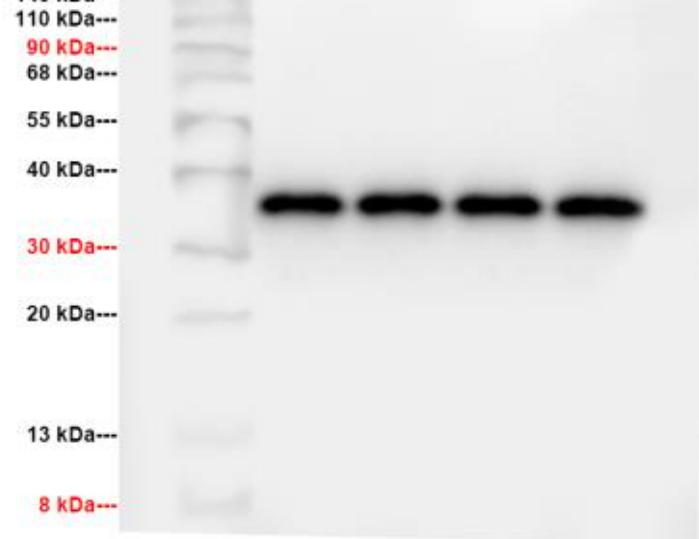

Figure 6-M-GAPDH

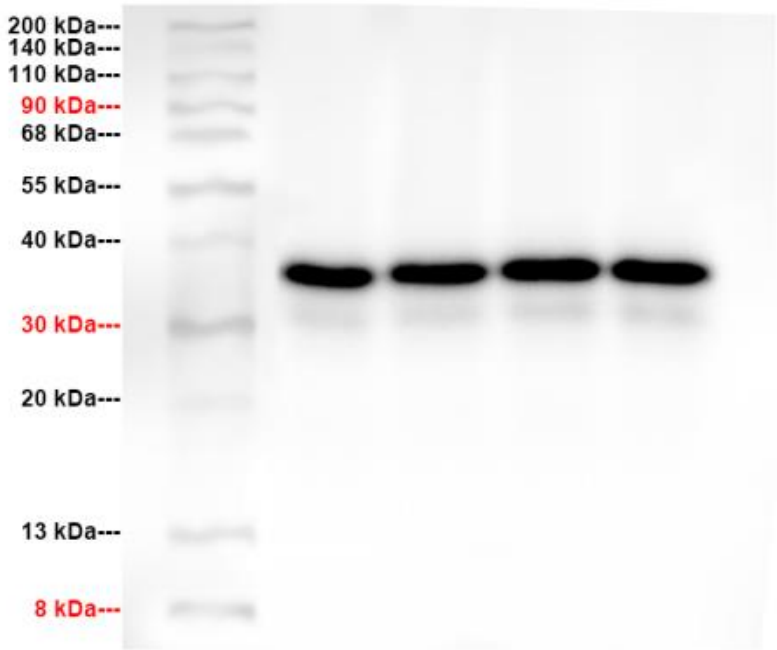

Figure 6-M-OTUB1

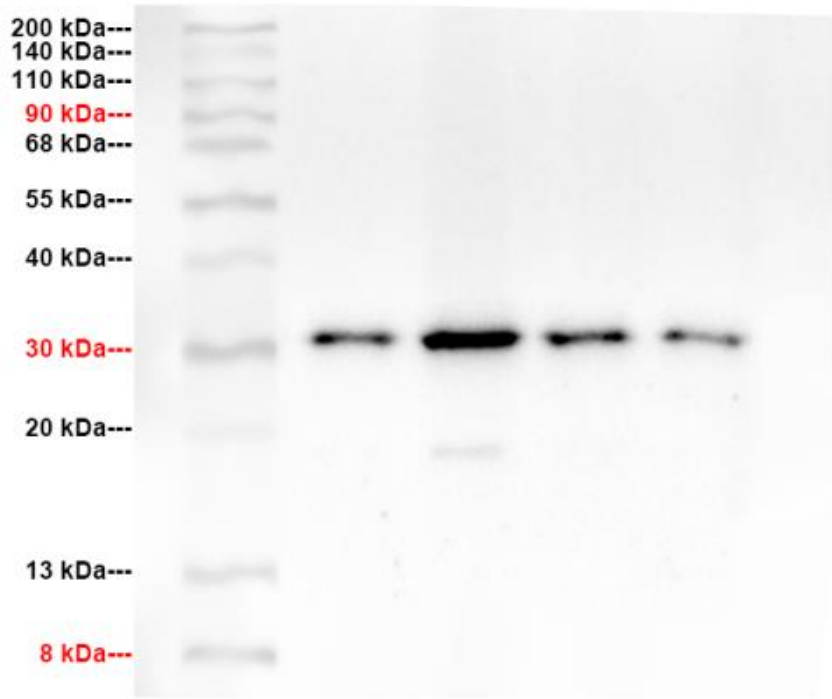

Figure 6-M-p62

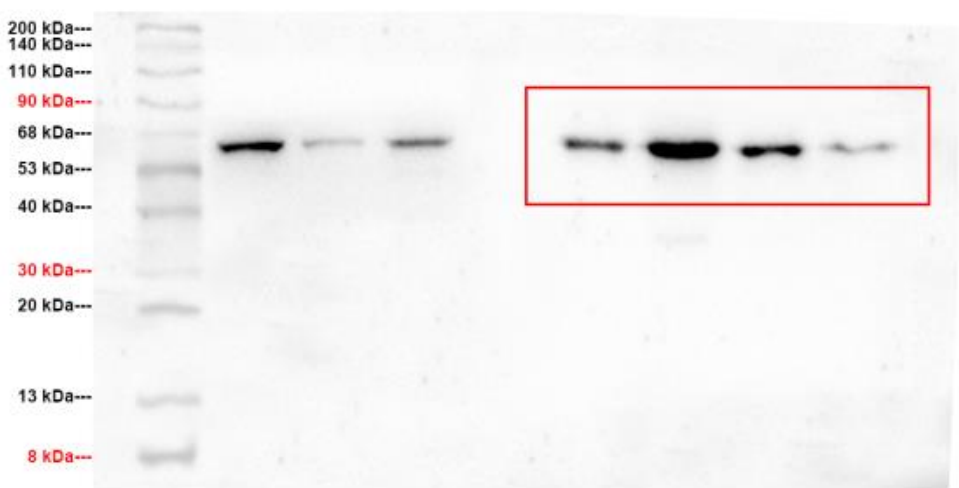

Figure-7(13)-B-ATG5

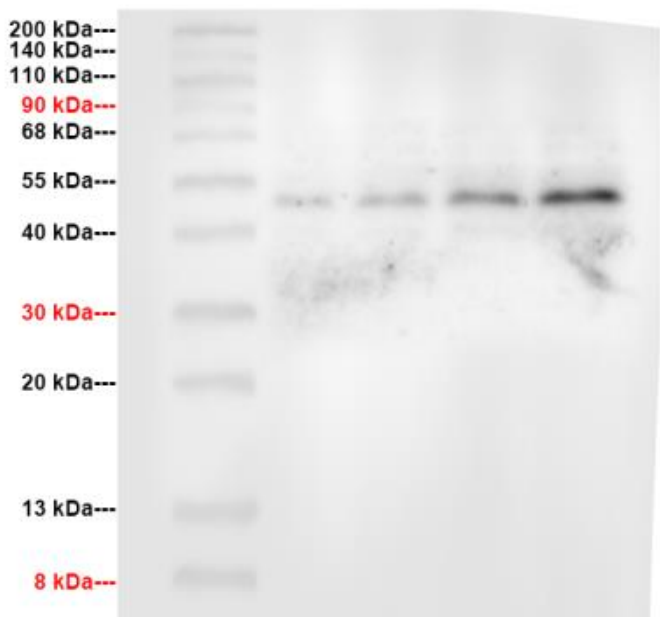

Figure7(13)-B-Beclin1

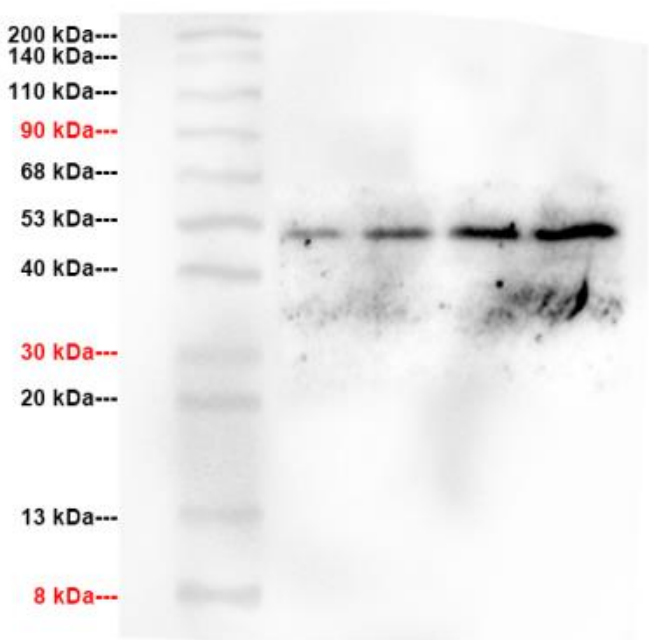

Figure7(13)-B-GAPDH

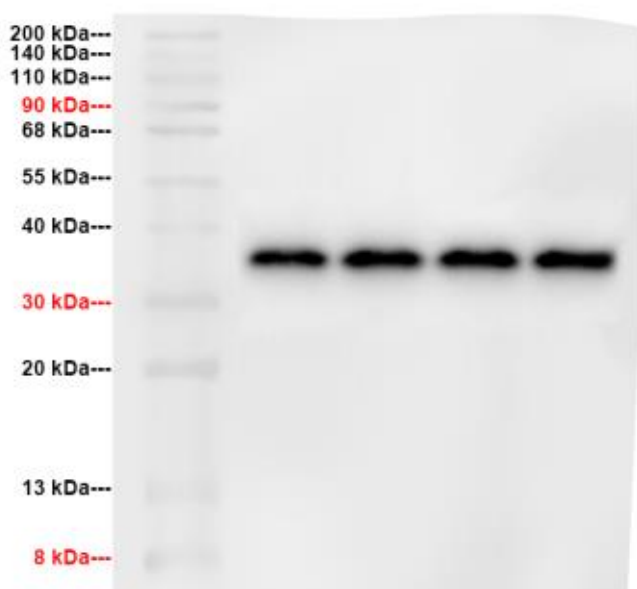

Figure7(13)-B-OTUB1

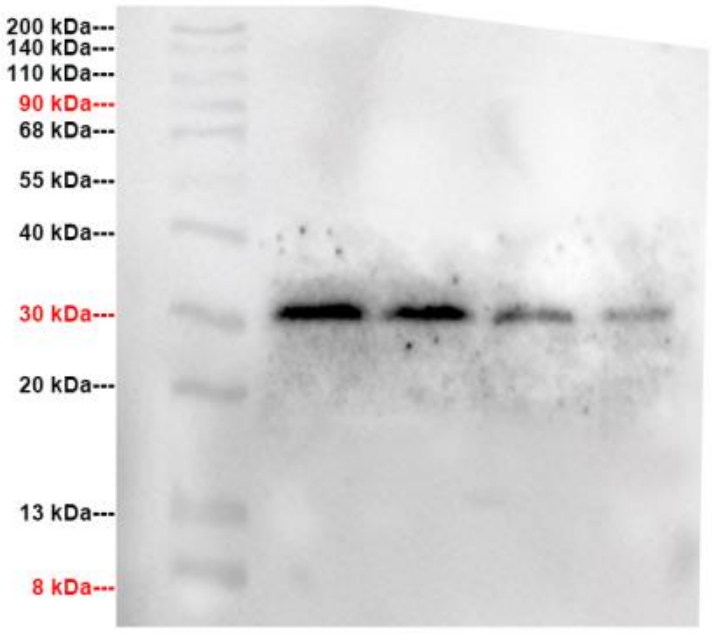

Figure7(13)-B-p62

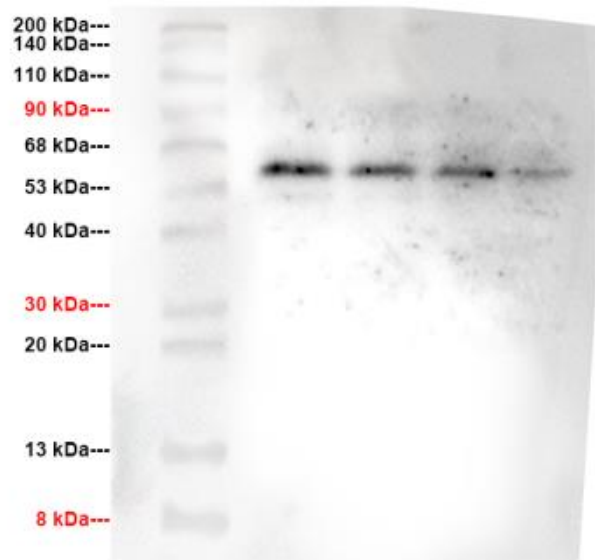

Figure-7(13)-D-ACSL4

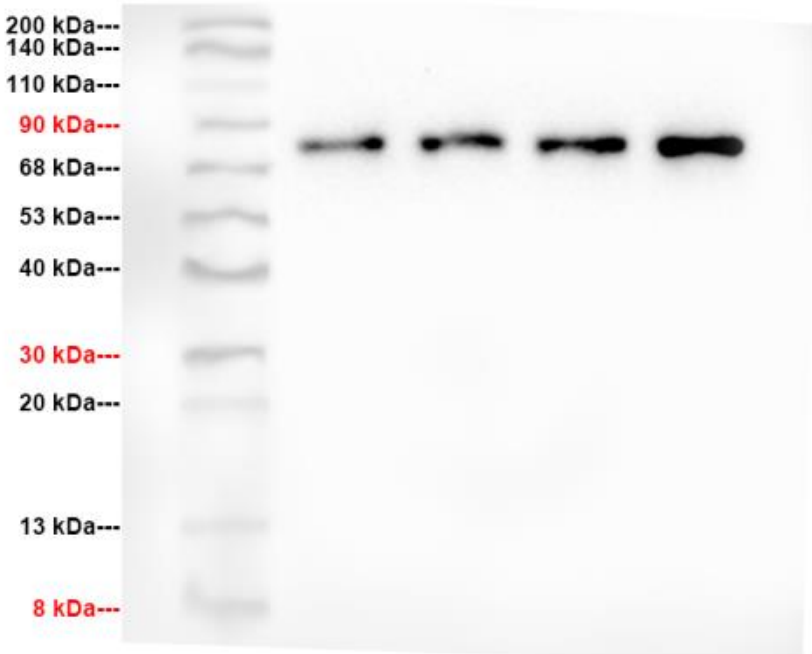

Figure-7(13)-D-FTH1

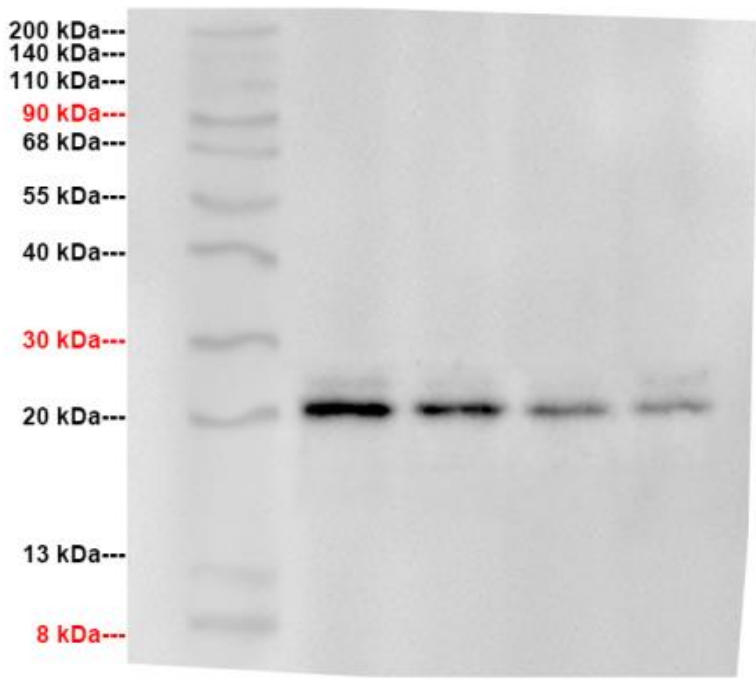

Figure-7(13)-D-GAPDH

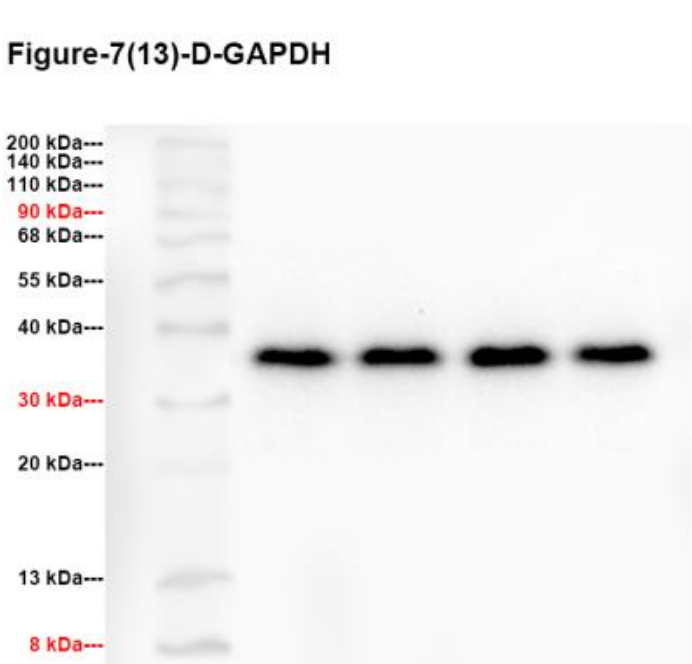

Figure-7(13)-D-GPX4

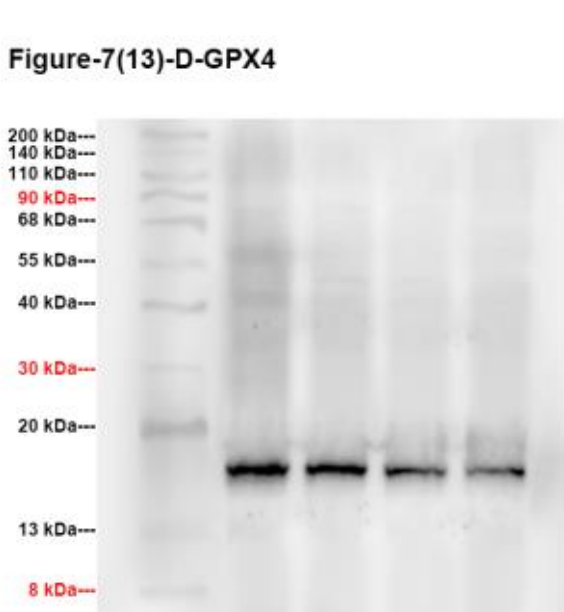

Figure-7(13)-K-ACSL4

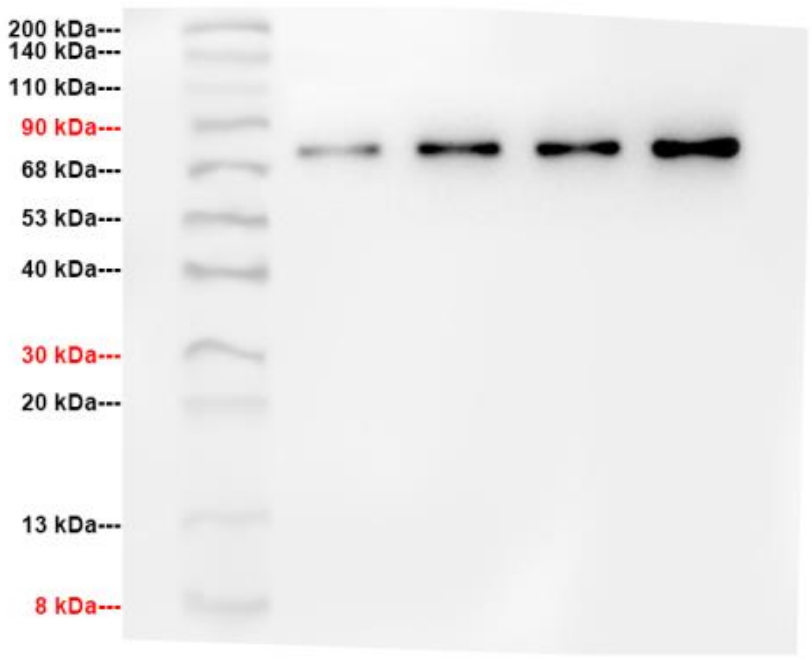

Figure-7(13)-K-FTH1

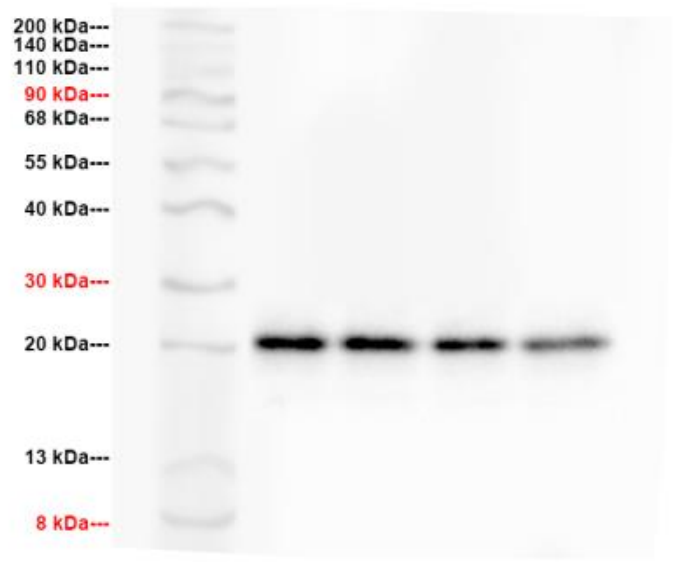

Figure-7(13)-K-GAPDH

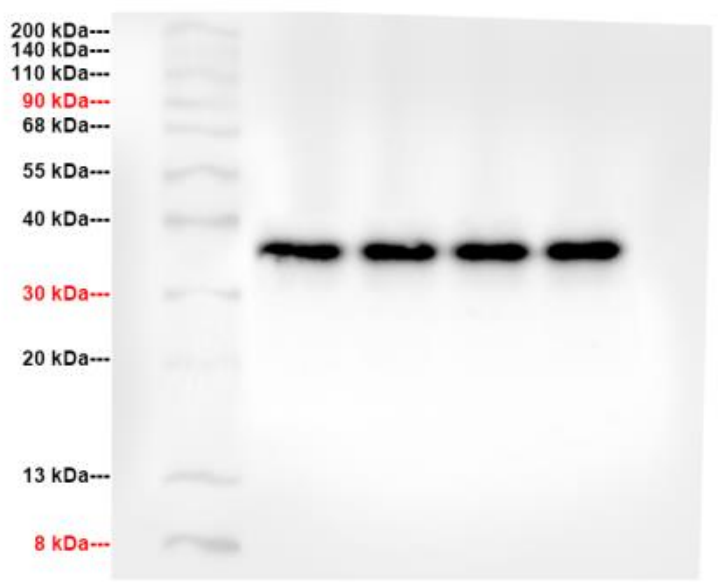

Figure-7(13)-K-GPX4

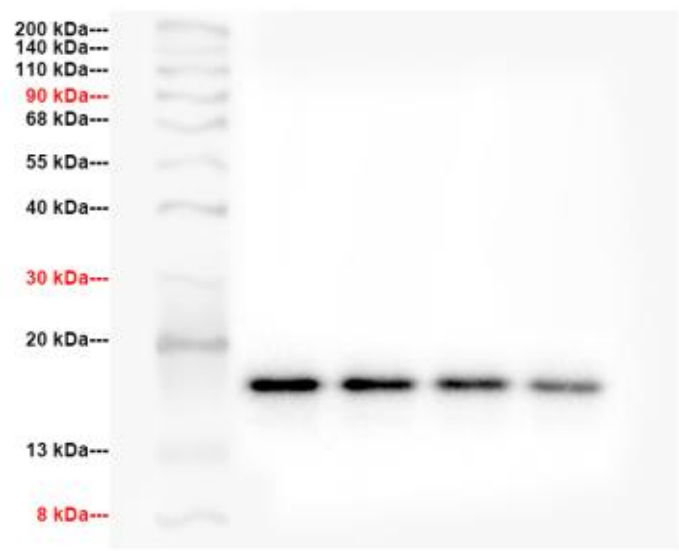

Figure S1(16)-F-Hep3B - N-cadherin

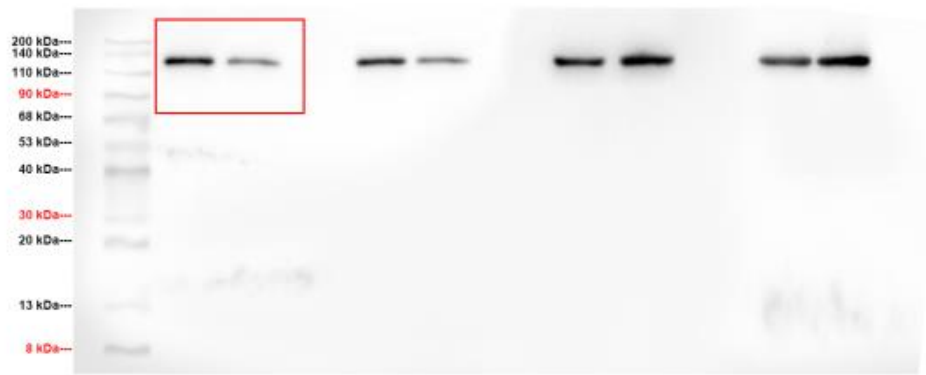

Figure S1(16)-F-Hep3B - E-cadherin

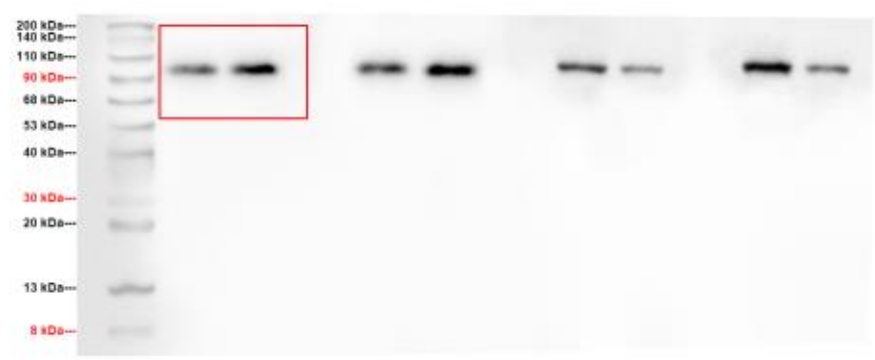

Figure S1(16)-F-Hep3B - GAPDH

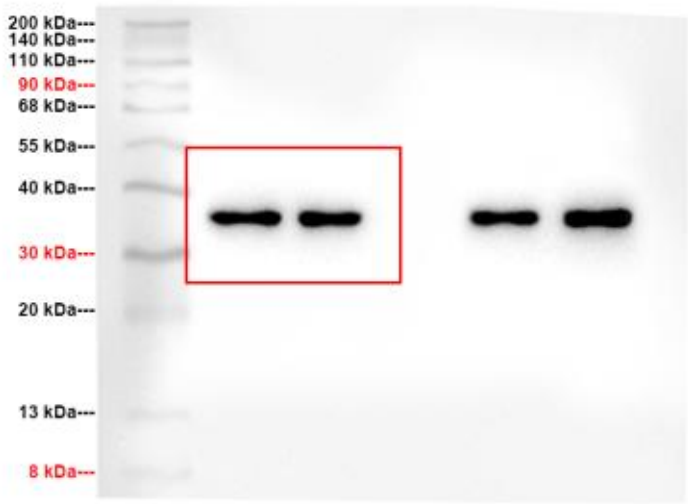

Figure S1(16)-F-Hep3B-Vimentin

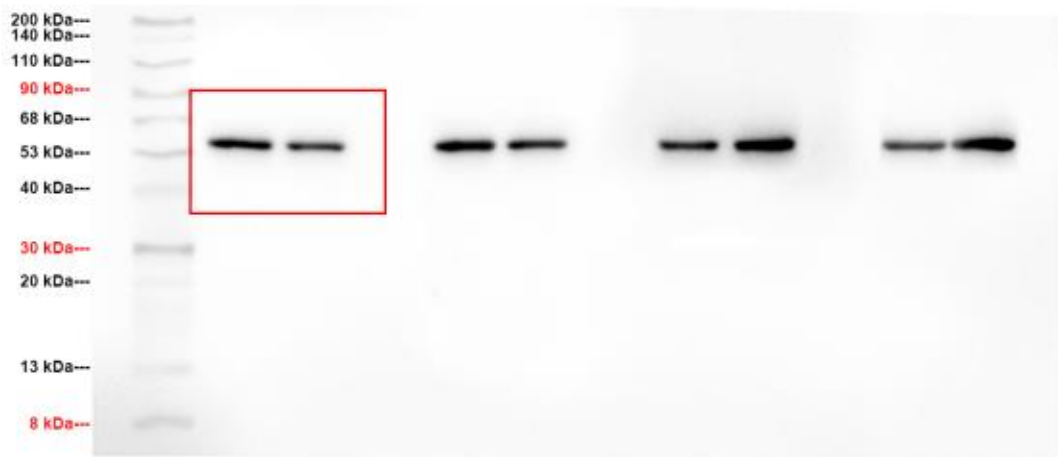

Figure S1(16)-F-MHCC97H - E-cadherin

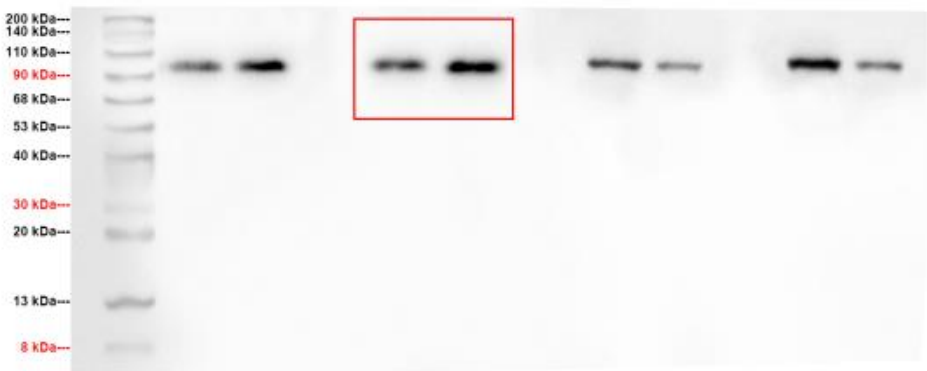

Figure S1(16)-F-MHCC97H - GAPDH

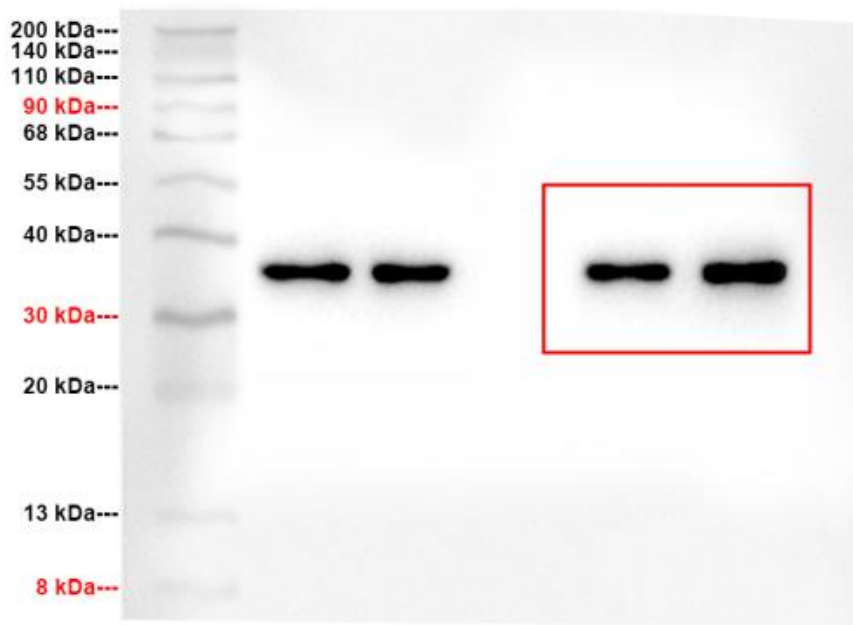

Figure S1(16)-F-MHCC97H - N-cadherin

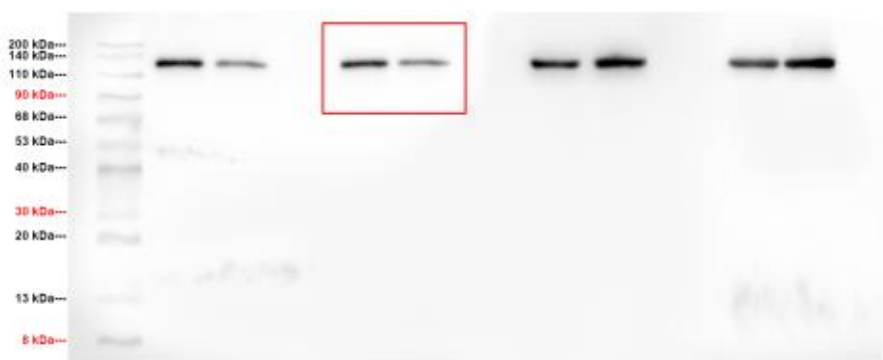

Figure S1(16)-F-MHCC97H-Vimentin

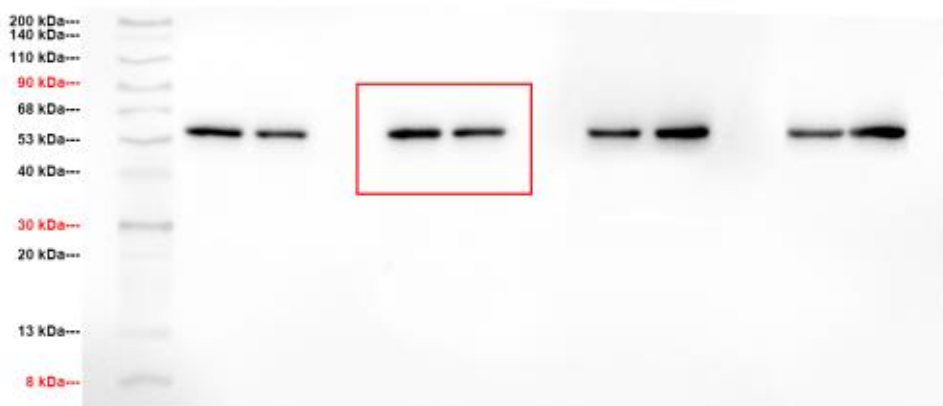

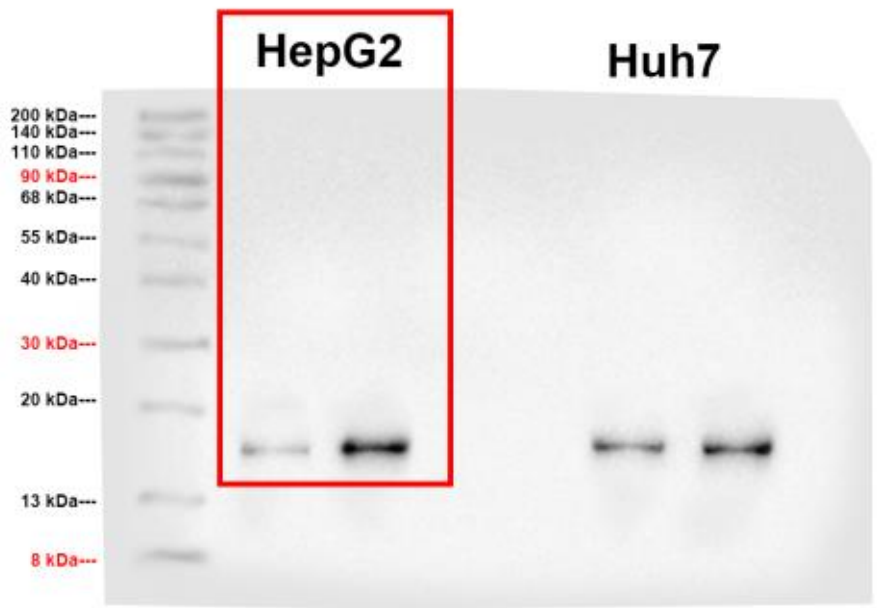
HepG2
Huh7
200 kDa
140 kDa
110 kDa
90 kDa
68 kDa
55 kDa
40 kDa
30 kDa
20 kDa
13 kDa
8 kDa

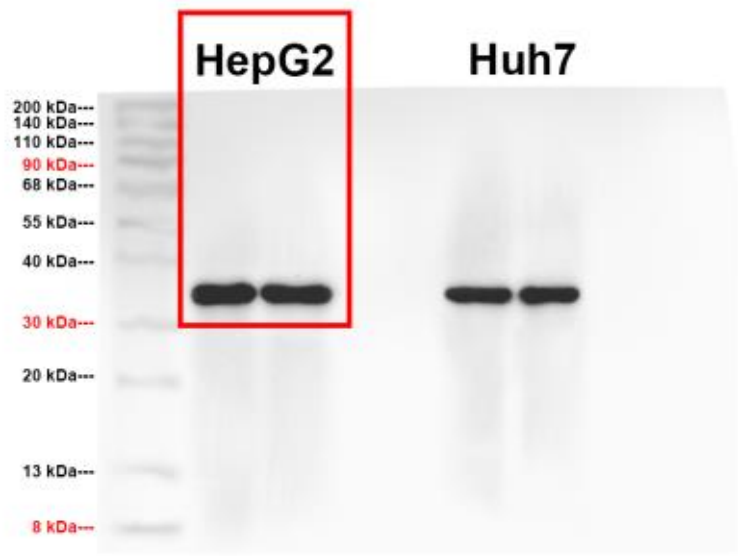
HepG2
Huh7
200 kDa
140 kDa
110 kDa
90 kDa
68 kDa
55 kDa
40 kDa
30 kDa
20 kDa
13 kDa
8 kDa

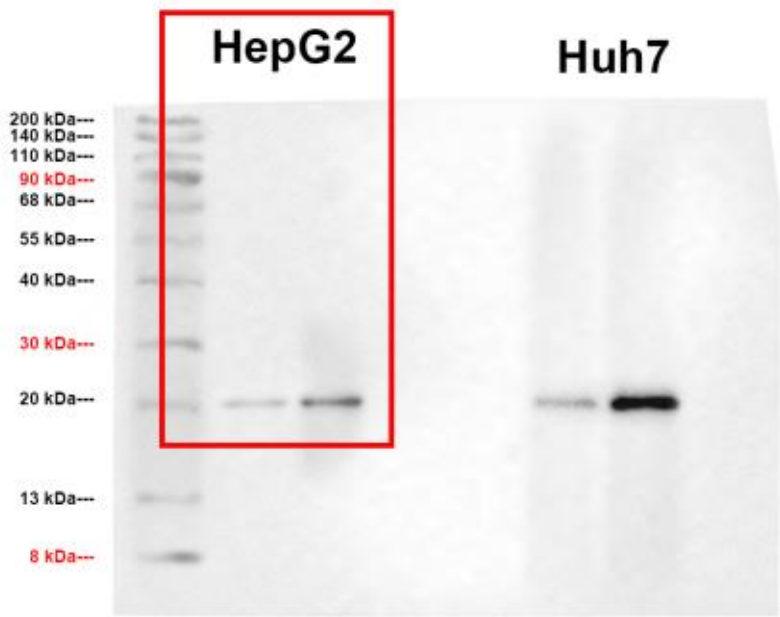
HepG2
Huh7
200 kDa
140 kDa
110 kDa
90 kDa
68 kDa
55 kDa
40 kDa
30 kDa
20 kDa
13 kDa
8 kDa

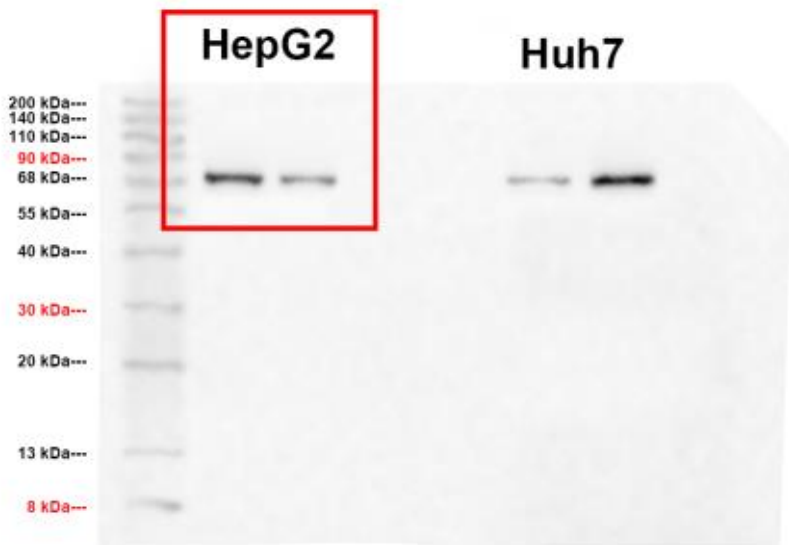
HepG2
Huh7
200 kDa
140 kDa
110 kDa
90 kDa
68 kDa
55 kDa
40 kDa
30 kDa
20 kDa
13 kDa
8 kDa

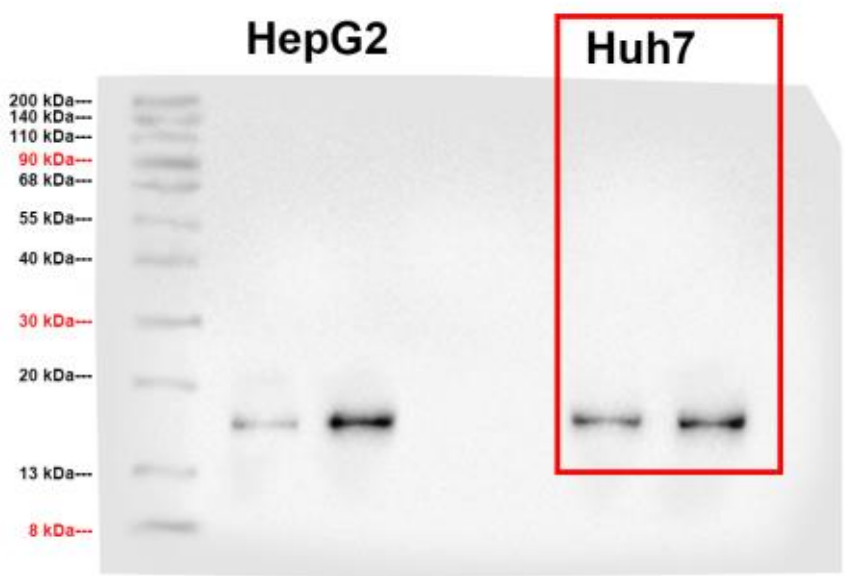
HepG2
Huh7
200 kDa
140 kDa
110 kDa
90 kDa
68 kDa
55 kDa
40 kDa
30 kDa
20 kDa
13 kDa
8 kDa

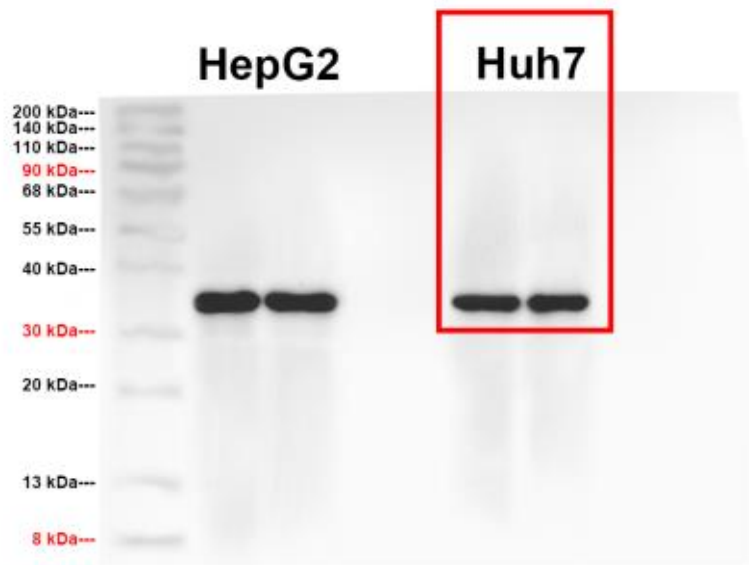
HepG2
Huh7
200 kDa
140 kDa
110 kDa
90 kDa
68 kDa
55 kDa
40 kDa
30 kDa
20 kDa
13 kDa
8 kDa

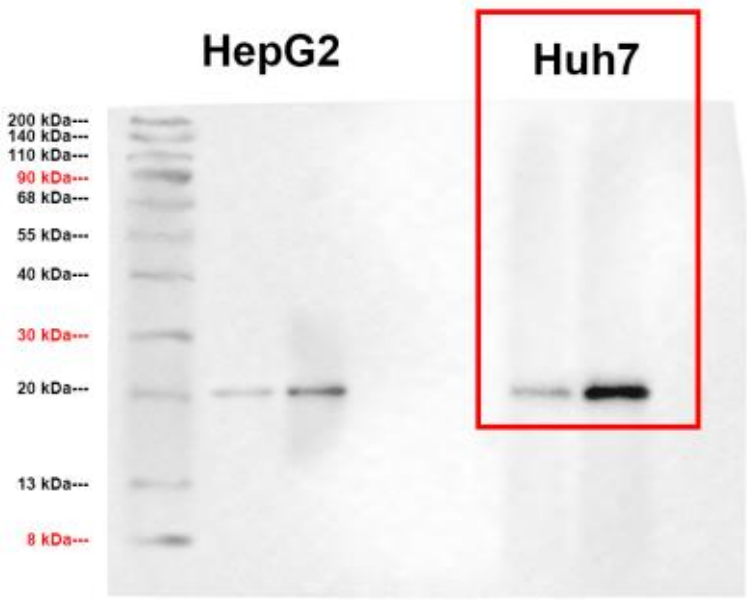

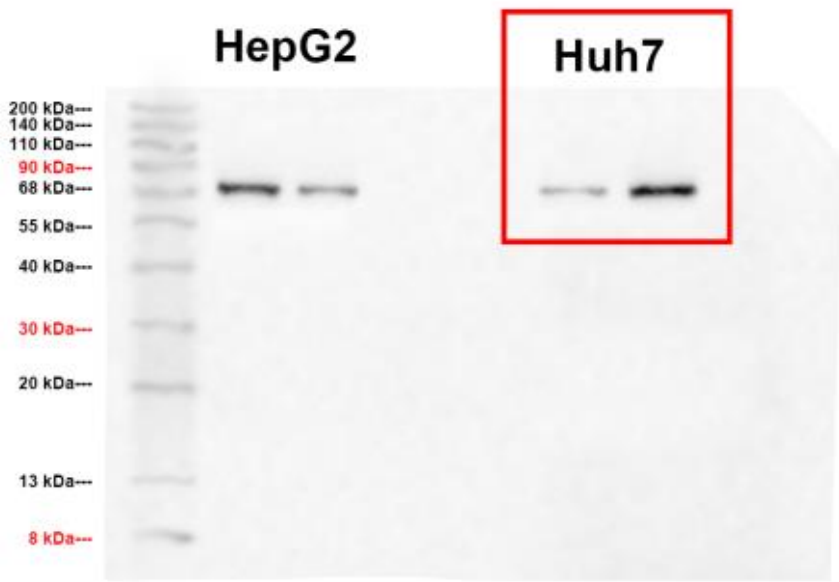

Figure S2(12)-H-GPX4

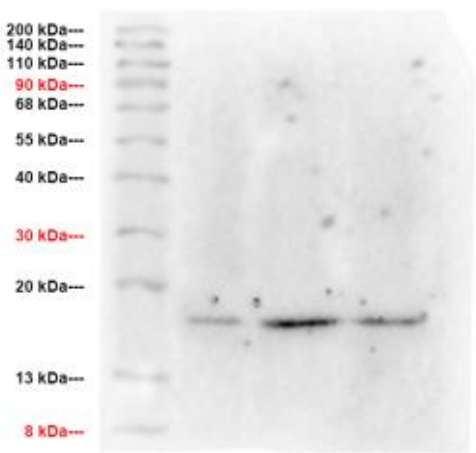

Figure S2(12)-H-GAPDH

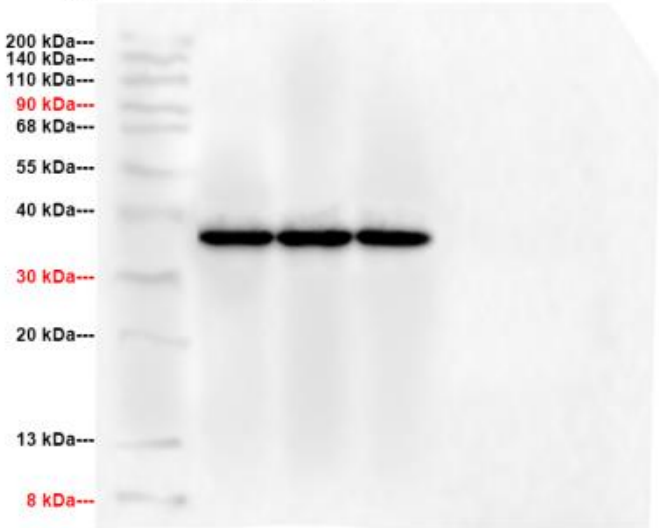

Figure S2(12)-H-FTH1

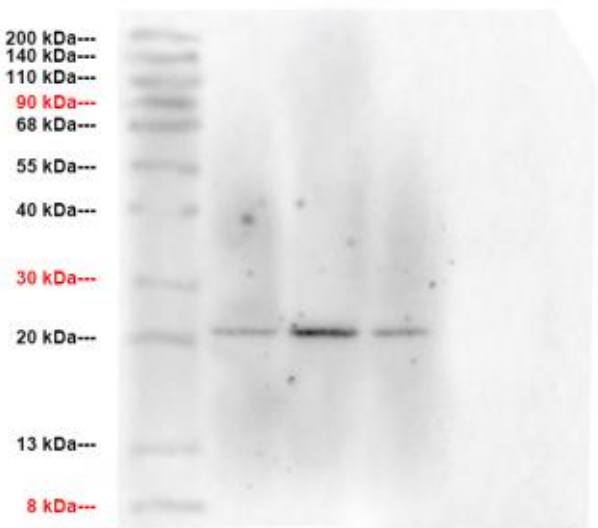

Figure S2(12)-H-ACSL4

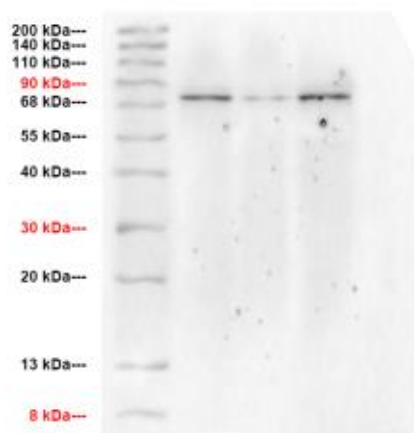

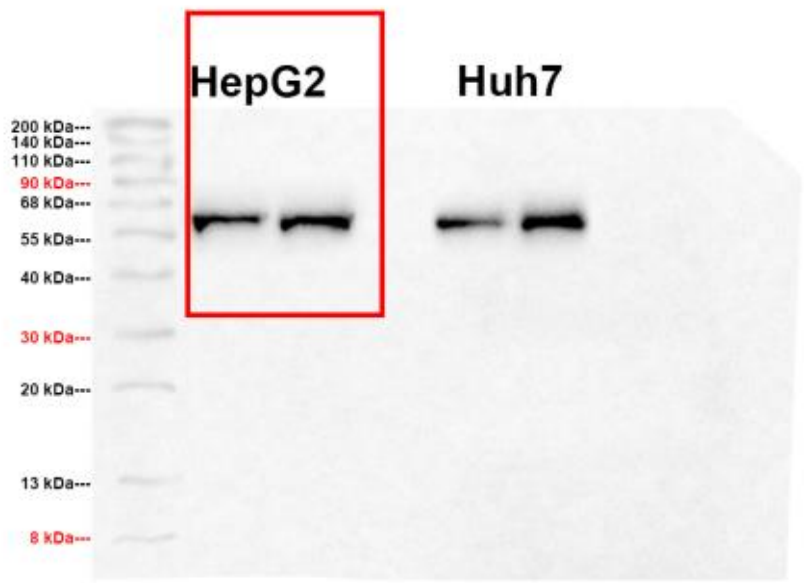
HepG2
Huh7
200 kDa
140 kDa
110 kDa
90 kDa
68 kDa
55 kDa
40 kDa
30 kDa
20 kDa
13 kDa
8 kDa

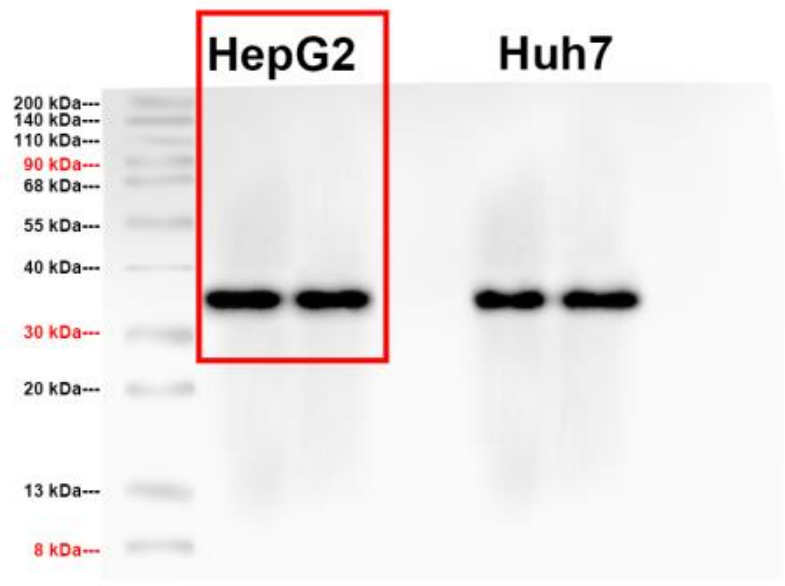
HepG2
Huh7
200 kDa
140 kDa
110 kDa
90 kDa
68 kDa
55 kDa
40 kDa
30 kDa
20 kDa
13 kDa
8 kDa

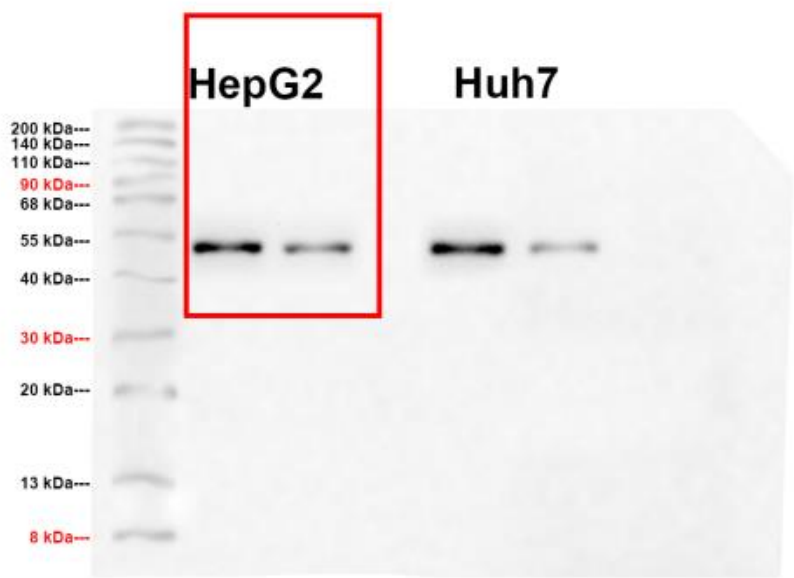
HepG2
Huh7
200 kDa
140 kDa
110 kDa
90 kDa
68 kDa
55 kDa
40 kDa
30 kDa
20 kDa
13 kDa
8 kDa

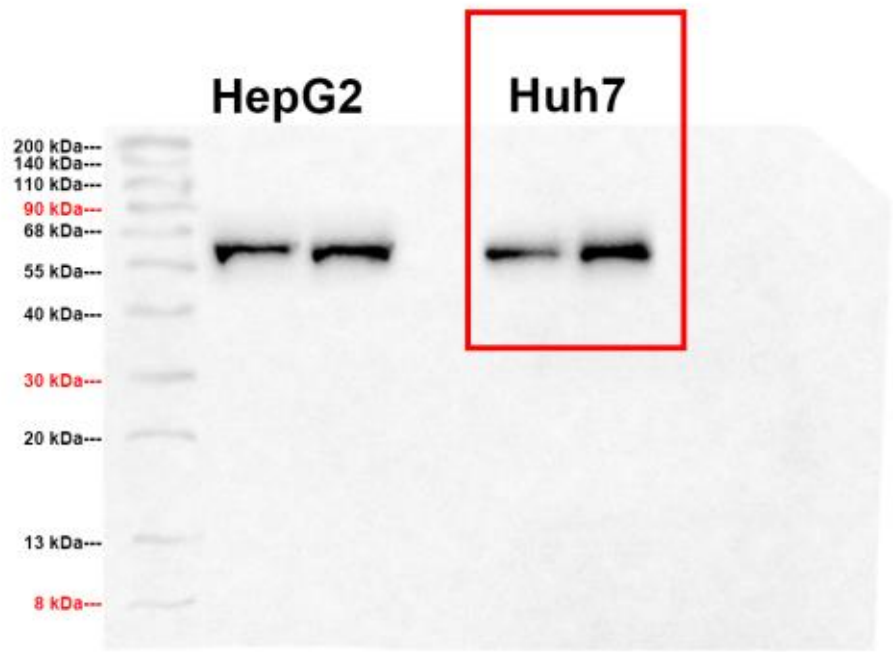
HepG2
Huh7
200 kDa
140 kDa
110 kDa
90 kDa
68 kDa
55 kDa
40 kDa
30 kDa
20 kDa
13 kDa
8 kDa

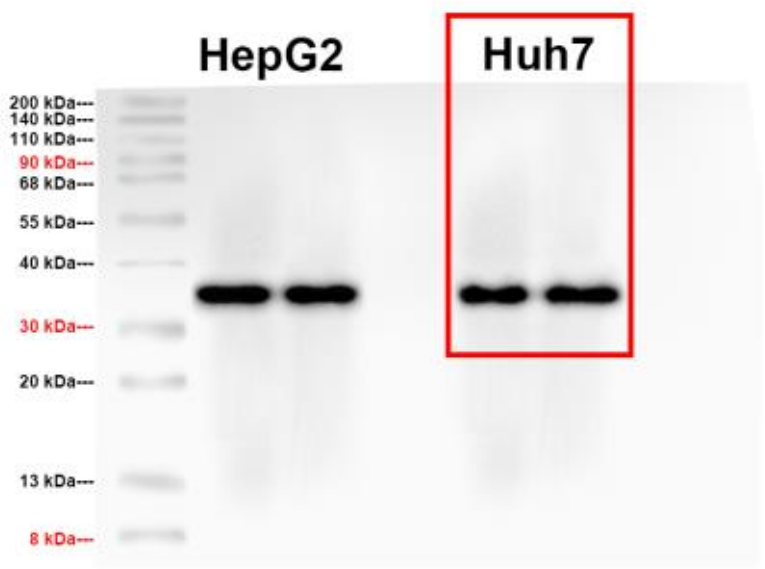
HepG2
Huh7
200 kDa
140 kDa
110 kDa
90 kDa
68 kDa
55 kDa
40 kDa
30 kDa
20 kDa
13 kDa
8 kDa

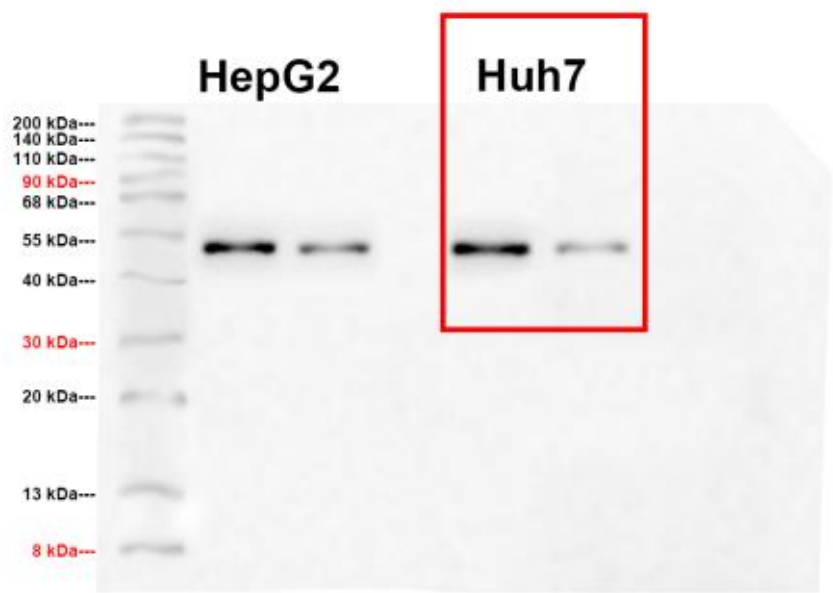
HepG2
Huh7
200 kDa
140 kDa
110 kDa
90 kDa
68 kDa
55 kDa
40 kDa
30 kDa
20 kDa
13 kDa
8 kDa

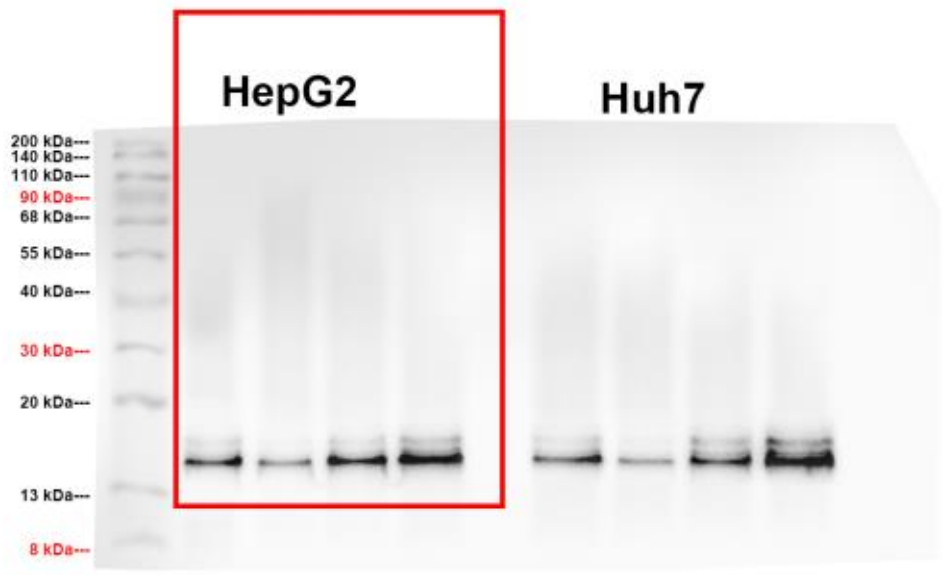
HepG2
Huh7
200 kDa
140 kDa
110 kDa
90 kDa
68 kDa
55 kDa
40 kDa
30 kDa
20 kDa
13 kDa
8 kDa

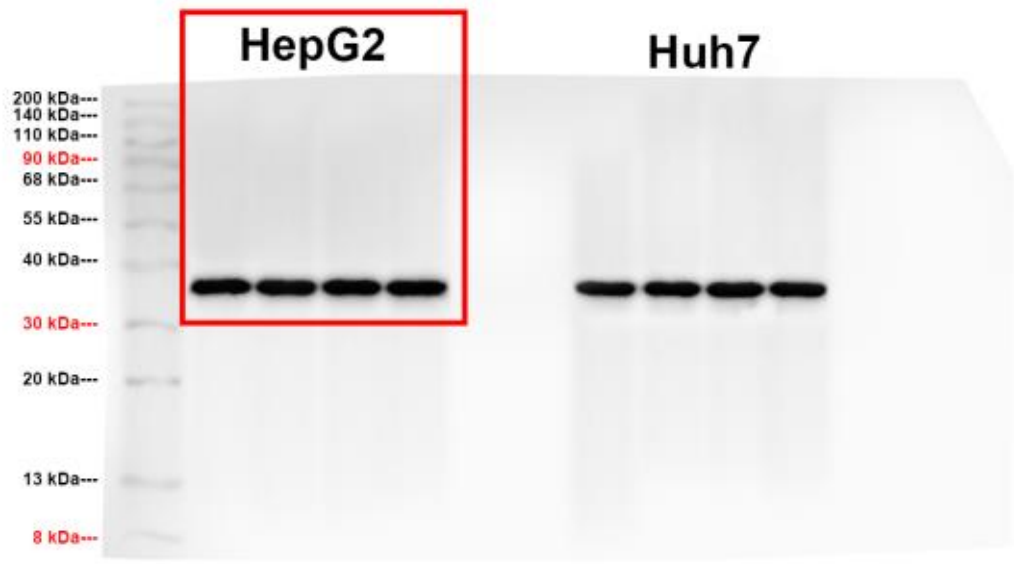
HepG2
Huh7
200 kDa
140 kDa
110 kDa
90 kDa
68 kDa
55 kDa
40 kDa
30 kDa
20 kDa
13 kDa
8 kDa

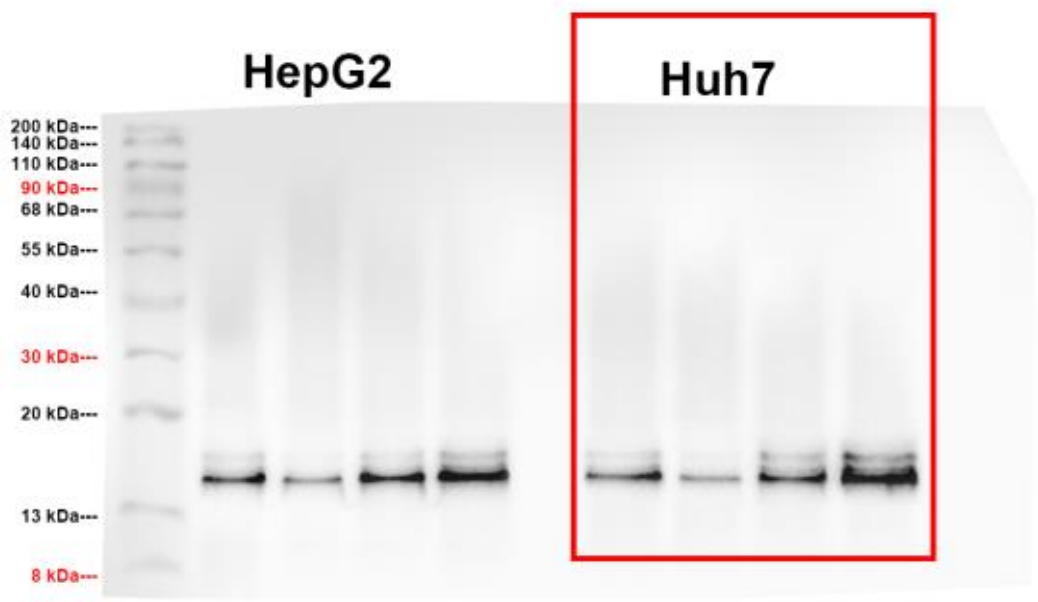
HepG2
Huh7
200 kDa
140 kDa
110 kDa
90 kDa
68 kDa
55 kDa
40 kDa
30 kDa
20 kDa
13 kDa
8 kDa

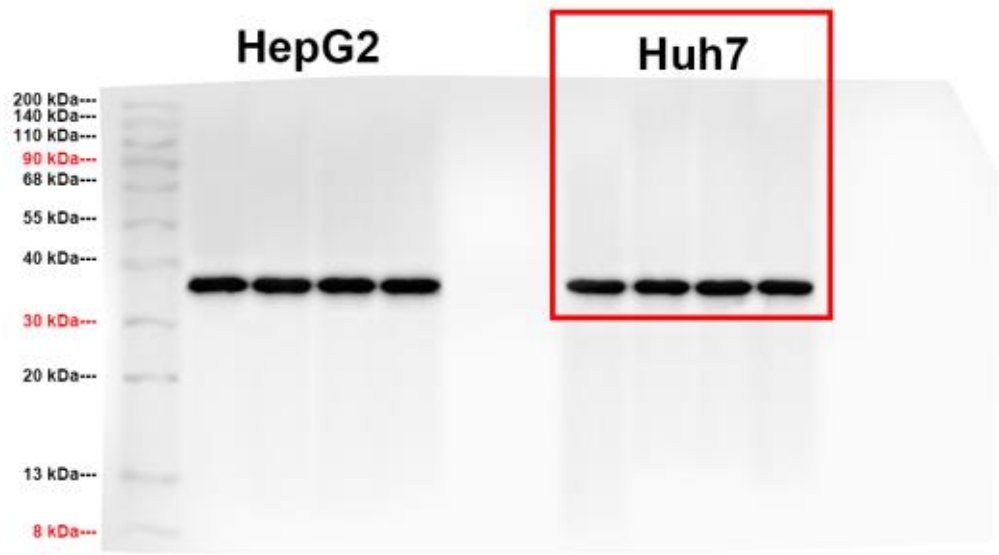
HepG2
Huh7
200 kDa
140 kDa
110 kDa
90 kDa
68 kDa
55 kDa
40 kDa
30 kDa
20 kDa
13 kDa
8 kDa

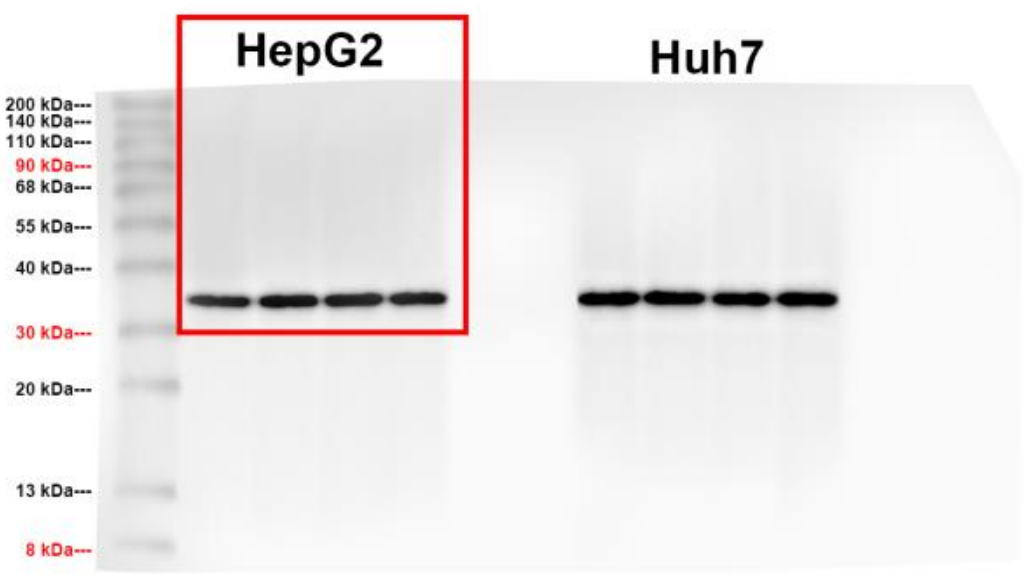
HepG2
Huh7
200 kDa
140 kDa
110 kDa
90 kDa
68 kDa
55 kDa
40 kDa
30 kDa
20 kDa
13 kDa
8 kDa

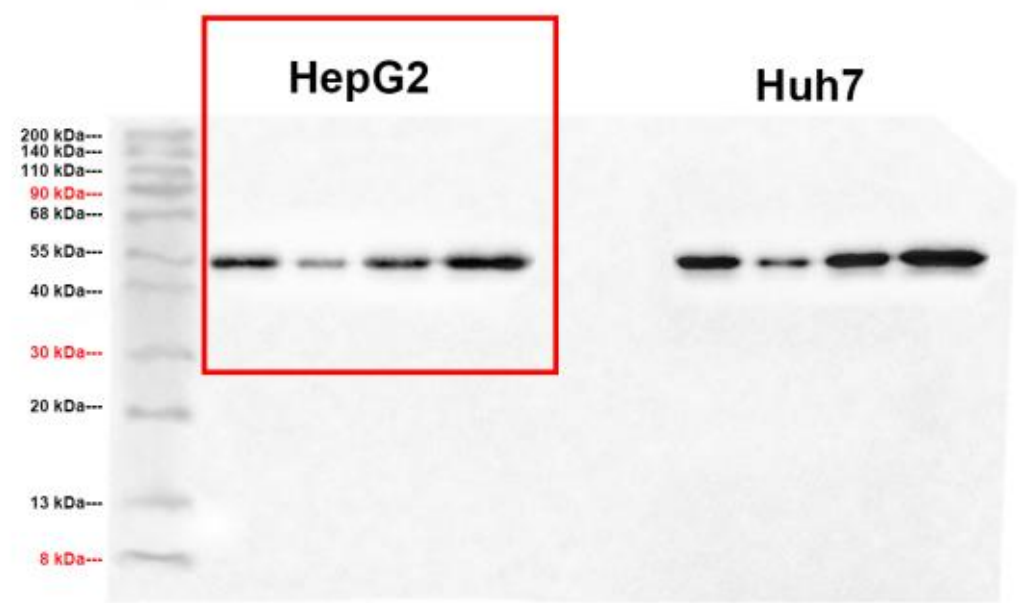
HepG2
Huh7
200 kDa
140 kDa
110 kDa
90 kDa
68 kDa
55 kDa
40 kDa
30 kDa
20 kDa
13 kDa
8 kDa

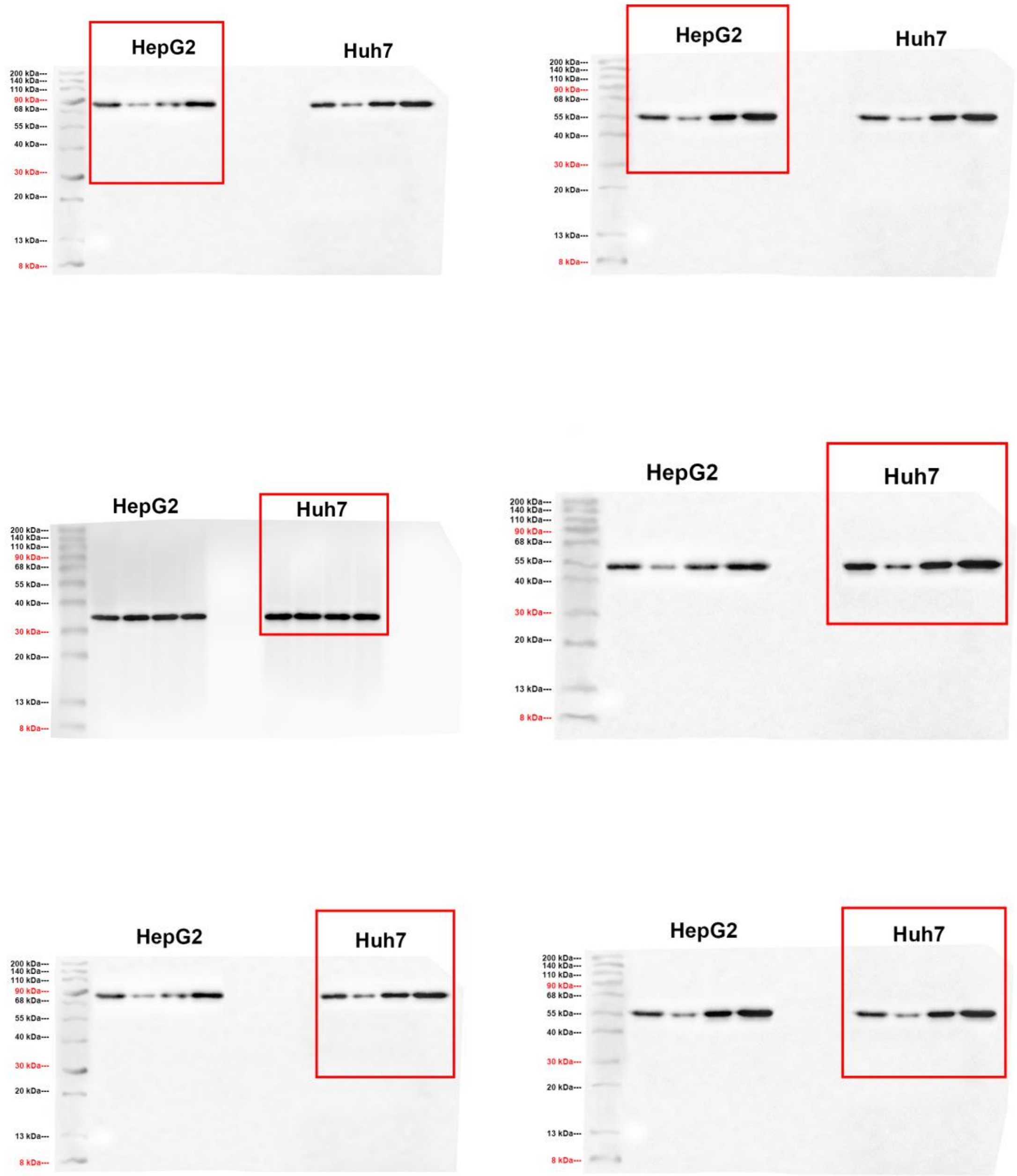
HepG2
Huh7
200 kDa
140 kDa
110 kDa
90 kDa
68 kDa
55 kDa
40 kDa
30 kDa
20 kDa
13 kDa
8 kDa

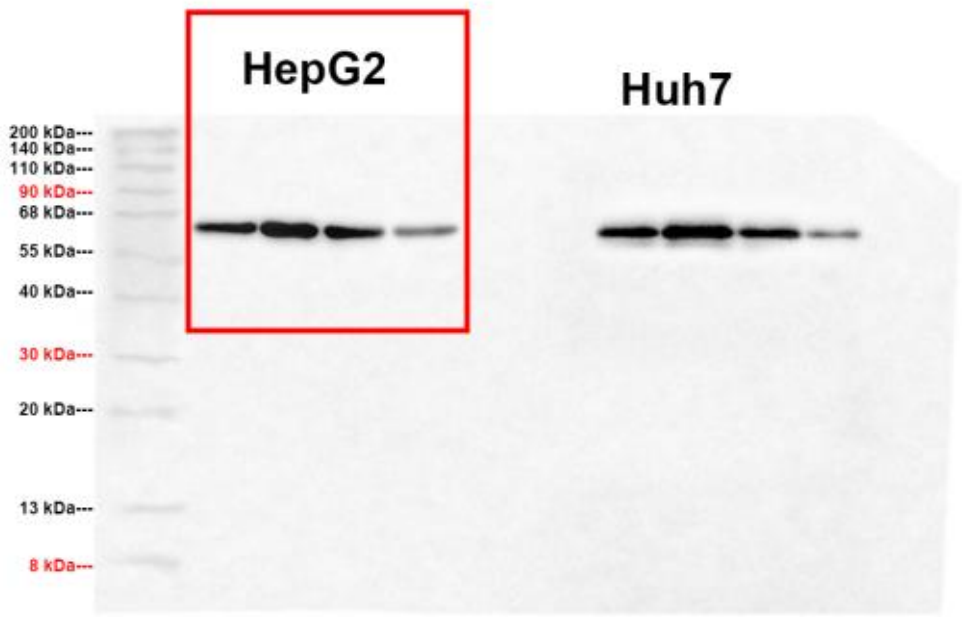
HepG2
Huh7
200 kDa
140 kDa
110 kDa
90 kDa
68 kDa
55 kDa
40 kDa
30 kDa
20 kDa
13 kDa
8 kDa

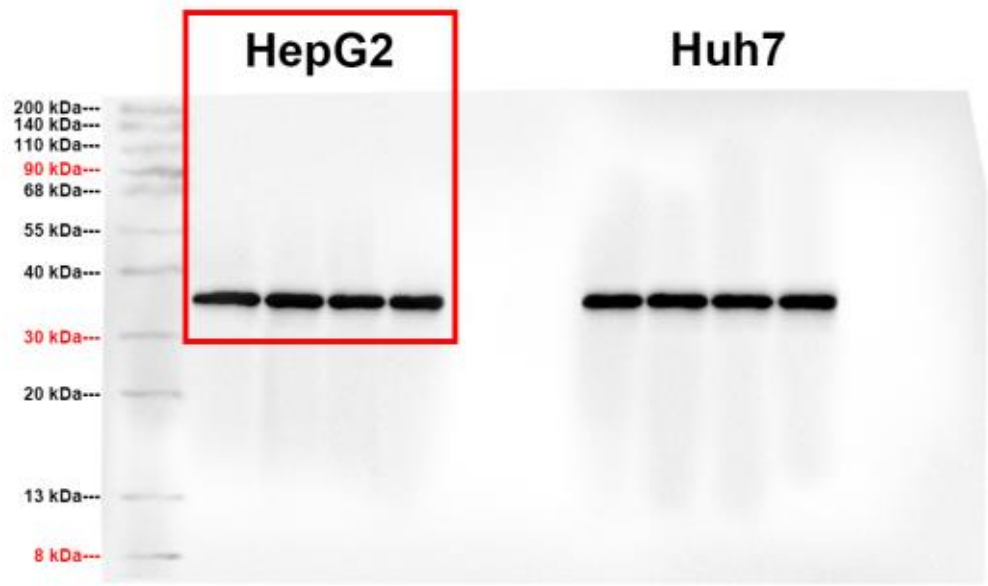
HepG2
Huh7
200 kDa
140 kDa
110 kDa
90 kDa
68 kDa
55 kDa
40 kDa
30 kDa
20 kDa
13 kDa
8 kDa

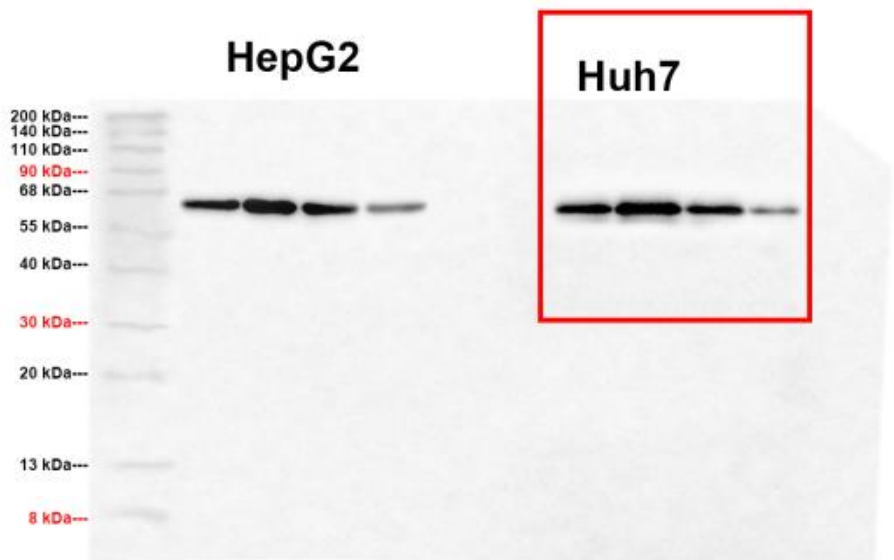
HepG2
Huh7
200 kDa
140 kDa
110 kDa
90 kDa
68 kDa
55 kDa
40 kDa
30 kDa
20 kDa
13 kDa
8 kDa

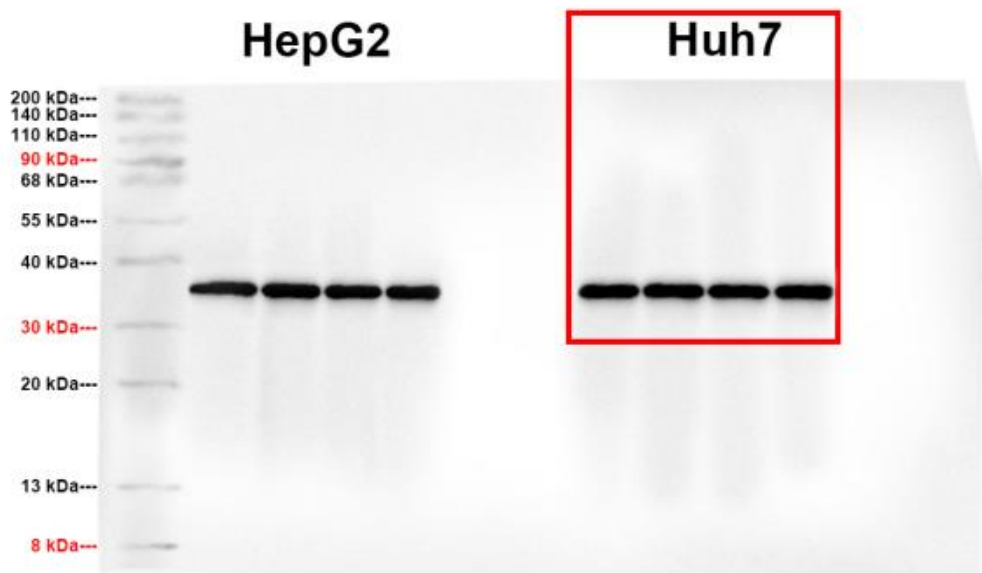
HepG2
Huh7
200 kDa
140 kDa
110 kDa
90 kDa
68 kDa
55 kDa
40 kDa
30 kDa
20 kDa
13 kDa
8 kDa
