# Supplementary material for: OTUB1 inhibits autophagy-dependent ferroptosis in hepatocellular carcinoma by stabilizing p62 via non-canonical deubiquitination
Source: Cell Death Discov. 2026 Jul 22;12:382. doi: 10.1038/s41420-026-03241-5 (PMC13601636; doi:10.1038/s41420-026-03241-5)
Supplement: Supplementary file 2 [file 41420_2026_3241_MOESM1_ESM.docx]

**OTUB1 inhibits autophagy-dependent ferroptosis in hepatocellular carcinoma by stabilizing p62 via non-canonical deubiquitination**

**Supplementary Materials and Methods**

**Bioinformatics**

Transcriptomic profiles and clinicopathological parameters from hepatocellular carcinoma cases were curated via The Cancer Genome Atlas repository (access portal: https://portal.gdc.cancer.gov/). Gene expression microarray data for the GSE87630 cohort were additionally acquired through the NCBI's Gene Expression Omnibus platform. Subsequent bioinformatic interrogation of OTUB1 transcriptional levels employed the R computational environment (v4.2.2).

**Antibodies and reagents**

Pharmacological agents including proteasome inhibitor MG132 (#HY-13259), protein synthesis blocker cycloheximide (CHX) (#HY-12320), OTUB1 inhibitor OTUB1/USP8-IN-1 (#HY-151563), multikinase inhibitor Lenvatinib (#HY-10981), ferroptosis inhibitor Ferrostatin-1 (Fer-1) (#HY-100579), and ferroptosis inducer Erastin (#HY-15763) were procured from MedChemExpress (Monmouth Junction, NJ, USA). DMSO (Sigma #D2560) served as solvent control. Immunoreactions employed the following primary antibodies: OTUB1 (1:1000, Abcam #ab270959); EMT markers: E-cadherin (1:5000, Proteintech #20874-1-AP), N-cadherin (1:5000, Proteintech #22018-1-AP), Vimentin (1:1000, Santa Cruz Biotechnology #sc-32322); Ferroptosis regulators: ACSL4 (1:1000, Abcam #ab155282), FTH1 (1:2000, Abcam #ab65080), GPX4 (1:5000, Abcam #ab125066); Autophagy machinery: ATG5 (1:1000, HUABIO #E1A6301), p62 (1:2000, HUABIO #ER1806-26), LC3B (1:2000, HUABIO #M191-3), ATG7 (1:2000, HUABIO #R1307-3), Beclin1 (1:2000, Proteintech #11306-1-AP); Ubiquitin (1:1000, CST #3936S); Epitope tags: Flag-Tag (1:2000, CST #14793S), Myc-Tag (1:2000, CST #2278S), HA-Tag (1:2000, CST #3724S); Controls: GAPDH (1:2000, Servicebio #GB11002), IgG (Proteintech #B900610); Ki-67 (1:200, Servicebio #GB111141); Apoptosis markers: Bax (1:1000, Proteintech #50599-2-Ig), Bcl-2 (1:2000, Proteintech #12789-1-AP), cleaved-Caspase3 (1:1000, Proteintech #19677-1-AP)

**Western blotting analysis**

Protein lysates were extracted from samples using ice-cold RIPA Buffer (Servicebio), which contained protease and phosphatase inhibitors (Beyotime). Quantified protein extracts underwent electrophoretic separation via SDS-polyacrylamide gels followed by blotting onto PVDF membranes (Millipore). After blocking with 5% skim milk, membranes were incubated with primary antibodies at 4 ℃ overnight, then followed by HRP-conjugated goat anti-rabbit/mouse secondary antibodies (1:5000; Abclonal #AS014/AS003) at room temperature for 1 hour. Immunocomplexes were ultimately visualized by enhanced chemiluminescence detection on a ChemiDoc MP Imaging System.

**CCK8 assay and colony formation assay**

Cell viability was assessed using a CCK-8 assay kit (#HY-K0301, MedChemExpress). For colony formation quantification, 1,000 cells were plated and cultured for 14 days, followed by fixation in 4% paraformaldehyde (30 min) and staining with 0.5% crystal violet (60 min). Colonies were imaged and counted manually.

**Transwell assays**

Cell migration capacity was assessed using 8 µm-pore Transwell chambers. Serum-starved HCC cells (1×10^5^ in 200 µL FBS-free medium) were seeded in apical chambers, with 20% FBS-containing medium serving as chemoattractant in basal chambers. Following 24 h incubation, migrated cells adherent to the membrane underside were fixed in 4% paraformaldehyde (15 min) and stained with 1% crystal violet (20 min) for microscopic analysis.

**CFDA-SE staining**

Proliferation dynamics were tracked with CFDA-SE (5 μM, 37 ℃, 10 min). After labeling, cells were washed three times with PBS washes and then imaged (488 nm ex / 518 nm em). Division peaks were quantified using FlowJo v10.8 software (CFSE dilution algorithm).

**Protein half-life assay**

P62 protein stability was assessed by CHX-chase analysis. Transfected cells in the exponential growth phase underwent 10 μg/mL cycloheximide treatment for specified durations (0, 0.5, 1, 2 h), with subsequent lysis and immunoblotting. Degradation kinetics were modeled using first-order decay curves based on time-dependent band intensity quantification relative to loading controls.

**Cell apoptosis analysis**

HCC cells grown to 80% confluence in 6-well plates underwent serum starvation (24 h) prior to collection. Apoptosis was quantified using Annexin V-FITC/PI dual staining (Apoptosis Kit #LM-AP101-100, Lianke Bio) with analyzed immediately on CytoFlex LX (Beckman, Shanghai). Data processing employed FlowJo v10.6.0.

**Immunohistochemical and hematoxylin** **& eosin staining**

MHCC97H xenografts underwent 4% paraformaldehyde fixation and paraffin embedding, with subsequent sectioning at 4 µm thickness for Immunohistochemical (IHC)/ hematoxylin & eosin (H&E) analyses. Tissue sections were deparaffinized, subjected to heat-induced epitope retrieval in 10 mM sodium citrate (pH 6.0, 95 ℃, 20 min), and quenched with 3% H_2_O_2_ (25 min, RT). After blocking in 3% BSA (30 min), primary antibodies (Ki67, Servicebio #GB111141) were applied overnight at 4 ℃. HRP-conjugated secondary antibodies were incubated (60 min, RT), followed by DAB chromogenic development and hematoxylin counterstaining. Brightfield imaging employed a Nikon Ni-E microscope.

**Assessment of lipid peroxidation, GSH, and iron content**

Intracellular ferrous iron (Fe^2+^), reduced glutathione (GSH), and malondialdehyde (MDA) levels were quantified using commercial kits: Fe^2+^ (Elabscience #E-BC-K773), GSH (Jiancheng Bioengineering #A006-2-1), MDA (Beyotime #S0131S). Cell lysates prepared with either Beyotime lysis buffer or 0.1 M PBS were processed per manufacturer protocols. Absorbance readings utilized a SpectraMax i3x microplate reader: GSH at 405 nm and MDA at 532 nm. Data normalized to total protein concentration determined by BCA assay.

**Mito-tracker red staining**

MitoTracker^TM^ Red CMXRos (150 nM, 37 ℃) stained live cells (3×10^5^/well in 6-well plates) for 20 min under experimental conditions. After triple PBS washes, mitochondrial networks were imaged at consistent laser intensity via confocal microscopy (TRITC channel: 579/599 nm ex/em), enabling quantitative assessment of fission/fusion dynamics.

**Lipid Peroxidation Quantification via BODIPY 581/591 Probe**

Cellular lipid peroxidation was quantified using C11-BODIPY 581/591 (2.5 μM; Thermo #D3861). Post-treatment cells from 6-well culture plates were trypsinized, PBS-rinsed, and incubated with probe (37 ℃, 20 min). After triple PBS washes, resuspended samples underwent flow cytometry detecting emission shift ratio (590 nm to 510 nm) corresponding to peroxidation intensity. FlowJo-processed data analyzed using standardized gating strategies for quantitation.

**Supplementary Figures**

**Supplementary Fig. S1. OTUB1 is upregulated in HCC and associated with poor prognosis.**

(A, B) OTUB1 mRNA expression in TCGA (A) and GEO GSE87630 (B) datasets. (C, F) Kaplan-Meier analysis of overall survival (C) and disease-free survival (F) in HCC patients with high vs. low OTUB1 expression. (D) Protein quantitative analysis of Fig. 1B (n = 3). (E) IHC from the Human Protein Atlas. Scale bars: 100 μm. (F, G) OTUB1 expression in HCC cell lines (HepG2, Huh7, Hep3B, MHCC97H) versus normal LX-2 cells assessed by RT-qPCR (F) and Western blot (G) (n = 3). (H, I) Validation of OTUB1 knockdown (H) and overexpression (I) in indicated cell lines by Western blot (n = 3). **, *P* < 0.01; ***, *P* < 0.001; ****, *P* <0.0001.

**Supplementary Fig. S2. OTUB1 promotes HCC cell proliferation, migration, and EMT *in vitro*.** (A) The proliferation of Hep3B and MHCC97H cells was assessed using colony formation assays (n = 3). (B) CFDA staining was performed to assess cell viability in the indicated Hep3B and MHCC97H cells (n = 3). Scale bar: 100 μm. (C) Annexin V/PI staining and flow cytometry were performed to assess the cell death rate in the indicated Hep3B and MHCC97H cells (n = 3). (D) Migration abilities of Hep3B and MHCC97H cells were evaluated with Transwell assays (n = 3). Scale bar: 100 μm. (E) Cell adhesion abilities of the indicated Hep3B and MHCC97H cells were evaluated using Calcein AM staining (n = 3). Scale bar: 100 μm. (F) The expression of EMT markers in the indicated Hep3B and MHCC97H cells was measured by Western blot (n = 3). (G) The proliferation of HepG2 and Huh7 cells was assessed using colony formation assays (n = 3). (H) CFDA staining was performed to assess cell viability in the indicated HepG2 and Huh7 cells (n = 3). Scale bar: 100 μm. (I) Annexin V/PI staining and flow cytometry were performed to assess the cell death rate in the indicated HepG2 and Huh7 cells (n = 3). (J) Migration abilities of HepG2 and Huh7 cells were evaluated with Transwell assays. The migration rate was calculated (n = 3). Scale bar: 100 μm. (K) Cell adhesion abilities of the indicated HepG2 and Huh7 cells were evaluated using Calcein AM staining (n = 3). Scale bar: 100 μm. (L) The expression of EMT markers in the indicated HepG2 and Huh7 cells was measured by Western blot (n = 3). *, *P* < 0.05; **, *P* < 0.01; ***, *P* < 0.001.

**Supplementary Fig. S3. Erastin reverses the inhibition of ferroptosis induced by OTUB1 overexpression.** (A) Quantitative analysis of lipid ROS in Figure 1G (n = 3). (B) The relative values of GSH, MDA, and Fe^2+^ concentrations in the indicated HepG2 and Huh7 cells were determined (n = 3). (C) Representative fluorescence microscopy images of the indicated HepG2 and Huh7 cells stained with BODIPY 581/591 C11 (n = 3). Scale bar: 100 μm. (D, E) Western blot and quantitative analysis was used to detect the expression of ferroptosis-associated proteins in the indicated HepG2 and Huh7 cells (n = 3). (F) Immunofluorescence detection of mitochondrial membrane potential (stained with MitoTracker Red) in the indicated HepG2 and Huh7 cells (n = 3). Scale bar: 100 μm. (G) CCK-8 assays were conducted to determine the viability of indicated HepG2 and Huh7 cells following treatment with Erastin (2-10 μM) (n = 3). (H) Quantitative analysis of lipid ROS in Fig. 1M (n = 3). (I) Western blot analysis was used to detect the expression of ferroptosis-associated proteins in the indicated Huh7 cells (n = 3). (J) The relative values of GSH, MDA, and Fe^2+^ concentrations in the indicated Huh7 cells were determined (n = 3). (K) Representative fluorescence microscopy images of the indicated Huh7 cells stained with BODIPY 581/591 C11 (n = 3). Scale bar: 100 μm. (L) Immunofluorescence detection of mitochondrial membrane potential (stained with MitoTracker Red) in the indicated Huh7 cells (n = 3). Scale bar: 100 μm. (M) The proliferation of Huh7 cells was assessed using colony formation assays (n = 3). (N) Migration abilities of Huh7 cells were evaluated with Transwell assays (n = 3). Scale bar: 100 μm. *, *P* < 0.05; **, *P* < 0.01; ***, *P* < 0.001.

**Supplementary Fig. S4. OTUB1 suppresses autophagic flux post-translationally, which can be rescued by Erastin-induced cellular stress.** (A) Western blot analysis was used to detect the expression of autophagy-associated proteins (p62, and Beclin 1) in HepG2 and Huh7 cells transfected with Vector or OTUB1 constructs (n = 3). (B) Representative confocal microscopy images of mRFP-GFP-LC3 fluorescence in HepG2 and Huh7 cells transfected with Vector or OTUB1 constructs and infected with mRFP-GFP-LC3 adenovirus (n = 3). Scale bar: 10 μm. (C-E) Relative mRNA expression of SQSTM1 (p62) (C), ATG5 (D) and Beclin1 (E) in HepG2 and Huh7 cells transfected with empty vector or OTUB1 overexpression plasmid, detected by qPCR. (F-H) Relative mRNA expression of SQSTM1 (p62) (F), ATG5 (G) and Beclin1 (H) in Hep3B and MHCC97H cells transduced with shCtrl or shOTUB1 lentivirus, detected by qPCR. (I) Protein quantitative analysis of Figure 2N. (J) Western blot analysis was used to determine the expression of LC3-Ⅰ/Ⅱ protein in OTUB1-overexpressing HepG2 and Huh7 cells following treatment with Erastin (n = 3). (K) Western blot analysis was used to detect the expression of autophagy-associated proteins (ATG5, ATG7, and Beclin 1) in OTUB1-overexpressing HepG2 and Huh7 cells following treatment with Erastin (n = 3). (L, N) Western blot and quantitative analysis was used to determine the expression of p62 protein in OTUB1-overexpressing HepG2 and Huh7 cells following treatment with Erastin (n = 3). (M, O) Representative confocal microscopy images of mRFP-GFP-LC3 fluorescence in Huh7 cells: Vector, OTUB1, OTUB1+Erastin, Erastin alone. All groups were transfected with indicated constructs and infected with mRFP-GFP-LC3 adenovirus. The number of autophagosomes (yellow puncta, GFP^+^/mRFP^+^) and autolysosomes (red puncta, GFP^-^/mRFP^+^) per cell were independently quantified to assess the blockade of autophagic flux (n = 3). Scale bar: 10 μm. ns, *P* > 0.05; *, *P* < 0.05; **, *P* < 0.01; ***, *P* < 0.001.

**Supplementary Fig. S5. Autophagic suppression rescues OTUB1 knockdown-induced ferroptosis activation.**

(A-C) Pharmacological inhibition of autophagy with CQ abrogates ferroptosis induced by OTUB1 depletion. Hep3B cells were subjected to shOTUB1 alone or in combination with CQ treatment. (A) Western blot analysis of LC3 and p62. (B) Relative levels of intracellular GSH, MDA, and Fe^2+^ (n = 3). (C) Western blot analysis of ferroptosis-related proteins ACSL4, FTH1, and GPX4. (D-F) Exogenous p62 accumulation counteracts OTUB1 knockdown-induced ferroptosis. MHCC97H cells were co-transfected with shOTUB1 and a p62 overexpression plasmid. (D) Western blot analysis of LC3, Beclin 1, and p62. (E) Relative levels of GSH, MDA, and Fe^2+^ (n = 3). (F) Western blot analysis of ferroptosis markers ACSL4, FTH1, and GPX4. (G-H) Genetic ablation of autophagy core machinery abolishes ferroptotic phenotypes triggered by OTUB1 depletion. MHCC97H cells were co-transfected with shOTUB1 and shATG5. (G) Relative levels of GSH, MDA, and Fe^2+^ (n = 3). (H) Western blot and corresponding quantitative analysis of ferroptosis markers ACSL4, GPX4, and FTH1 under the indicated genetic modifications (n = 3). *, *P* < 0.05; **, *P* < 0.01; ***, *P* < 0.001.

**Supplementary Fig. S6. OTUB1 stabilizes p62 through non-canonical deubiquitination to inhibit autophagy-dependent ferroptosis and drive HCC progression.** (A) Western blot and corresponding quantitative analysis of OTUB1, ATG5, and p62 in MHCC97H cells subjected to the indicated combinations of OTUB1 knockdown (shOTUB1), ATG5 knockdown (shATG5), and proteasome inhibition (MG132) (n=3). (B-C) CHX chase assays were performed to monitor the protein half-life of p62 under the aforementioned conditions. Representative Western blots (C) and the statistical quantification of relative p62 degradation kinetics (B) are shown. (D) Quantitative analysis of ferroptosis markers in Fig. 3K (n = 3). (E) Quantitative analysis of lipid ROS in Fig. 3L (n = 3). (F) Quantitative analysis of mitochondrial red fluorescence in Fig. 3N (n = 3). (G) Quantitative analysis of autophagosomes and autolysosomes in Fig. 3O (n = 3). (H) Quantitative analysis of p62 protein in Fig. 3P (n = 3). (I) Quantitative analysis of ATG7, ATG5, and Beclin1 proteins in Fig. 3Q (n = 3). (J) The clone number was counted using ImageJ in Fig. 3R (n = 3). (K) The migration rate was calculated in Fig. 3S (n = 3). ns, *P* > 0.05; *, *P* < 0.05; **, *P* < 0.01; ***, *P* < 0.001.

**Supplementary Tables**

**Table S1. Expression patterns in HCC tissues and para-carcinoma tissues revealed in immunohistochemistry analysis**

| OTUB1 expression | Tumor tissue | | Para-carcinoma tissue | | *P* value |
| --- | --- | --- | --- | --- | --- |
|  | Cases | Percentage | Cases | Percentage | <0.0001*** |
| Low | 46 | 59.0% | 81 | 100% |  |
| High | 32 | 41.0% | 0 | - |  |

**Table S2. Relationship between OTUB1 expression and tumor characteristics in patients with HCC**

| Features | No. of patients | OTUB1 expression | | *P* value |
| --- | --- | --- | --- | --- |
|  |  | low | high |  |
| All patients | 78 | 46 | 32 |  |
| Age (years) |  |  |  | 0.1163 |
| <51 | 38 | 19 | 19 |  |
| ≥51 | 40 | 27 | 13 |  |
| Gender |  |  |  | 0.2084 |
| Male | 63 | 35 | 28 |  |
| Female | 15 | 11 | 4 |  |
| Cirrhotic nodule |  |  |  | 0.3610 |
| no | 11 | 7 | 4 |  |
| ≤3mm | 33 | 22 | 11 |  |
| >3mm | 34 | 17 | 17 |  |
| Tumor number |  |  |  | 0.0006*** |
| ≤1 | 63 | 43 | 20 |  |
| >1 | 15 | 3 | 12 |  |
| Grade |  |  |  | 0.8707 |
| II | 52 | 31 | 21 |  |
| III | 26 | 15 | 11 |  |
| AJCC Stage |  |  |  | 0.6551 |
| 1 | 51 | 31 | 20 |  |
| 2 | 27 | 15 | 12 |  |
| T Infiltrate |  |  |  | 0.6551 |
| T1 | 51 | 31 | 20 |  |
| T2 | 27 | 15 | 12 |  |
| Tumor recurrence |  |  |  | <0.0001*** |
| no | 31 | 27 | 4 |  |
| yes | 47 | 19 | 28 |  |
| Liver cirrhosis |  |  |  | 0.4478 |
| no | 10 | 7 | 3 |  |
| yes | 68 | 39 | 29 |  |
| HBsAg |  |  |  | 0.7345 |
| no | 11 | 7 | 4 |  |
| yes | 67 | 39 | 28 |  |
| HBcAb |  |  |  | 0.3002 |
| no | 8 | 6 | 2 |  |
| yes | 68 | 38 | 30 |  |
| TB（Total bilirubin） |  |  |  | 0.9139 |
| <17.1umol/L | 58 | 34 | 24 |  |
| ≥17.1umol/L | 20 | 12 | 8 |  |
| ALT |  |  |  | 0.0216* |
| <40u/l | 34 | 25 | 9 |  |
| ≥40u/l | 44 | 21 | 23 |  |
| ALB（Serum albumin） |  |  |  | 0.6580 |
| <4g/dl | 20 | 11 | 9 |  |
| 4g/dl≤ALB<5.5g/dl | 57 | 34 | 23 |  |
| ≥5.5g/dl | 1 | 1 | 0 |  |
| AFP |  |  |  | 0.4298 |
| <20ug/l | 23 | 12 | 11 |  |
| ≥20ug/l | 55 | 34 | 21 |  |
| GGT |  |  |  | 0.0395* |
| <40u/l | 22 | 17 | 5 |  |
| ≥40u/l | 56 | 29 | 27 |  |
